# Biosynthesis of the Biphenomycin Family of Potent Antibiotics

**DOI:** 10.1002/anie.202516156

**Published:** 2025-11-02

**Authors:** Elisabeth Strunk, Alfred Lobert, Tatiana Khorovich, Katia M. Guzman Lucio, René Richarz, Maximilian Hohmann, Paul M. D'Agostino, Tobias A. M. Gulder

**Affiliations:** ^1^ Chair of Technical Biochemistry Department of Chemistry and Food Chemistry Technical University of Dresden Bergstraße 66 01069 Dresden Germany; ^2^ Department of Natural Product Biotechnology Helmholtz Institute for Pharmaceutical Research Saarland (HIPS) Helmholtz Centre for Infection Research (HZI) and Department of Pharmacy at Saarland University PharmaScienceHub (PSH) Campus E8.1 66123 Saarbrücken Germany; ^3^ Biosystems Chemistry, Faculty of Chemistry Technical University of Munich Lichtenbergstraße 4 85748 Garching Germany

**Keywords:** Antibiotics, Biophenomycins, Biosynthesis, Enzymes, RiPPs

## Abstract

Peptide natural products are important molecules for the development of efficient drugs for human health applications. The biphenomycins are bacterial macrocyclic peptides characterized by unique *ortho*‐tyrosine (*o*Tyr) residues connected by biaryl linkages. Biphenomycins possess potent antibacterial activity against Gram‐positive pathogens at low doses with no eukaryotic toxicity. Despite their initial discovery in 1967, their biosynthetic pathway has remained elusive. Within this work, we identified the ribosomal biosynthetic origin of biphenomycins and elucidated all enzymatic maturation steps by in‐depth functional characterization in vivo and in vitro. Key steps include selective *ortho*‐hydroxylation events at two phenyl alanine residues catalyzed by a bifunctional multinuclear nonheme iron‐dependent oxidase yielding the *o*Tyr functionalities, biaryl cross coupling by a B12‐dependent radical SAM enzyme, amino acid side‐chain modifications by a highly regioselective arginase and by dedicated hydroxylases, as well as a stepwise proteolytic processing by a TldD‐type but self‐sufficient protease. These findings clarify the molecular basis of biphenomycin assembly, reveal unprecedented enzymatic dual functions, and provide the foundation for the targeted discovery of novel biphenomycins and for the development of bioengineering strategies to enhance yields and develop antibiotics with further increased potency, addressing the urgent need for new antimicrobial agents.

## Graphical abstract

The complete biosynthetic pathway of the biphenomycin antibiotics is elucidated, revealing a unique enzymatic machinery featuring a bifunctional MNIO, a regioselective arginase, a B12‐dependent rSAM for *C,C* coupling, hydroxylation enzymology, and a metalloprotease with dual‐specificity. These findings uncover new enzyme functions in RiPP biosynthesis and establish biocatalytic tools for future antibiotic discovery and engineering.

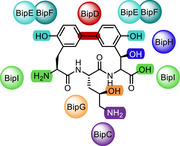

## Introduction

**Figure 1:**
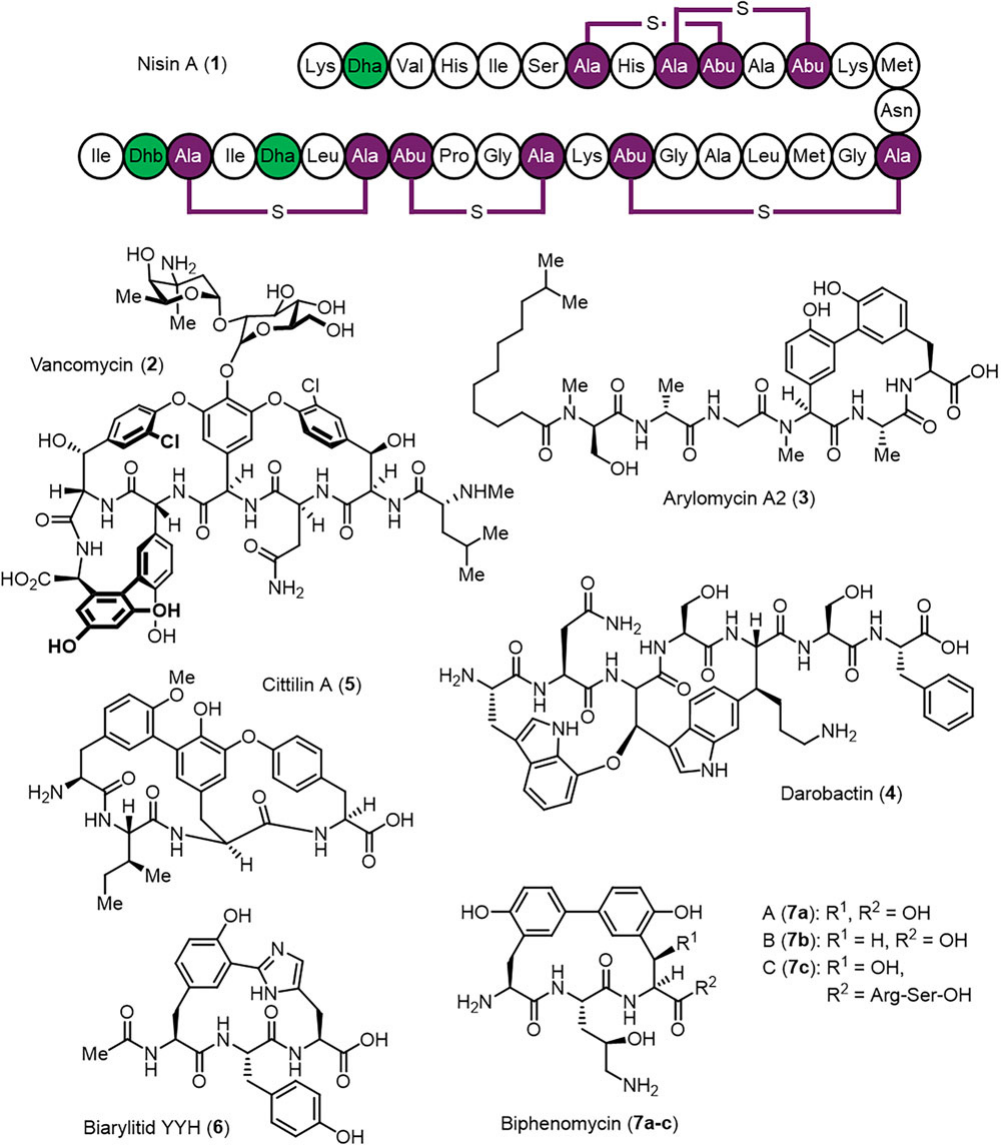
Structural diversity of cyclic bacterial peptides exemplified by nisin A (**1**), vancomycin (**2**), arylomycin A2 (**3**), darobactin (**4**), cittilin A (**5**), biarylitide YYH (**6**), and biphenomycins A‐C (**7a‐c**).

Natural products (NPs) are one of the most prolific sources for the discovery and development of innovative bioactive compounds for human health applications.^[^ ^1^, ^2^, ^3^ ^]^ Microbial peptides are among the structurally and functionally most diverse NP classes. Important examples include the lanthipeptide nisin A (**1**), which has been utilized in human health applications and as a food preservative,^[^ ^4^ ^]^ and the glycopeptide vancomycin (**2**), a clinical antibiotic of last resort (Figure 1).^[^ ^5^ ^]^

Nature has evolved two major strategies for the assembly of peptidic NPs. This can be exemplified by nisin A (**1**), a ribosomally synthesized and post‐translationally modified peptide (RiPP),^[^ ^6^, ^7^ ^]^ and vancomycin (**2**), produced by nonribosomal peptide synthetase (NRPS) machinery.^[^ ^8^ ^]^ In RiPP biosynthesis, small genes are translated into precursor peptides of approx. 20–110 amino acids (AAs), which consist of a leader peptide (LP), a core peptide (CP), and a potential follower peptide (FP).^[^ ^6^, ^7^ ^]^ In addition, RiPP biosynthetic gene clusters (BGCs) encode enzymes for structural tailoring. LP and FP serve as substrate recognition sequences for these maturases, which catalyze selective modifications at the CP. Typical modifications include alterations at individual AAs (by, e.g., oxygenation, alkylation, epimerization), formation of small heterocyclic elements (e.g., oxazoles, thiazoles), and backbone cross‐coupling reactions (e.g., *C*,*C*, *C*,*N*, *C*,*S*, *C*,*O*). RiPP biosynthesis is usually concluded by proteolytic excision of the CP and cellular export of the resulting mature NP.

For example, in the biosynthesis of **1**, the precursor peptide NisA is processed by the dehydratase NisB, which forms electrophilic dehydroalanine (Dha) and ‐butyrine (DhB) moieties from serine and threonine residues, respectively.^[^ ^9^ ^]^ The cyclase NisC subsequently catalyzes thioether formation by nucleophilic attack of the thiol functions of Cys residues to Dha/Dhb, leading to the formation of five macrocyclic units.^[^ ^10^, ^11^ ^]^ Transport and proteolysis are catalyzed by NisT^[^ ^12^ ^]^ and NisP,^[^ ^13^, ^14^ ^]^ respectively, thereby delivering final product **1** (Figure 1).

NRPSs are modular biosynthetic mega‐enzymes. Each module contains an adenylation, thiolation, and condensation domain responsible for selective AA activation, covalent tethering, and peptide bond formation, respectively.^[^ ^8^, ^15^ ^]^ Their catalytic interplay leads to a stepwise elongation of the nascent peptide while moving along the NRPS assembly line, with final release catalyzed by a thioesterase domain. Further optional catalytic domains within the NRPS modular organization can introduce structural changes, such as methylation, epimerization, or heterocyclization. In addition, tailoring enzymes can perform further functionalization reactions. For example, in the case of vancomycin (**2**), a 7‐modular NRPS delivers the hepta‐peptide precursor, which is tailored by halogenation, glycosylation, and three cross‐coupling reactions, the latter catalyzed by three dedicated cytochrome P450 enzymes (CYPs).^[^ ^16^, ^17^, ^18^ ^]^

Irrespective of their biosynthetic origin, many potent bioactive peptides, such as **1** and **2**, contain macrocylic structural elements. Macrocyclization by formation of *C*,*C─* or *C*,*O*,*C─*bonds is typically performed by CYP or radical SAM enzymes (rSAM). The corresponding structural features lead to significantly increased metabolic stability while also conferring highly defined three‐dimensional structures that facilitate strong NP‐target interactions.^[^ ^19^ ^]^ Small ring systems incorporating three AAs seem to be particularly privileged structural motifs. Such elements can not only be found in **2**, but in many peptide NPs. Examples include the potent signal peptidase inhibitor arylomycin A2 (**3**, NRPS‐derived, cross‐linking by CYP),^[^ ^20^, ^21^ ^]^ the BamA inhibitor darobactin (**4**, RiPP, rSAM),^[^ ^22^, ^23^ ^]^ the bicyclic cittilin (**5**, RiPP, CYP; here, a 2nd ring system containing only two AAs is additionally present),^[^ ^24^, ^25^ ^]^ and the biarylitides, such as YYH (**6**, RiPP, CYP).^[^ ^26^, ^27^, ^28^ ^]^

Another interesting group of macrocyclic peptide NPs are the biphenomycins A‐C (**7a‐c**).^[^ ^29^, ^30^ ^]^ Their ring structures consist of two rare *ortho*‐Tyr (*o*Tyr) units that are directly connected by a biaryl bond and are attached to a central, γ‐hydroxylated ornithine unit by peptide bonds. Biphenomycins A (**7a**) and B (**7b**) only differ with respect to the presence or absence of a β‐hydroxy group at the *C*‐terminal *o*Tyr.^[^ ^31^ ^]^ In biphenomycin C (**7c**), the *C*‐terminus is further extended by Arg‐Ser‐OH.^[^ ^32^ ^]^ The biphenomycins stand out due to their potent antibacterial activity against Gram‐positive bacteria such as *Staphylococcus aureus* at concentrations between 0.07–0.6 mg kg^−1^ ED_50_. Furthermore, **7a** exhibits no toxicity in mice after intravenous application of 0.5 g kg^−1^ body weight.^[^ ^33^ ^]^ However, biotechnological production of biphenomycins is hampered by very low production titers of approx. 0.1 mg L^−1^ for biphenomycin A (**7a**) and 0.01 mg L^−1^ for biphenomycin B (**7b**).^[^ ^33^ ^]^ Despite their initial discovery already in 1967 and their full chemical characterization in 1985,^[^ ^34^ ^]^ nothing was known about biphenomycin biosynthetic assembly at the start of this work. Their structures might derive of either RiPP or NRPS biosynthetic logic, with intriguing further open questions concerning the biosynthetic origin of *o*Tyr, the mechanism of biaryl coupling, the oxidative processing of the side chains, and potential other required maturation steps. Within this work, we thus set out to shed light on biphenomycin biosynthesis. Through the enzymatic characterization of the entire biosynthetic pathway, we provide a clear picture of biphenomycin biosynthesis. These results deepen our understanding of RiPP‐driven NP production and provide a foundation for efficient biotechnological production, for bioengineering approaches, and for the targeted discovery of novel congeners by bioinformatic approaches aiming to increase biological activities and hence potential application of this promising class of potent antibiotics.

## Results and Discussion

### Identification of a Putative Biphenomycin BGC (*bip*)

For the identification of the biosynthetic machinery catalyzing biphenomycin assembly, we retrieved the reported biphenomycin producer *Streptomyces griseorubiginosus* No. 43708^[^ ^33^ ^]^ from the International Patent Organism Depositary of the National Institute of Advanced Industrial Science and Technology (AIST) in Japan (accession ID FERM BP‐669). The strain was cultivated in SYM medium^[^ ^33^ ^]^ for 14 days at 30 °C and organic extracts of the supernatant were analyzed by LC‐HRMS (Figure 1a). Production of biophenomycins **7a** (main product) and **7b** was unambiguously validated.

**Figure 2:**
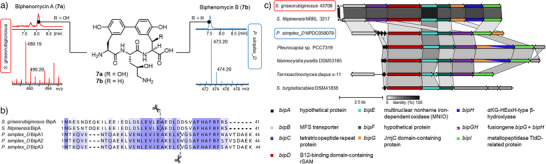
Production of biphenomycins and schematic representation of selected putative *bip* BGCs. a) LC‐HRMS analyses of extracts of the reported *S. griseorubiginosus* (production of **7a,b**) and the newly established producer *P. simplex* (exclusive production of **7b**) (Figure S1). b) Multiple sequence alignment of the precursor peptides BipA of the two known and the newly established producer of biphenomycins. Relevant GluC cleavage sites utilized for simplified LC‐HRMS/MS analysis are indicated by dashed lines and scissors. c) Genetic composition of a selection of putative biphenomycin BGCs across phylogenetically diverse organisms (graphics assembled using clinker).^[^ ^36^ ^]^

The genome of *S. griseorubiginosus* was sequenced at GATC Biotech AG using PacBIO SMRT sequencing technology. The resulting high‐quality draft genome with a total size of approx. 9.37 MBp was distributed on two contigs. These likely represent the bacterial chromosome (9.26 Mbp) and an additional plasmid (107 Kbp), based on the differences in GC content (71% versus 67.8%) and dramatically differing relative codon usage (81% versus 22%). Bioinformatic analysis of the genome using antiSMASH (version 8.0.1)^[^ ^35^ ^]^ revealed the presence of 28 putative NP BGCs (for a complete overview, cf. Figure S3). Only a single NRPS‐type BGC was detectable. However, this BGC only contains two adenylation domains, one of which predicted to activate Lys and the other one with no substrate prediction possible. Consequently, this made an NRPS origin of biphenomycins in *S. griseorubiginosus* highly unlikely. We, therefore, next analyzed the genome for the presence of a putative biphenomycin BGC based on RiPP biosynthetic logic. Screening of the genome for the most likely AA sequence of the hypothetical unmodified CP region of **7a–c**, Phe‐Arg‐Phe‐Arg‐Ser (FRFRS), confirmed it to be present eight times. Only a single one of these sequences was part of a protein coding sequence (CDS) based on RAST annotation. This FRFRS sequence fragment was found in a 41 AA long hypothetical protein, located at its *C*‐terminal end and directly followed by a stop codon. This would be in line with the general RiPP biosynthetic paradigm as it could represent a 41 AA long precursor peptide composed of a 36 AA LP followed by the anticipated 5 AA FRFRS CP. The corresponding gene was designated *Sg_bipA*. Bioinformatic analysis of the neighboring genes revealed the presence of eight further genes directly downstream within a single operon that based on BLAST homology searches might be involved in RiPP maturation. This includes genes putatively encoding an MFS transporter (*Sg_bipB*), a tetratricopeptide repeat protein (*Sg_bipC*), a B12‐binding domain‐containing rSAM (*Sg_bipD*), a member of the emerging class of multinuclear nonheme iron‐dependent oxidases (MNIO, *Sg_bipE*), a hypothetical protein (*Sg_bipF*), a JmjC domain‐containing protein (*Sg_bipG*), an α‐ketoglutarate‐dependent HExxH‐type β‐hydroxylase (*Sg_bipH*), and a TldD‐related metallopeptidase (*Sg_bipI*) (Figure 2b, Table S4).

To further substantiate our hypothesis that the candidate BGC *Sg_bip* identified in *S. griseorubiginosus* is indeed involved in biphenomycin production, we also sequenced the genome of the second reported producer strain, *S. filipinensis* NRRL 3217 using Illumina sequencing (GATC Biotech AG). Bioinformatic screening showed the presence of a virtually identical BGC *Sf_bip* composed of the same set of highly homologous genes in identical genetic organization (Figure 2b). Interestingly, when screening for related BGCs in publicly available genomes, we identified >1.000 similar pathways across phylogenetically diverse organisms not yet reported to produce biphenomycins (Figure 2b). We selected one of these potential production strains, *Peribacillus simplex_D* NPDC058078 (containing *Ps_bipA* candidate BGC with three highly conserved copies of BipA, Ps_BipA1‐3, Figure 2c), and retrieved the organism from NPDC (Natural Products Discovery Center). The strain was cultivated in M9 medium for 7 days at 30 °C and the organic extract of the supernatant analyzed for the presence of biphenomycins by LC‐HRMS/MS. To our delight, production of biphenomycin B (**7b**) was unambiguously validated (Figure 2a), further substantiated by in‐depth analysis of the MS/MS fragmentation patterns (Figure S2), strongly suggesting the putative *bip* candidate BGCs to be responsible for biphenomycin formation.

Based on general RiPP biosynthetic logic, structural maturation of the precursor peptide BipA is most likely to precede proteolytic release of biphenomycins. Preliminary functional predictions based on our BLAST homology searches on all enzymes in the *bip* BGC (Figure 2b, Tables S4 and S5) indicated the enzymes to potentially be involved in: AA side‐chain oxygenation by BipG and BipH; cross‐coupling by rSAM BipD; arginine side‐chain modification by BipC; proteolytic processing by BipI. No putative function was predictable for the MNIO enzyme BipE and the hypothetical protein BipF. However, as there was no obvious candidate for Phe hydroxylation, we anticipated one or both of these enzymes to be involved in this process.

To unambiguously elucidate the function of all maturases, we next turned our attention to their in‐depth functional interrogation using a combination of in vitro and in vivo approaches. Experiments were conducted using enzymes from both *Sg_bip* and *Ps_bip* BGCs. In vitro experiments were enabled with synthetic precursor peptides Sg_BipA and Ps_BipA1 (Shanghai Royobiotech Co.,Ltd). To facilitate LC‐HRMS/MS analysis of the products, GluC digestion was generally employed, leading to significantly shortened modified BipA sequences to be analyzed (relevant GluC cleavage sites indicated in Figure 2c).

### BipEF, a MNIO with Dual Function

A crucial and rare structural feature found in all biphenomycins are the two cross‐linked *o*Tyr residues. As outlined above, candidate enzymes to perform the required hydroxylation of Phe to deliver *o*Tyr were BipE and/or BipF. Based on sequence analysis, BipE was annotated as a putative MNIO. MNIOs are an emerging class of RiPP modifying enzymes that have recently received significant attention due to their diverse catalytic functions. Most characterized MNIO enzymes such as MbnBC, TglHI, or ChrHI, catalyze oxidative rearrangements at Cys‐rich precursor peptides resulting in cyclization or β‐carbon excision.^[^ ^37^, ^38^, ^39^ ^]^ Further studies have broadened the scope of potential modifications by *C*‐terminal amidation or oxidative conversion of Asp to an α‐keto acid by MovX and ApyHI, respectively.^[^ ^40^, ^41^ ^]^ While aromatic AA side‐chain oxygenation had not been reported prior to the start of our work (see conclusion section), we considered BipE to be a strong candidate to perform this function given the broad catalytic versatility of the few MNIOs investigated so far. The majority of MNIO enzymes form heterodimers with the subsequently encoded gene. While these partner proteins often lack a defining structural or recognizable catalytical domain and are therefore often overlooked in annotations, closer inspection frequently reveal the presence of a RRE (RiPP recognition element). This domain enables the binding to the precursor peptide while being essential for the assembly and catalytic activity of the functional holoenzyme.^[^ ^42^, ^43^ ^]^ We hypothesized that BipF could be the partner protein to the potential MNIO BipE.

To test our hypothesis, the corresponding genes *Sg_bipE* and *Sg_bipF* from *S. griseorubiginosus* No. 43 708 and their homologs *Ps_bipE* and *Ps_bipF* from *Peribacillus simplex_D* were cloned into a pACYC‐Duet vector system at MCS1 (*bipE*) and MCS2 (*bipF*), respectively. The expression construct was designed to produce BipE with an *N*‐terminal His_6_‐ or His_6_‐MBP‐tag for downstream affinity‐based protein purification, while BipF was devoid of an additional purification‐tag fusion. Recombinant protein production was carried out in *Escherichia coli* BL21(DE3) and the desired protein was purified by Ni‐NTA or MBP affinity chromatography. SDS‐PAGE analysis confirmed the presence of both, Sg_BipE/Ps_BipE and Sg_BipF/Ps_BipF, in the protein elution fraction (Figures S4 and S5). This confirmed the anticipated formation of a BipEF heterodimer.

**Figure 3:**
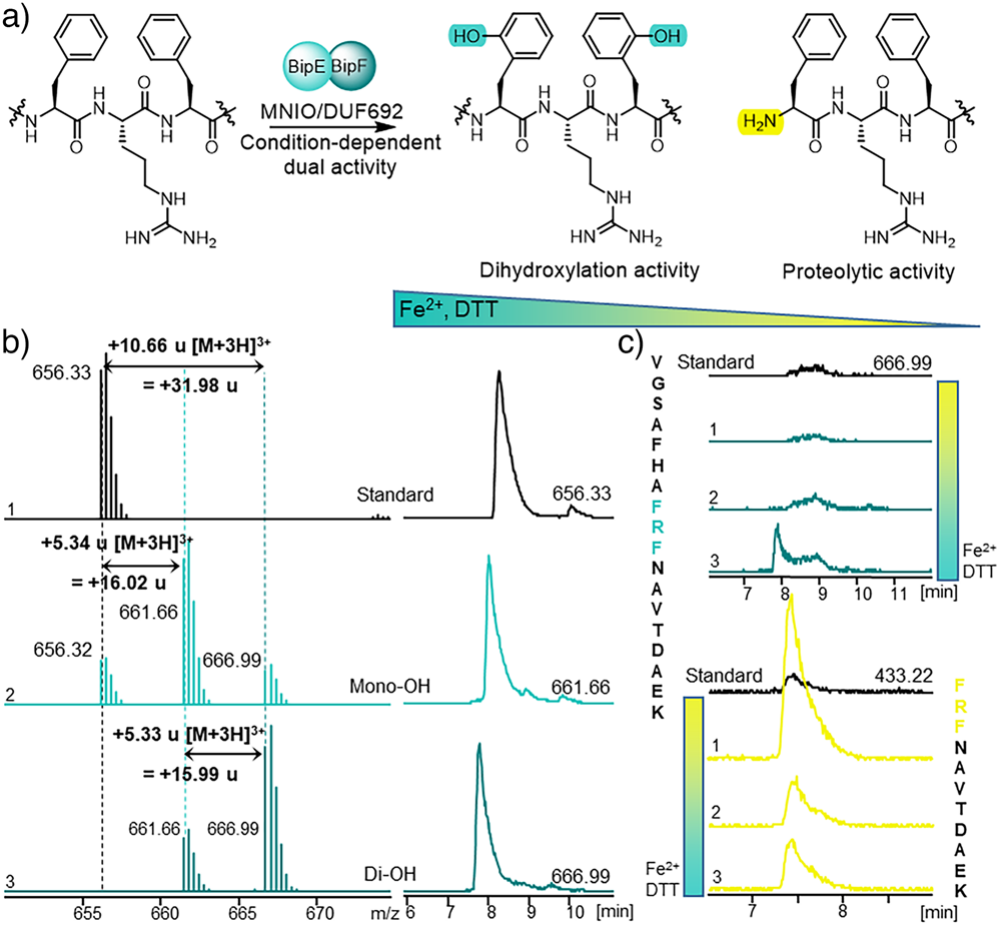
Enzymatic activity of Sg_BipEF and Ps_BipEF. a) Enzymatic reaction of Sg_BipEF/Ps_BipEF at the CPs Sg_BipA/Ps_BipA1, demonstrating a bifunctional activity depending on the assay conditions. Double hydroxylation of the Phe residues occurred when 5 mM DTT and 1 mM Fe^2+^ were present; *N*‐terminal proteolytic processing prevailed without these additives. b) HRMS (left) and HPLC (right) EIC analysis of the expected fragments after the corresponding mass change with Ps_BipA1 after GluC digest with unmodified Ps_BipA1 as a standard (1); mono‐ (2) and di‐hydroxylated (3) product of Ps_BipA1. c) EIC analysis of GluC‐digested fragments at their expected mass; di‐hydroxylated CP (blue) and *N*‐terminal cleavage product (yellow). Shown are standard and assays in the absence of additives (1), upon addition of 5 mM DTT (2), or of 5 mM DTT and 1 mM Fe^2+^ (3).

Enzymatic assays were conducted in parallel with purified Sg_BipEF and Ps_BipEF using the unmodified precursor peptides Sg_BipA and Ps_BipA1, respectively. Assays were performed in Tris buffer (50 mM, pH 8.0) with 20 µM enzyme and supplemented with ammonium iron(II) sulphate as source of Fe^2+^ and dithiothreitol (DTT) as reducing agent. LC‐HRMS/MS analysis of the assays after GluC digestion indeed revealed the anticipated hydroxylation at both Phe residues of the CP (Figure 3b), as clearly evident from detection of products with the respective increase in molecular mass by +16 Da (mono‐) or +32 Da (di‐hydroxylation). Further MS/MS analysis confirmed the hydroxy groups to be located at the two Phe residues of the CP of Sg_BipA and Ps_BipA. The mono‐hydroxylated product constitutes a mixture of both possible regioisomers, revealing that initial hydroxylation can occur on either Phe with no apparent regioselectivity (Figures S4 and S5).

In addition to the hydroxylation activity, we surprisingly detected a second function of BipEF. In the absence of Fe^2+^ and DTT, proteolytic cleavage at the *N*‐terminus of the CP was observed. The enzymatic activity therefore shifted from double hydroxylation with DTT and Fe^2+^ to exclusive proteolytic cleavage in the absence of additives (Figures 3C and S6 and S7). BipEF thus not only catalyzed a remarkable dihydroxylation sequence but also condition‐specific, selective proteolysis. It thus constitutes the first MNIO enzyme with such dual function.

### BipC, a Highly Regioselective Arginase

In our BLAST analysis, we discovered that BipC belongs to the UPF0489 superfamily, which is related to the arginase family. BipC also contains the characteristic Mn^2+^ binding motifs (EEH*x*E, D*x*H*x*D and DIDLD; Figure S8) found in arginases. Little is known about arginases in RiPP biosynthesis as only few are thoroughly characterized. OspR is the most extensively studied RiPP arginase and is involved in landornamide A biosynthesis.^[^ ^44^ ^]^ OspR displays a high substrate tolerance with respect to precursor peptide sequences as well as Arg‐enriched precursor peptides. To confirm BipC to function as an arginase, we cloned and expressed *Sg_bipC* as well as *Ps_bipC* into the pMAL‐c5x/c6t vector system to produce MBP‐tagged fusion proteins in *E. coli* BL21 (DE3), facilitating protein purifications on MBP‐Trap columns (Figures S9 and S10). After successfully obtaining purified Sg_BipC and Ps_BipC proteins, we conducted in vitro assays with 20 µM enzyme and supplementation of the reactions with 1 mM MnCl_2_ as a source of Mn^2+^ as well as 5 mM DTT.

**Figure 4:**
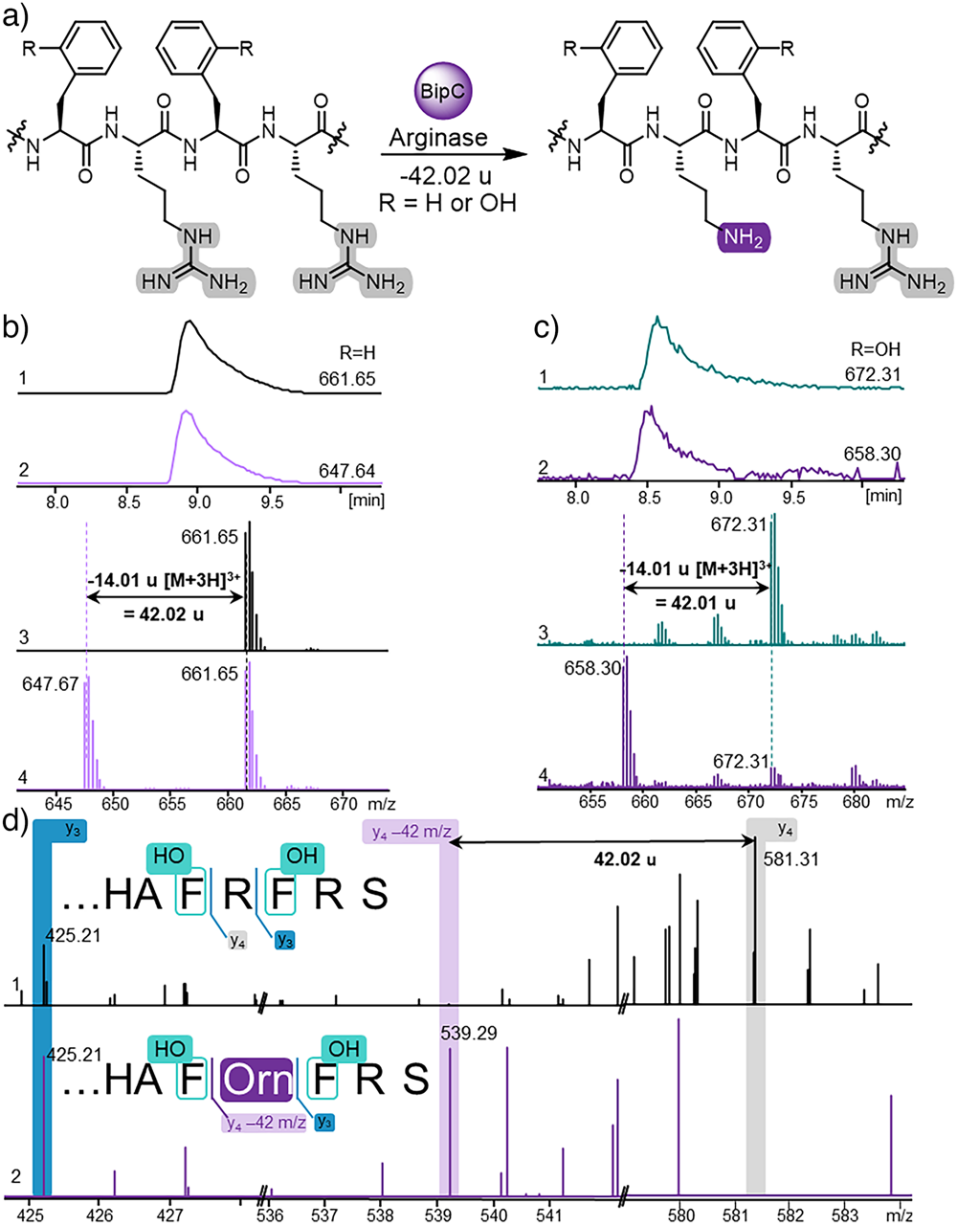
Elucidation of the catalytic activity of Sg_BipC and Ps_BipC, exemplarily depicted for Sg_BipC. a) Regioselective hydrolysis of Arg to Orn at the CP of Sg_BipA catalyzed by Sg_BipC. b) LC‐HRMS data of Sg_BipC catalyzed reaction, displaying the EIC of GluC digested fragments for their calculated mass with umodified Sg_BipA standard (1,3) and modified Sg_BipA after hydrolysis of Arg to Orn (2,4) with a corresponding mass loss of ‐42 Da. c) LC‐HRMS data of enzymatic reaction of Sg_BipC on the previously hydroxylated Sg_BipA, displaying the EIC of the calculated mass after GluC digestion of Sg_BipA after action of Sg_BipEF (1,3) with a mass gain of +32 Da and further modified Sg_BipA after Sg_BipC reaction (2,4) with the corresponding mass loss of ‐42 Da. d) HRMS/MS fragmentation analysis of unmodified (top) and processed (bottom) Sg_BipA after GluC digestion. Strict regioselectivity was confirmed by identical y_3_‐ions and differing y_4_‐ions (loss of 42 Da validating Arg to Orn transformation).

Initial in vitro assays were conducted with Sg_BipC using the unmodified precursor peptide Sg_BipA as a substrate. Analysis of the GluC‐digested products revealed the expected mass loss of ‐42 Da, corresponding to the transformation of Arg to Orn by deguanidination (Figure 4b). Despite the presence of two Arg residues in Sg_BipA in close proximity and only separated by a Phe residue, the hydrolytic activity of Sg_BipC was exclusively observed at the central Arg flanked by the two Phe residues. This was evident from MS/MS analysis: y‐fragments with the mass loss of 42 Da were exclusively observed starting with the y_3_‐ion (Figure 4c). These results establish Sg_BipC as a highly regioselective enzyme that is capable of distinguishing between two Arg residues in close proximity.

Interestingly, Sg_BipC not only processed unmodified Sg_BipA (Figure 4a, R═H) but also the mono‐ or dihydroxlyated Sg_BipA congeners (R═OH) resulting from the catalytic activity of Sg_BipEF outlined above (Figure S9). However, while Ps_BipC showed identical site‐selectivity when compared to Sg_BipC, a decreased substrate promiscuity was observed; the arginase reactivity was exclusively detected when presenting the enzyme with di‐hydroxylated Ps_BipA1, with little to no turnover of the unmodified precursor peptide (Figure S10). This strongly indicated Ps_BipC and Sg_BipC to preferentially accept di‐hydroxylated Ps_BipA1/Sg_BipA and hence to likely act after Ps_BipEF/Sg_BipEF in biphenomycin biosynthesis. This was corroborated by the observation that Sg_BipEF does not show any turnover after Arg to Orn hydrolysis by Sg_BipC.

### BipG, a αKG‐ and Fe(II)‐Dependent Ornithine Hydroxylase

BLAST analysis of BipG showed a JmjC domain containing protein. The JmjC domain is associated with many α‐ketoglutarate (KG)‐ and Fe(II)‐dependent oxygenases that catalyze a variety of oxidations. JmjC oxygenases are generally divided into two major classes based on their function: de‐methylation versus hydroxylation.^[^ ^45^ ^]^ Given substrate and product structures in case of biphenomycin biosynthesis, we predicted BipG to function as a hydroxylase targeting either the Arg or Orn residue within the precursor peptide. To prove this hypothesis, *Sg_bipG* and *Ps_bipG* were cloned into the pMAL‐c5x/c6t vector, proteins expressed in *E. coli* BL21 (DE3), and purified using MBP‐Trap columns (Figures S11–S13).

**Figure 5:**
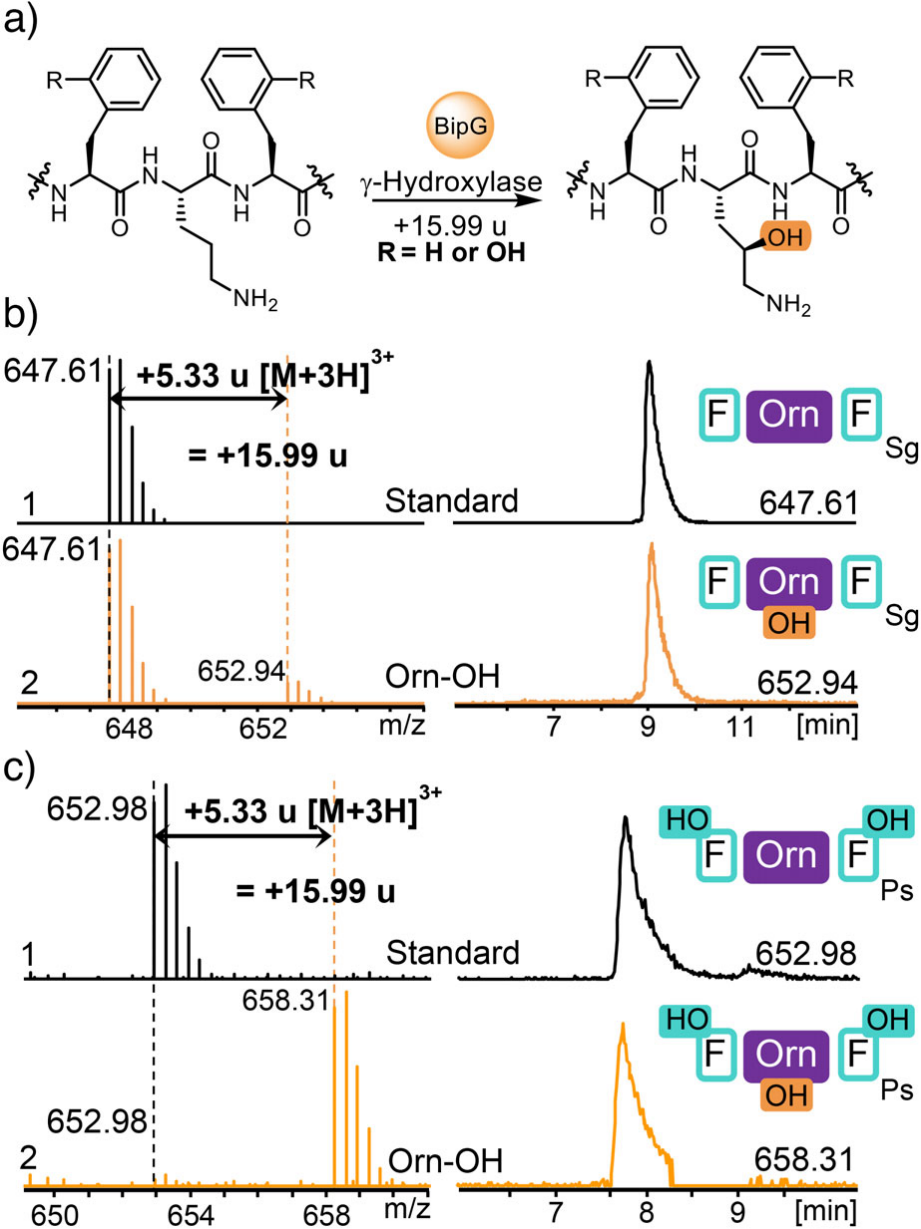
Enzymatic activity of Sg_BipG/Ps_BipG. a) Function of Sg_BipG/Ps_BipG catalyzing the γ‐hydroxylation of Orn in the CP of Sg_BipA/Ps_BipA1. b) LC‐HRMS analysis of deguanidinated Sg_BipA standard (1, expected mass 647.61 [M + 3H]^3+^) and the hydroxylated product (2, 652.94 [M + 3H]^3+^) with a mass gain of 16 Da. c) LC‐HRMS EIC analysis of di‐hydroxylated and deguanidinated Ps_BipA1 standard (1, 652.98 [M + 3H]^3+^) and the CP modified by BipG‐catalyzed hydroxylation (2, 658.31 [M + 3H]^3+^) with a mass gain of 16 Da.

To assess catalytic activity of Sg_BipG and Ps_BipG, a range of substrates was tested in in vitro assays, including the respective cognate unmodified precursor peptides (Phe‐Arg‐Phe) and the mono‐ (*o*Tyr‐Orn‐Phe) and di‐hydroxylated (*o*Tyr‐Orn‐*o*Tyr) modified CPs. The CP Phe‐Orn‐Phe lacking prior hydroxylation was only available for Sg_BipA due to the broader substrate tolerance of Sg_BipC that did not require prior dihydroxylation, as detailed above. Using this approach, we aimed to confirm enzymatic activity and timing of BipG catalysis in biphenomycin biosynthesis. The assays revealed that hydroxylation activity required the presence of Orn, with no detectable hydroxylation at Arg‐containing substrates (Figures 5a and S13). Interestingly, Orn hydroxylation activity of BipG was not dependent on di‐hydroxylation of Phe residues to *o*Tyr: all Orn‐containing substrates were readily transformed into the respective Orn side‐chain hydroxylated congeners, exemplarily shown for modified Sg_BipA containing Phe‐Orn‐Phe (Figures 5b and S12) and modified Ps_BipA1 containing *o*Tyr‐Orn‐*o*Tyr (Figures 5C and S13). These results validated Sg_BipG and Ps_BipG to act as selective γ‐Orn hydroxylases in biphenomycin maturation. These enzymes might be valuable starting points to engineer Orn hydroxylases as tools for functionalization of diverse Orn‐containing CPs.

### BipD, a B12‐Containing rSAM Catalyzing Biaryl‐Bond Formation

BLAST analysis predicted BipD to be a B12‐binding domain‐containing rSAM enzyme. B12‐dependent rSAMs are known to catalyze a range of reactions in RiPP biosynthesis, with the majority described to function as methyltransferases, such as PoyC or TsrM.^[^ ^46^, ^47^ ^]^ Other B12‐dependent rSAMs, such as ThnL or OxsB, are catalyzing complex reactions such as *C,C*‐ and *C,S* bond formation or oxidative ring contractions.^[^ ^48^ ^]^ Despite the broad catalytic range, the functional diversity of B12‐dependent rSAMs in RiPP biosynthesis remains largely unexplored.^[^ ^49^, ^50^ ^]^ As no methylation occurs in biphenomycin biosynthesis, we anticipated Sg_BipD and Ps_BipD to catalyze the biaryl bond formation between the two *o*Tyr residues resulting from action of Sg_BipEF/Ps_BipEF, thereby completing formation of the prototypical macrocycle in biphenomycins. To enable functional studies, we cloned *Sg_bipD* and *Ps_bipD* into the vectors pMAL‐c5x and pHis8‐TEV, resulting in constructs with an *N*‐terminal MBP‐ or a His_8_‐Tag fusion, respectively. These constructs were individually introduced into *E. coli* BL21 (DE3) together with helper plasmid pBAD1030S::*btuCEDFB*, which enables enhanced cellular uptake of B12 in *E. coli*.^[^ ^51^ ^]^ Protein expressions were supplemented with cyanocobalamin (CNCob) as well as ammonium iron(II) sulphate and cysteine to support [4Fe‐4S] cluster assembly. The fusion protein was purified by either Ni‐NTA affinity chromatography (His‐tag) or MBP‐Trap (MBP‐tag). It is important to note that co‐expression with the helper plasmid was crucial as we observed significant reduction in protein solubility when it was absent, caused by limited availability of B12 in the recombinant host. The incorporation of B12 during expression is known to increase solubility of rSAMs drastically.^[^ ^51^ ^]^ These effects were consistent for both proteins, Sg_BipD and Ps_BipD (Figures S18 and S19).

To characterize the identity of the B12 cofactor in Sg_BipD and Ps_BipD, we expressed and purified the corresponding proteins in the absence of light and released the protein‐bound B12 by addition of 100 mM H_2_SO_4_ for acid‐promoted protein denaturation. LC‐HRMS/MS of the resulting supernatant revealed a predominant presence of hydroxocobalamin (HOCob) and smaller amounts of adenosylcobalamin (AdoCob), while methylcobalamin (MeCob) was practically absent in Sg_BipD and Ps_BipD (Figure 5b). To enable further characterization of Sg_BipD and Ps_BipD, we reconstituted the [4Fe‐4S] cluster. Anaerobic reconstitution was conducted by incubation of the purified enzymes Sg_BipD and Ps_BipD with an eightfold excess of ammonium iron(II) sulphate and Na_2_S, followed by removal of unbound iron sulfide. UV–vis spectra showed absorption bands at 360 and 550 nm, confirming the presence of bound cobalamin in Sg_BipD and Ps_BipD (Figure 5c).^[^ ^52^ ^]^ Formation of the [4Fe‐4S]^2+^ cluster was underlined by the characteristic appearance of new UV‐bands at 300 nm and 420–470 nm upon reconstitution.^[^ ^47^ ^]^ The subsequent loss of these absorption maxima upon the reduction of the enzymes with Ti(III) citrate to [4Fe‐4S]^1+^ supported the redox‐sensitive nature of the cluster. Altogether, these data support the successful assembly of catalytically active B12‐containing rSAM enzymes. To confirm enzyme activity, we assessed their efficiency in SAM cleavage assays, the initial step in rSAM catalysis. Assays were conducted with 20 µM enzyme in Tris buffer (50 mM, pH 8.0) containing the substrate, 1 mM SAM, 10 mM DTT, and a reducing system. The selection of the reducing system is highly important due to the reduction of the [4Fe‐4S]^2+^ cluster into the catalytically active state of [4Fe‐4S]^1+^. Therefore, we conducted enzyme assays to identify the best reducing system with highest SAM cleavage rate by employing: dithionite (DTH), methyl viologen (MV)/NADPH, flavodoxin/flavodoxin reductase/NADPH, and Ti(III) citrate, with the latter delivering best results (Figure 5d and S14–S17).

**Figure 6:**
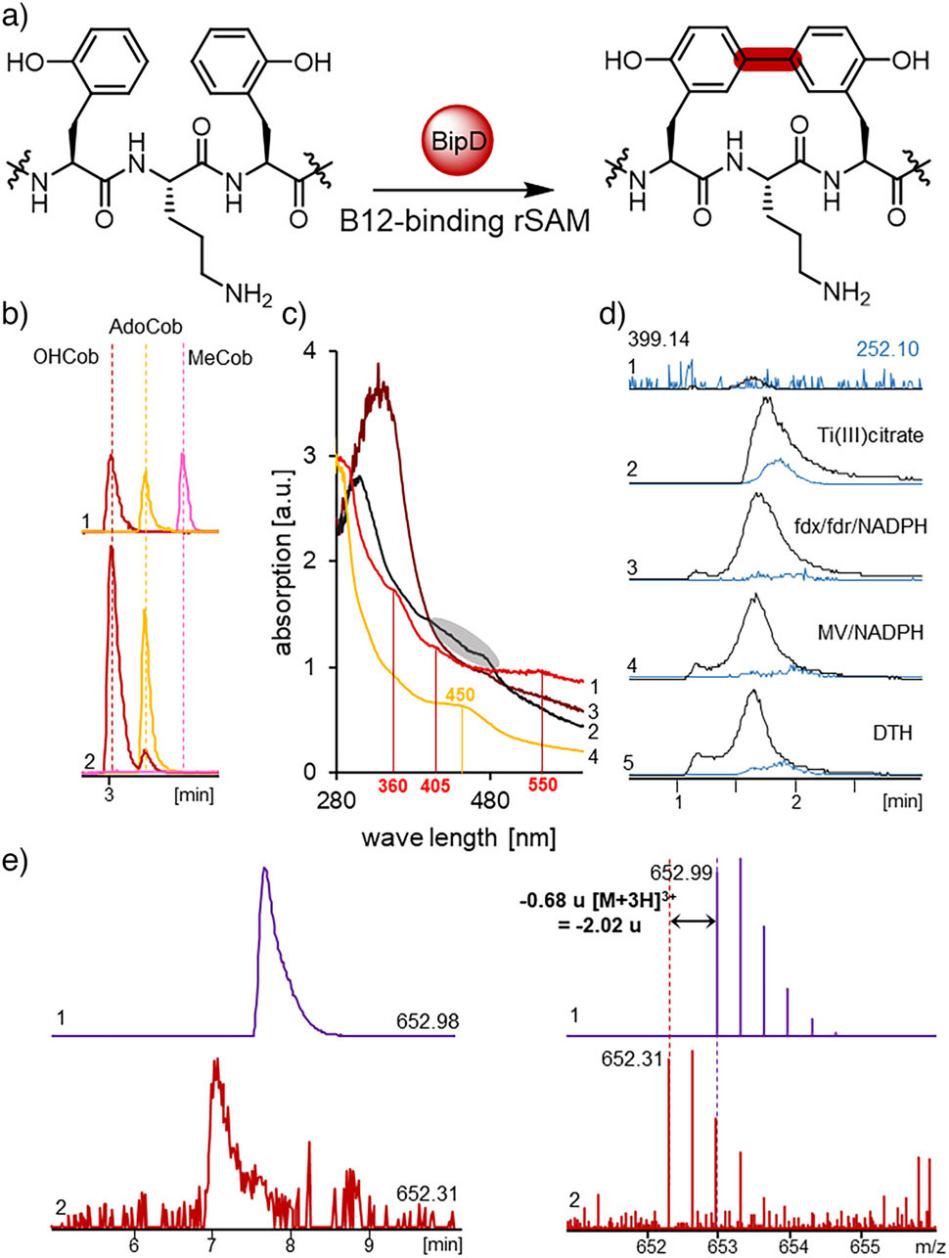
Functional evaluation of rSAMs Sg_BipD and Ps_BipD. **a)** Putative BipD‐catalyzed *C*,*C*‐biaryl coupling reaction. **b)** Determination of B12 species bound to Sg_BipD (2, 56 µM) denatured with 100 mM H_2_SO_4_ by comparison to a standard (1, mix of HOCob, AdoCob and MeCob) and LC‐HRMSLC analysis. c) UV–vis spectra of Sg_BipD (20 µM), (1) directly after isolation (red), (2) after reconstitution (black), (3) after reconstitution followed by treatment with 12.5 mM Ti(III) citrate (brown), and (4) for comparison the reconstituted protein without cobalamin supplementation (yellow). The absorption of cobalamin directly after protein isolation is slightly visible at 360 nm, 405 nm and 550 nm (1). After reconstitution, UV signals of the [4Fe‐4S]^2+^ cluster and of reduced cob(II)alamin around 420–470 nm are slightly visible (2, grey highlight). For comparison, the reconstituted Sg_BipD without cobalamin, shows a strong absorption at 450 nm (4), corresponding to the [4Fe‐4S]^2+^ cluster. d) SAM cleavage assays exemplarily shown for Ps_BipD. Depicted are an assay without SAM as standard (1) and screening reactions with different reduction systems: 15 mM Ti(III) citrate (2), 20 µM fdx/5 µM fdr/2 mM NADPH (3), 1 mM MV/2 mM NADPH (4), 1 mM DTH (5). LC‐HRMS analysis depicts EICs for SAM (black, 399.14 [M + H]^+^) and the 5′‐dAdoH (blue, 252.10 [M + H]^+^) cleavage product.

To characterize the function of BipD in biphenomycin maturation, we conducted co‐expression experiments together with the genes encoding enzymes thought to precede rSAM‐catalyzed biaryl formation, namely *bipEF* and *bipC*. Genes *Ps_bipEF*, *Ps_bipC*, and *Ps_bipD* were assembled into construct pACYC‐Duet::*Ps_bipDEFC* without any additional tags. The construct was transferred into *E. coli* BL21 (DE3) and co‐expressed with His_8_‐tagged precursor peptide Ps_BipA1 and the helper plasmid pBAD1030S::*btuCEDFB*, with final protein purification on Ni‐NTA columns (Figure S24). GluC digestion of the eluted Ps_BipA1 and analysis of the cleavage product revealed the expected structural changes introduced by Ps_BipEF (*o*Tyr formation) and Ps_BipC (hydrolysis of Arg to Orn) together with an additional mass shift of ‐2 Da, consistent with the expected *C,C* cross‐coupling reaction (Figures 6e and S25 and S26). These results confirmed Ps_BipD to be responsible for the biaryl bond formation in biphenomycin biosynthesis.

### BipI, a Metalloprotease with *N*‐, and *C*‐Terminal Proteolytic Activity

In RiPP biosynthesis, the final maturation step is usually the release of the CP from the adjacent LP and FP. As described above, *N*‐terminal cleavage activity was detected for Sg_BipEF/Ps_BipEF. However, the mechanism underlying *C*‐terminal cleavage remained unresolved. A strong candidate for this reactivity was the putative TldD‐related metalloprotease BipI. TldD metalloproteases are known as RiPP maturases that usually form a heterodimer with TldE. One of the best characterized TldD/E metalloproteases is involved in the *N*‐terminal processing of McbA to form microcin B12 in *E. coli*.^[^ ^53^ ^]^ Analysis of crystal structures has shown that TldD and TldE only share 18% identity while still possessing similar folds. The formation of a TldD/E heterodimer improves the stability of both proteins and is required for enzymatic activity. However, only TldD contains the metal‐binding domain HE*xx*H required for enzymatic activity. The proteolytic cleavage mechanism was described as channel‐like pencil sharpener, where the LP is being cleaved into smaller AA sequences until steric hindrance within the modified CP region (heterocycles in McbA) is preventing further cleavage.^[^ ^54^ ^]^ Sequence analysis of Sg_BipI and Ps_BipI revealed the conserved HE*xx*H motif typical in TldD‐family zinc metalloproteases. Notably, unlike the canonical TldD/TldE systems, no candidate for a gene encoding a TldE‐like partner could be identified in the vicinity of the *bip* BGC. While TldD enzymes alone are capable of retaining proteolytic activity, efficiency and stability are diminished without TldE.^[^ ^55^ ^]^ To enable enzymatic activity elucidation of BipI, we individually cloned *Sg_bipI* and *Ps_bipI* into the pMAL‐c5x vector for expression in *E. coli* BL21 (DE3). The MBP‐fusion protein was purified on MBP‐Trap columns (Figures S22 and S23). In vitro enzyme assays were conducted with 50 µM enzyme in Tris buffer (50 mM, pH 7.5) supplemented with 125 mM NaCl, 1 mM MnCl_2_ and 5 mM DTT as reducing agent.

We evaluated a range of substrates, starting with the unmodified precursor peptides Sg_BipA and Ps_BipA1. TldD/E metalloproteases involved in RiPP maturation usually require complete post‐translational modification of the CP and lack enzymatic activity on unmodified substrates.^[^ ^54^ ^]^ Remarkably, both Sg_BipI and Ps_BipI catalyzed *N*‐ and *C*‐terminal cleavage of the unmodified Sg_BipA and Ps_BipA1, respectively, releasing Phe‐Arg‐Phe (Figure 6a). Thorough inspection of the LC‐HRMS data showed the presence of the released LPs (Figure 6a, 1) and FPs (2). Furthermore, the intact sequence of CP‐FP was still detectable, attesting to cleavage at the *N*‐terminal region (3). However, a product consisting of LP‐CP was not found (4). This reveals that Sg_BipI and Ps_BipI process the precursor peptides in a progressive manner, where in a first step the *N*‐terminal LP is removed, followed by a second step for the cleavage of the *C*‐terminal FP (5).

To further explore potential substrate tolerance of Sg_BipI and Ps_BipI, we tested additional precursor peptides Sg_BipA and Ps_BipA1 with varying degrees of modifications introduced by the maturases described above (Figure 6c). The following CP alterations were tested, exemplarily shown for catalytic action of Sg_BipI: unmodified Sg_BipA with FRF as a positive control (Figure 6c, 1); unmodified Ps_BipA with FRF (2), Sg_BipA with F‐Orn‐F (3), Ps_BipA with *o*Tyr‐Orn‐*o*Tyr (4), Sg_BipA with artificial Y‐Orn‐Y (5), and truncated FRFRS devoid of any LP (6). In all cases, the released CPs were detected, along with the LP and FP cleavage products (Figures S20 and S21). It is important to note that Sg_BipI and Ps_BipI also efficiently processed peptides in the Ps_BipA and Sg_BipA sequence context, respectively (Figure 6c, 2, and 4 and S20 and S21). This is particularly interesting as other Sg_Bip‐ and Ps_Bip‐enzymes were not catalytically active on Ps_BipA1 or Sg_BipA substrates, respectively.

The observed catalytic activity of Sg_BipI toward the truncated FRFRS peptide (Figure 6c,5) excludes the necessity of a recognition motif within the LP of Sg_BipA. As the FP sequences in Sg_BipA versus Ps_BipA1 are very different, yet both, Sg_BipI and Ps_BipI, can process either sequence, substrate recognition by interactions with the FP are also unlikely. Therefore, we propose that Sg_BipI and Ps_BipI process the precursor peptide in a progressive cleavage mechanism similar to the “pencil sharpener” model of TldD/E enzymes, which is constrained by steric hindrance of the CP. This observation is consistent with a sequential processing model in which the initial step consists of the *N*‐terminal cleavage by BipI or a different proteolytic enzyme, such as BipEF, followed by a final *C*‐terminal cleavage event to release the mature RiPP. The lack of detectable fragments consisting of the LP and CP with a cleaved FP further supports the hypothesis of a stepwise processing mechanism. Biphenomycin C (**7c**) would hence result from *N*‐terminal cleavage of the LP and lack of the second, *C*‐terminal processing step.

Our results establish Sg_BipI and Ps_BipI as unique dual‐specificity TldD‐like metalloproteases capable of catalyzing both, *C*‐ and *N*‐terminal processing of the CP. This is combined with a very broad substrate tolerance, highlighting the potential of BipI for versatile applications in RiPP pathway engineering.

## Conclusion

Our work elucidates the full biosynthetic assembly of the biphenomycin family of potent antibiotics. Starting with the unmodified precursor peptide **8**, the MNIO BipEF initially hydroxylates the two Phe residues in the CP region to deliver **9** containing the prototypical biphenomycin *o*Tyr residues (Figure 8). The arginase BipC selectively hydrolyses the Arg residue located between the two *o*Tyr units to give **10**, with no deguanidation observed at the Arg if located in the FP. The biaryl cross link is subsequently installed by the B12‐dependent rSAM BipD yielding **11**, setting the stage for selective γ‐hydroxylation catalyzed by BipG to furnish **12**. From **12**, biosynthesis diverges dependent on the presence (in *S. griseorubiginosus*) or absence (in *P. simplex_D*) of the αKG‐HExxH‐hydroxylase BipH. In *P. simplex_D*, proteolytic processing by the metalloprotease BipI directly and exclusively delivers biphenomycin B (**7b**). In *S. griseorubiginosus*, BipH‐mediated hydroxylation provides intermediate **13**, which upon BipI‐catalyzed removal of the LP results in biphenomyin C (**7c**). Upon further proteolytical processing of **7c** by BipI, biphenomycin A (**7a**) is ultimately released (Figure 8).

**Figure 7:**
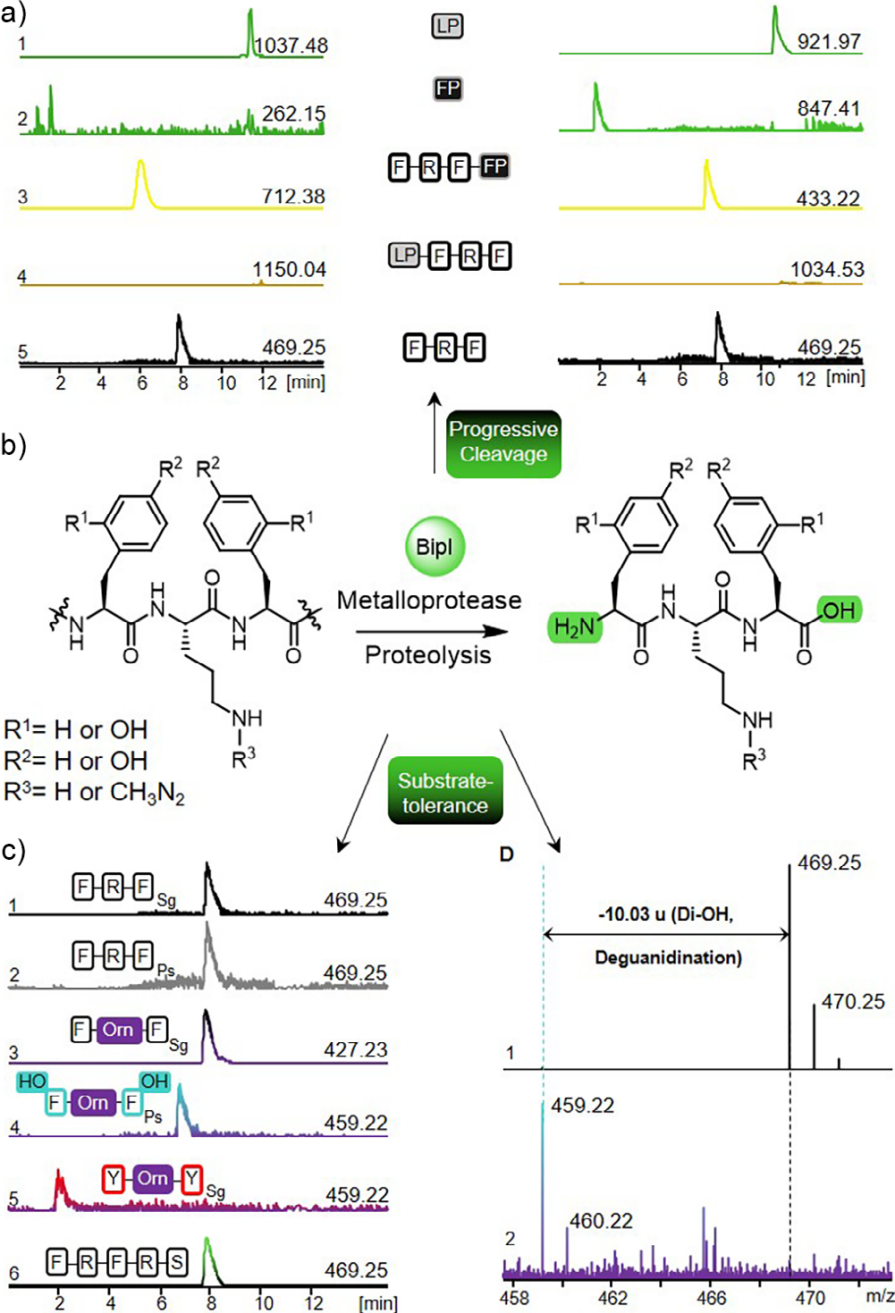
Functional analysis of Sg_BipI and Ps_BipI. a) LC‐HRMS analysis of the progressive cleavage of the unmodified precursor peptides Sg_BipA (left) and Ps_BipA1 (right) by Sg_BipI and Ps_BipI, respectively. Depicted are EICs for the expected masses resulting from the LP (1), the FP (2), CP still bound to FP (3), CP still bound to LP (4, not detected), and the fully excised CP Phe‐Arg‐Phe (5). b) Proteolytic reaction catalyzed by Sg_BipI and Ps_BipI. **c)** LC‐HRMS analysis of substrate screening exemplarily shown for Sg_BipI. Depicted are EICs of the expected excised CP from unmodified Sg_BipA (1), unmodified Ps_BipA1 (2), the Sg_BipC modified, Orn‐containing Sg_BipA (3), Ps_BipEF and Ps_BipC modified, Orn and *o*Tyr‐containing Ps_BipA1 (4), artificial Tyr‐Orn‐Tyr CP (5), and exclusive *C*‐terminal cleavage of the synthetic FRFRS CP bound to the FP Sg_BipA (6). **d)** HRMS/MS spectra of released CP from unmodified Ps_BipA1 (1, expected mass 469.25 [M + H]^+^) and di‐hydroxylated, deguanidinated CP of Ps_BipA1 (2, 459.22 [M + H]^+^).

Overall, the biphenomycin biosynthetic pathway described in this work features several maturation steps catalyzed by unique and biotechnologically valuable enzymes, including the bifunctional BipEF, which beyond the depicted dihydroxylation reactivity also possesses condition‐dependent proteolytic activity, the highly regioselective arginase BipC, two oxidative processing enzymes BipG and BipH, the rSAM BipD connecting the two *o*Tyr residues by *C*,*C*‐cross coupling, and the self‐sufficient and bifunctional metalloprotease BipI with *N*‐ and *C*‐terminal proteolytic activity (Figure 7).

**Figure 8:**
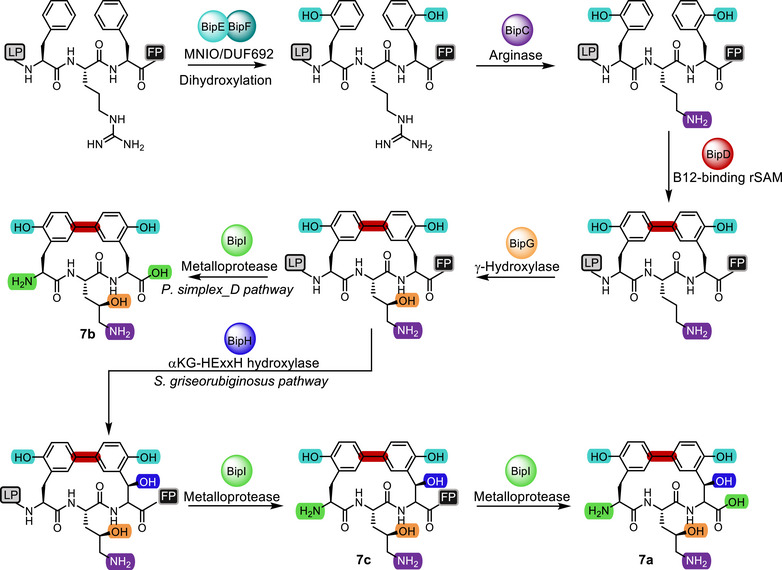
Full biosynthetic assembly of biphenomycins A‐C (**7a‐c**) from precursor peptide **8**.

In parallel to our work, van der Donk and coworkers explored the functional diversity of uncharacterized MNIOs, specifically from *Peribacillus simplex* BE23.^[^ ^56^ ^]^ In their study, they nicely showed that the MNIO PbsCD performs two *ortho* hydroxylation reactions at an FRF motif of an adjacent precursor peptide, leading to two *o*Tyr residues. The precursor peptide is further modified by an arginase PbsE and a cross‐link between the *o*Tyr units established by an rSAM PbsB with further precursor peptide hydroxylation by PbsQ. The characterized enzymatic functions are thus reminiscent of the biphenomycin pathway described here, yet full proteolytic processing of the modified precursor peptide to biphenomycins or related small molecules was not observed. As shown within our work, this can be achieved by the dual function of BipEF and particularly by the *N*‐ and *C*‐terminal proteolytic activity of BipI. Additionally, comparison between *P. simplex_D* and BE23 support the conservation of function between orthologs, whereas interspecies comparison (e.g., to *Streptomyces*) reveals distinct variations in activity and substrate scope, emphasizing the biosynthetic diversity across taxa. Together, the work by van der Donk^[^ ^56^ ^]^ and our study provide the basis for the targeted discovery of novel biphenomycin congeners by genome mining and establishes biotechnological and biocatalytic tools for the production of biphenomycin analogs. This sets the stage for the structural diversification of the biphenomycin family and thus provides valuable tools for the development of desperately needed, novel antibiotic scaffolds.

## Supporting Information

General methods and additional analytical data are provided in the Supporting Information. The authors have cited additional references within the Supporting Information.

## Conflict of Interests

The authors declare no conflict of interest.

## Supporting information



Supporting Information

## Data Availability

The data that support the findings of this study are available in the Supporting Information of this article.
