# Supplementary material for: Biosynthesis of the Biphenomycin Family of Potent Antibiotics
Source: Angew Chem Int Ed Engl. 2025 Nov 2;64(51):e202516156. doi: 10.1002/anie.202516156 (PMC12707348; doi:10.1002/anie.202516156)
Supplement: Supplementary file 1 — Supporting Information [file ANIE-64-e202516156-s001.pdf]

Biosynthesis of the Biphenomycin Family of Potent Antibiotics

Elisabeth Strunk,[a] Alfred Lobert,[b] Tatiana Khorovich,[a,b] Katia M. Guzman Lucio,[a,b] René Richarz,[c] Maximilian Hohmann,[a] Paul M. D'Agostino[a,b] Tobias A. M. Gulder[a-c*]

[a] E. Strunk, T. Khorovich, K. M. Guzman Lucio, Dr. M. Hohmann, Dr. P. M. D'Agostino, Prof. Dr. T.A.M. Gulder
Chair of Technical Biochemistry, Department of Chemistry and Food Chemistry, Technical University of Dresden, Bergstraße 66, 01069 Dresden, Germany.

[a] A. Lobert, T. Khorovich, K. M. Guzman Lucio, Dr. P. M. D'Agostino, Prof. Dr. T.A.M. Gulder
Department of Natural Product Biotechnology, Helmholtz Institute for Pharmaceutical Research Saarland (HIPS), Helmholtz Centre for Infection Research (HZI) and Department of Pharmacy at Saarland University, PharmaScienceHub (PSH), Campus E8.1, 66123 Saarbrücken, Germany.
E-mail: tobias.gulder@helmholtz-hips.de

[c] Dr. R. Richarz, Prof. Dr. T.A.M. Gulder
Biosystems Chemistry, Faculty of Chemistry, Technical University of Munich, Lichtenbergstraße 4, 85748 Garching, Germany.

Supporting Information

1 General Methods 2
1.1 Chemical and Analytical Methods 2
1.2 Biochemical Methods 3
1.2.1 Cultivation and Extraction of the WT Organisms 3
1.2.2 Transformation, Expression and Purification of Proteins 3
1.2.3 Enzymatic Assay Conditions and Downstream Processing 6
2 Supplementary Figures and Tables 9
3 References 38

1 General Methods

1.1 Chemical and Analytical Methods

Solvents and Chemicals

All chemicals and solvents were purchased of the highest available quality from from commercial suppliers, including Carbolution, Carl Roth, Merck, Selleckchem, Sigma Aldrich, Thermo Fisher Scientific, and VWR. The synthesized protein standards were ordered from Shanghai Royobiotech Co. Ltd in ≥95% purity. Water used for HPLC and LC-MS/MS analyses was prepared using a TKA GenPure water system. Acetonitrile was purchased in LC-MS grade quality from Fisher Scientific and VWR.

LC-HRMS/MS

LC-HRMS/MS analyses were performed using a Bruker Impact II Q-TOF mass spectrometer with ESI source equipped with an UHPLC consisting of an Elute autosampler and a HPG 1300 pump. The system was operated by Bruker Compass® HyStar software and analysis was conducted using Bruker Compass® DataAnalysis software. MS/MS fragmentation pattern analysis was conducted using Bruker BioTools and the Interactive Peptide Spectra Annotator (IPSA) for visual representation.[57]

The following general conditions were used for reversed-phase HPLC chromatography: solvents A = H_2O + 0.05% formic acid (FA); B = ACN + 0.05% FA; gradient: 0–2 min: 5% B, 2–25 min: 5–95% B, 25–28 min: 95% B, 28–30 min: 5% B; flowrate: 0.3 mL/min; column: ACE Ultracore 2.5 Super C18, 150 X 2.1 mm, 2.5 µm; temperature controlled in a column oven (40 °C).

HPLC conditions for determination of B12 content: solvents A = H_2O + 0.05% FA, B = ACN + 0.05% FA; separation method: 0–0.80 min: 5% A, 0.80–1.60 min: 5–20% B, 1.60–3.20 min: 20–41% B, 3.20–3.80 min: 41–5% B, 3.80–6.00 min: 5% B; flowrate: 0.3 mL/min; column: ACE Ultracore 2.5 Super C18, 150 X 2.1 mm, 2.5 µm (40 °C).

Conditions for HPLC on HILIC: solvents A = H_2O + 0.05% FA, B = ACN + 0.05% FA; separation method: 0–2 min: 5% A, 2–12 min: 5–60% A, 12–15 min: 60% A, 15–15.10 min: 60–5% A, 15.10–25 min: 5% A; flowrate: 0.3 mL/min; column: Atlantis Premier BEH Z-HILIC column, 2.1 mm X 100 mm, 1.7 µm (40 °C).

1.2 Biochemical Methods

1.2.1 Cultivation and Extraction of the WT Organisms

For *P. simplex_D*, pre-cultures were prepared by streaking cells on LB agar plates. A single colony was used to inoculate a pre-culture in LB medium, which was then used at a 1:100 ratio to inoculate the main culture in M9 minimal medium. Cultures were grown for 7 days at 30 °C and 180 rpm.

For *S. griseorubigenosus*, spores were prepared by streaking cells on GYM agar plates. After sporulation, a pre-culture of 10 mL seed medium (1% potato starch, 1% glycerol, 0.5% glucose, 1% cotton seed flour, 0.5% yeast extract, 0.5% corn steep liquor, 0.2% calcium carbonate) was prepared and grown over 3 days at 30 °C 180 rpm. The pre-culture was used to inoculate 1 L of SYM (5% sucrose, 0.5% yeast extract, 0.5% ammonium sulfate, 0.1% dikaliumhydrogenphosphate, 0.1 magnesium sulfate, 0.1% sodium chloride, 0.5% calcium carbonate, 0.001% iron sulfate) medium (1:100). Cultures were incubated for 14 days at 30 °C and 200 rpm.

After cultivation, cultures were centrifuged at 6000 rpm for 15 min at 4 °C to remove cells and the supernatant was incubated with a polymeric adsorbent under shaking conditions for approximately 48 h at 4 °C to allow compound binding. Bound material was eluted with methanol and the methanolic extract analyzed by LC-HRMS/MS.

1.2.2 Transformation, Expression and Purification of Proteins

Transformation into Chemically Competent Cells

Chemically competent *E. coli* BL21 (DE3) cells were transformed with the constructed expression plasmids (see Table S1 below) by heat shock at 42 °C for 30 s, followed by incubation on ice for 2 min. For single-expression constructs, one plasmid was added prior to heat shock. For co-expression, multiple compatible plasmids were added simultaneously. After transformation, cells were regenerated in LB medium at 37 °C for 1–2 h, then plated on LB agar plates supplemented with the appropriate antibiotics. Plates were incubated overnight at 37 °C.

Table S1. Expression constructs generated and used in this study.

Empty Plasmid	Gene Insert	Fusion Protein
pMAL-c5x	Sg_*bipC*	MBP-Sg_BipC
pMAL-c6t	Ps_*bipC*	His_6-MBP-Ps_BipC
pMAL-c5x	Sg_*bipD*	MBP-Sg_BipD
pMAL-c6t	Ps_*bipD*	His_6-MBP-Ps_BipD
pHis_8-TEV	Sg_*bipD*	His_8-Sg_BipD
pHis_8-TEV	Ps_*bipD*	His_8-Ps_BipD
pACYC_Duet	Sg_*bipE* (MCS1) Sg_*bipE* (MCS2)	His_8-Sg_BipEF
pACYC_Duet + MBP cassette	Sg_*bipE* (MCS1) Sg_*bipE* (MCS2)	His_6-MBP-Sg_BipEF
pACYC_Duet + MBP cassette	Ps_*bipE* (MCS1) PS_*bipE* (MCS2)	His_6-MBP-Ps_BipEF
pMAL-c5x	Sg_*bipG*	MBP-Sg_BipG
pMAL-c6t	Ps_*bipG*	His_6-MBP-Ps_BipG
pMAL-c5x	Sg_*bipI*	MBP-Sg_BipI
pMAL-c5x	Sg_*bipI*	MBP-Sg_BipI
pHis_8-TEV	Ps_*bipA1*	His_8-Ps_BipA1
pACYC_Duet	Ps_*bipEFC*	Ps_BipEFC
pACYC_Duet	Ps_*bipDEFC*	Ps_BipDEFC

General Conditions for Expression of Fusion Proteins

Single colonies were picked from the transformation plates to inoculate pre-cultures in LB medium with appropriate selection antibiotic at 37 °C and 180 rpm overnight. Main cultures in TB medium were inoculated 1:100 with the preculture and corresponding antibiotic. The cultures were grown to OD_{600} of 0.8-1.0 and cooled down to 4 °C before induction with 0.1 mM IPTG and addition of further supplements (see below). Expression cultures were grown for 16-18 hours at 16 °C before harvesting the cells by centrifugation at 6000 rpm for 10 minutes at 4 °C. The cell pellets were washed with 0.9 % NaCl solution and frozen at – 80 °C until further purification.

The following adjustments were made to the general protocol for production of:

- **BipD:** coexpression with helper plasmid pBAD1030K/S::*btuCDEFB;* induction at OD_{600} of 0.3; supplementation with 0.5 g/L L-arabinose, 5 mg/L CN-Cob, 0.1 mM ammonium iron(II) sulfate, and 0.1 mM cysteine.
- **BipEF and BipG:** supplementation with 0.1 mM ammonium iron(II) sulfate upon induction.
- **BipI:** supplementation with 0.1 mM $ZnCl_2$ upon induction.
- **BipC:** supplementation with 0.1 mM $MnCl_2$ upon induction.

Purification of Fusion Protein

Pellets containing MBP-tagged proteins were thawed and 4 mL/g ÄKTA running buffer (50 mM Tris, 200 mM NaCl, pH 7.8) was added. Pellet were resuspended and lysed by ultrasonication

on ice (38 % amplitude, 5 sec on / 10 sec off over 2 min). The resulting supernatants were separated from the cells by centrifugation at 12000 rpm over 30 min at 4 °C. Supernatant were filtered and subjected onto a 5 mL MBP-Trap pre-quilibrated with ÄKTA running buffer at a flow rate of 5 mL/min. After application of the supernatant, unbound protein fraction was washed off with ÄKTA running buffer. The bound protein was eluted with ÄKTA elution buffer (50 mM Tris, 200 mM NaCl, 10 mM maltose, pH 7.8).

Nickel-NTA Affinity Purification

Cell pellets were resuspended in lysis buffer A (20 mM NaH_2PO_4, 500 mM NaCl, 20 mM imidazole, pH 7.6) or lysis buffer B (50 mM HEPES, 300 mM NaCl, 10 mM imidazole, 2 mM β-mercaptoethanol, pH 8.0) at 4 mL/g cell pellet. Cells were lysed on ice using a sonicator (5s on/ 10s off, 2 min, 38% amplitude). Lysates were clarified by centrifugation at 12000 rpm for 30 min at 4°C and the supernatant was collected. Ni-NTA resin (1 mL per liter of original culture) was added to the supernatant and incubated for 2 h on ice. The resin was washed with 10–20 column volumes (CV) of lysis buffer. Proteins were eluted with 5 CV of elution buffer (250 mM imidazole in lysis buffer).

Buffer Exchange and Protein Concentration Determination

Proteins were transferred into storage buffer (Table S3) using PD-10 desalting columns pre-equilibrated with storage buffer. Protein concentration were increased using Amicon Ultra centrifugal filters (MWCO as appropriate). Protein concentration was determined by UV-Vis spectrophotometry using the method of Whitaker and Granum,[58] except for BipD, which was quantified using the Bradford assay.

Table S2. Amicon Ultracentrifugation vessels that were used during concentration.

Protein	Amicon MWCO (kDa)
BipA	3
BipC	30
MBP-BipD	100
His-BipD	30
MBP-BipEF/His-BipEF	10
BipG	30
BipI	30

1.2.3 Enzymatic Assay Conditions and Downstream Processing

Enzymatic Assay Conditions

In vitro assays were performed using a substrate concentration of 1 mg/mL. All assay components were prepared in the respective assay buffer (see Table S3 below), with any required supplements added prior to enzyme addition. Enzymes were added at 20 µM (except for BipI: 50 µM) as the final component to initiate the reaction. For multi-step conversions, reactions were carried out in a stepwise manner, with downstream enzymes added sequentially. Specific conditions for each enzyme are detailed in Table S2. All assays were incubated at 25 °C over night before quenching by heat inactivation at 80 °C for 5 min, followed by centrifugation at 13000 rpm for 30 min at 4 °C to remove precipitated protein. Supernatants were either analyzed directly by LC-HRMS/MS, subjected to GluC digestion and LC-HRMS/MS analysis, or used in further enzymatic conversions with downstream enzymes.

The following adjustments were made for the individual enzyme reactions:

- **BipC:** assays supplemented with 1 mM $MnCl_2$ and 5 mM DTT.
- **BipD:** BipD was reconstituted prior to storage buffer exchange with 10 mM DTT, 1 mM sodium sulfide, 200 µM hydroxocobalamin, and 1.1 mM ammonium iron(II) sulphate over night at 4 °C. For the assays, 10 mM DTT and 1 mM SAM as well as a reducing system (15 mM Ti(III)citrate; 20 µM fdx, 5 µM fdr, 2 mM NADPH; 1 mM methyl viologen (MV), 2 mM NADPH; 1 mM DTH) were added. All assays were carried out under anaerobic conditions.
- **BipEF:** assays supplemented with 1 mM ammonium iron(II) sulphate and 5 mM DTT for dihydroxylation activity. Without supplementation for proteolytic activity.
- **BipG:** anaerobic reconstitution with a threefold excess of ammonium iron(II) sulphate before application in the assay. Assays supplemented with 0.8 µM α-KG and additional 20 µM ammonium iron(II) sulphate.
- **BipI:** assays supplemented with 0.1 mM $MnCl_2$.

Table S3. Buffers used during purification, storage, and assay for each enzyme.

Protein	Lysis Buffer	Storage Buffer	Assay Buffer
MBP-BipC	ÄKTA running buffer	20 mM Tris, 100 mM NaCl, 5 mM DTT, 1 mM NaCl pH 7.6	50 mM Tris pH 8.0
MBP-BipD	ÄKTA running buffer	50 mM Tris, 10 mM DTT pH 8.0	50 mM Tris, pH 8.0
His-BipD	B	50 mM Tris, 10 mM DTT pH 8.0	50 mM Tris pH 8.0
MBP-BipEF	ÄKTA running buffer	25 mM HEPES, 300 mM NaCl pH 7.5	50 mM Tris pH 8.0
His-BipEF	A	20 mM Tris, 100 mM NaCl pH 7.6	50 mM Tris pH 8.0
MBP-BipG	ÄKTA running buffer	20 mM MOPS, 300 mM NaCl, 3 eq. Fe^{2+} pH 7.5	100 mM Tris pH 7.5
MBP-BipI	ÄKTA running buffer	125 mM NaCl, 50 mM Tris pH 7.5	125 mM NaCl, 50 mM Tris pH 7.5

Protein Digestion Using GluC

Endoproteinase GluC digestions were performed on quenched enzymatic assays using a substrate-to-protease ratio of approximately 80:1. Reactions were incubated at 37 °C for 16–18 hours.

***In vivo* Co-Expression**

Co-expression of pHis8::*Ps_bipA* and either pACYC-Duet::*Ps_bipEFC* or pACYC-Duet::*Ps_bipDEFC* were performed in *E. coli* BL21 (DE3). For constructs with *Ps_bipD*, the helper plasmid pBAD1030S::*btuCDEFB* was additionally transformed into the host strain. Chemically competent cells were transformed by heat shock and plated on LB agar plates supplemented with the corresponding antibiotics. Plates were incubated overnight at 37 °C prior to expression. A single colony from LB agar plates was used to inoculate a pre-culture, which was grown overnight at 37 °C in LB medium containing the appropriate antibiotics. The following day, the pre-culture was used to inoculate Terrific Broth (TB) medium (1:100 dilution) supplemented with antibiotics. Cultures were grown at 37 °C to an OD_{600} of ~0.8, then cooled on ice for ≥30 min. Expression was induced with 10 mg/L cyanocobalamin and 0.5 g/L L-arabinose (for co-expression with the helper plasmid), 0.5 mM IPTG, 1 mM ammonium iron(II) sulphate, 0.1 mM $MnCl_2$, 0.1 mM cysteine, and 0.1 mM methionine. Cultures were incubated at 16 °C and 120 rpm for 72 h.

Purification of *in vivo* Modified Ps_BipA

Cells were harvested by centrifugation at 6000 rpm at 4 °C for 15 min and washed with 0.9% NaCl solution prior to storage at –80 °C. Lysis was performed in two steps. First, the frozen cell pellets were resuspended in lysis buffer B (20 mM NaH2PO4, 500 mM NaCl, 20 mM imidazole, pH 7.6) and disrupted by sonication (4 min, 10s on/off, 38% amplitude) on ice. After centrifugation at 12000 rpm for 30 min at 4 °C, the supernatants were collected. The remaining pellet was subsequently treated for inclusion body solubilization using denaturing lysis buffer (6 M guanidine hydrochloride, 0.3 M NaCl, 50 mM Tris-HCl, 10% glycerol, pH 8.0) and the resuspended pellet sonicated on ice (2 min, 10s on/off, 38% amplitude). The resulting slurries was centrifuged and the supernatants collected for further analysis. Supernatants from both lysis steps were combined and Ni-NTA resin (1 mL per L of original culture) was added. The mixture was incubated for 2 h at 4 °C with gentle shaking. After incubation, the resin was transferred to a gravity-flow column and washed with 50 CV of lysis buffer B. Bound protein was eluted using 10 CV elution buffer (20 mM NaH2PO4, 500 mM NaCl, 250 mM imidazole, pH 7.6). Eluted fractions were buffer-exchanged to 50 mM Tris-HCl pH 8.0 and concentrated using Amicon® centrifugal concentrators. Samples were lyophilized and subsequently re-dissolved in the same buffer. Dissolved protein was GluC digested and analyzed by LC-HRMS/MS.

Determination of Cobalamin Species by LC-MS/MS

To identify the bound cobalamin species (methyl-, adenosyl-, or hydroxocobalamin), an aliquot of the purified enzyme prior to cofactor reconstitution was treated with 100 mM H_2SO_4 under exclusion of light to minimize photodegradation. The protein was denatured by acidification and the sample was subsequently centrifuged at 12000 rpm for 10 min to pellet precipitated protein. The resulting supernatant, containing the released cobalamin species, was immediately analyzed by LC-MS/MS. Chromatographic separation (for conditions, see above) and detection were carried out using an equally treated standard mix of methylcobalamin, adenosylcobalamin, and hydroxocobalamin for retention time and fragmentation pattern comparison.

2 Supplementary Figures and Tables

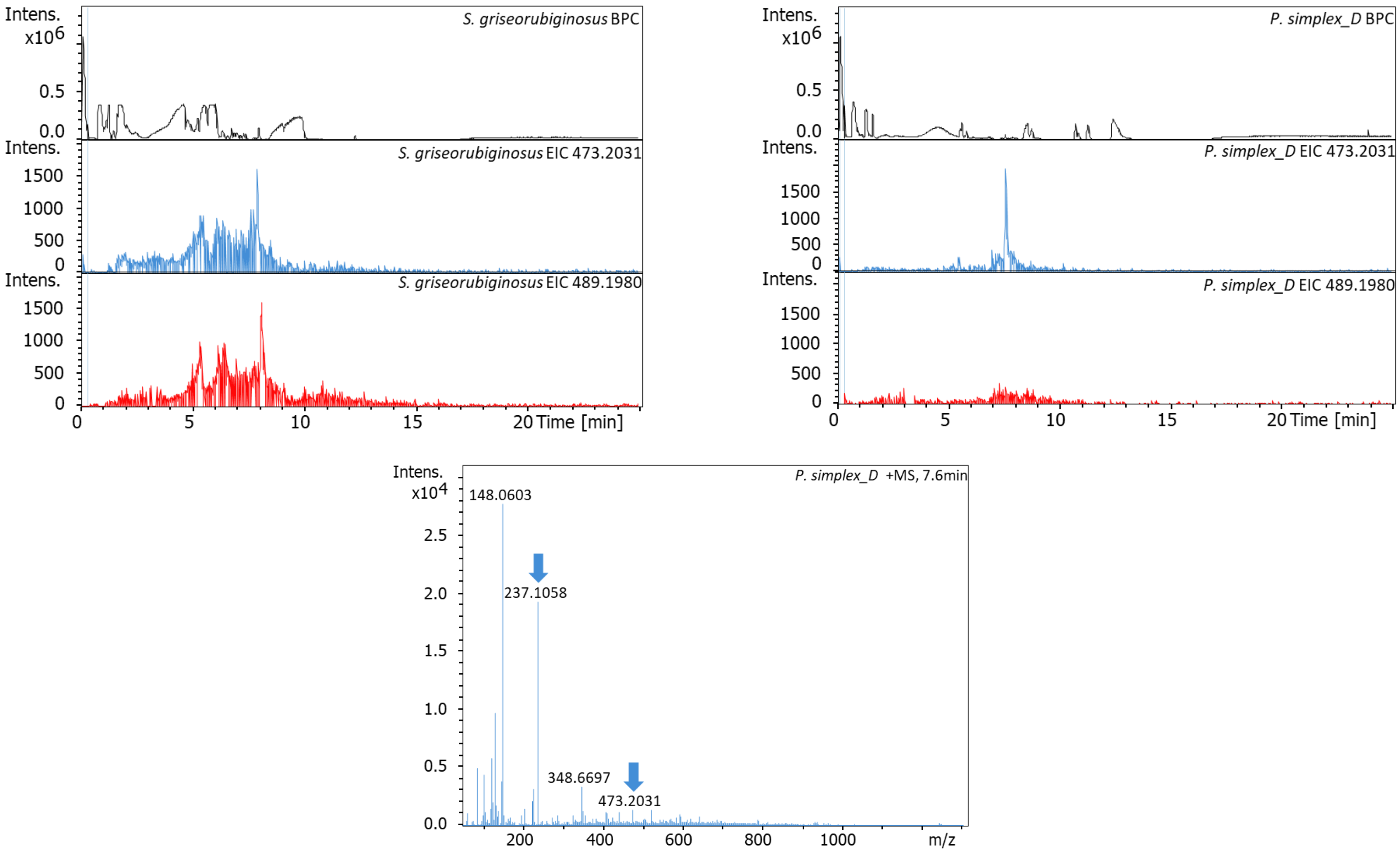

Figure S1. LC-HRMS of extracts from WT fermentation of *S. griseorubiginosus* (left) and *Peribacillus simplex_D* (right). Shown is the BPC, along with the EICs for biphenomycin A (red) *and biphenomycin B (blue). The mass spectrum of biphenomycin B with an *m/z* of 473.2031 $[M+H]^+$ and 237.1058 $[M+2H]^{2+}$ is exemplarily shown (bottom).

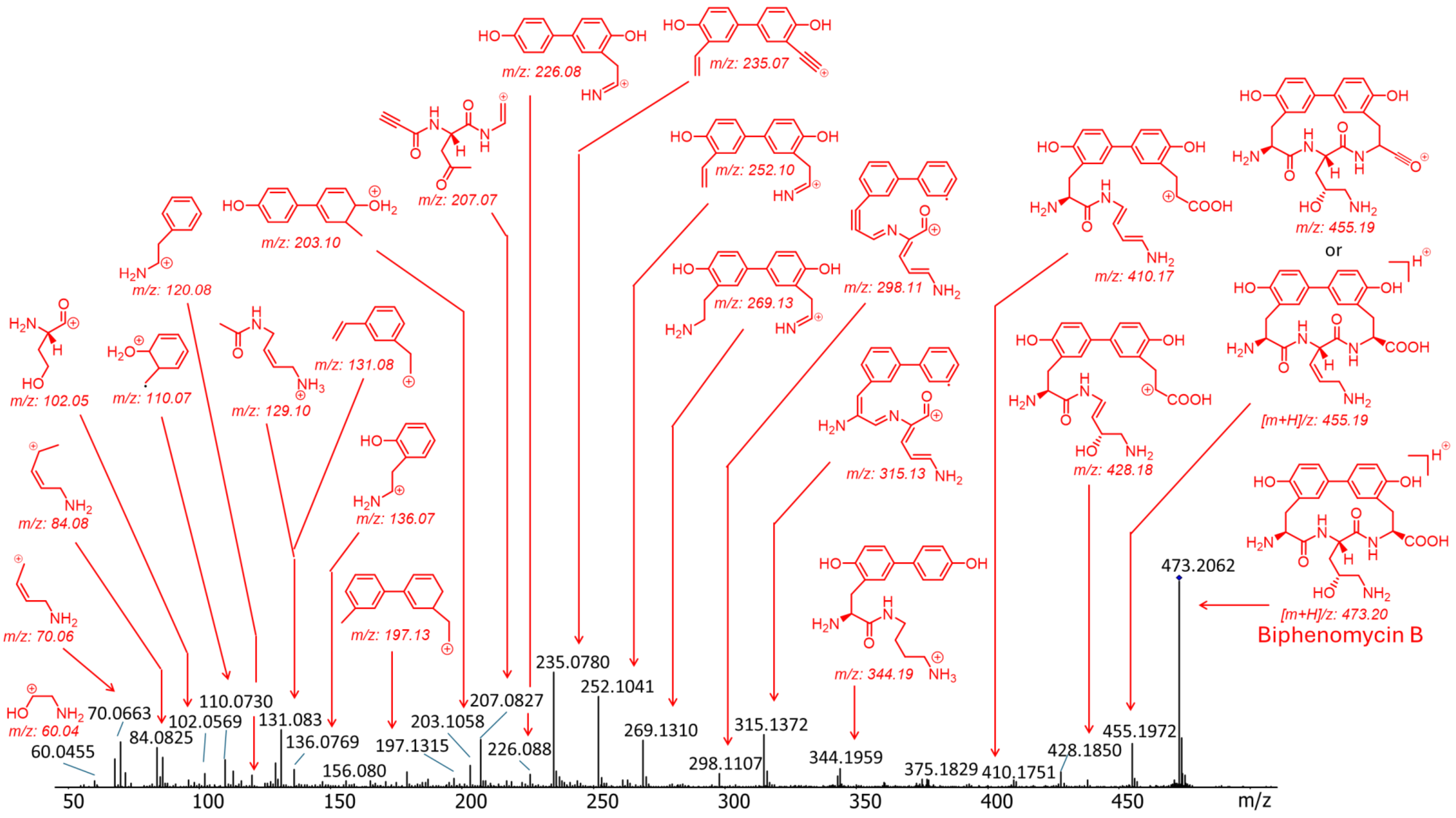

Figure S2. Analysis of HRMS/MS fragmentation pattern of biphenomycin B.

Region	Type	From	To	Similarity Confidence	Most similar known cluster	
Region 1	aminopolycarboxylic-acid	276,938	290,357	High	Ethylenediaminesuccinic acid hydroxyarginine	other:other
Region 2	T1PKS	505,623	549,855			
Region 3	terpene-precursor	644,351	665,325			
Region 4	melanin	665,427	675,771	Medium	melanin	other:other
Region 5	CDPS	694,462	715,349	Low	9-methylstreptimidone	PKS:Type I
Region 6	terpene	855,715	876,653			
Region 7	T2PKS	1,243,981	1,316,442	Medium	julichrome Q3-3/julichrome Q3-5	PKS
Region 8	terpene	1,818,600	1,839,610			
Region 9	NAPAA	1,895,769	1,929,632	High	ε-Poly-L-lysine	NRPS:Type I
Region 10	ectoine	2,170,615	2,181,031	High	ectoine	other:other
Region 11	melanin	3,314,666	3,325,151			
Region 12	NI-siderophore	3,426,595	3,456,367	High	desferrioxamin B/desferrioxamine E	other:other
Region 13	T2PKS	4,802,065	4,874,580	High	spore pigment	PKS
Region 14	terpene	6,001,805	6,022,728	High	albaflavenone	terpene
Region 15	terpene-precursor	6,036,453	6,057,712			
Region 16	NRPS	6,307,803	6,352,059	Medium	SF2768	NRPS:Type I
Region 17	lassopeptide , NI-siderophore	6,731,106	6,781,540	High	MS-271	ribosomal:RiPP
Region 18	RiPP-like	7,002,928	7,014,259			
Region 19	butyrolactone , terpene	7,074,490	7,099,856	High	γ-butyrolactone	other:other
Region 20	T1PKS , furan	7,136,357	7,181,183	Low	methylenomycin A	other:other
Region 21	hydrogen-cyanide	7,200,139	7,213,861			
Region 22	NI-siderophore	7,303,185	7,334,336			
Region 23	terpene	7,750,584	7,777,222	High	hopene	terpene
Region 24	RiPP-like	8,119,079	8,130,017			
Region 25	RiPP-like , lanthipeptide-class-iii	8,275,074	8,303,027	High	informatipeptin	ribosomal:RiPP:Lanthipeptide
Region 26	terpene	8,458,555	8,484,745	High	isorenieratene	terpene
Region 27	T1PKS	8,678,412	8,748,217	Low	akaeolide	PKS
Region 28	T3PKS , indole	9,177,319	9,227,811	High	germicidin	other:other

Region	Type	From	To	Similarity Confidence	Most similar known cluster	
Region 12.1	transAT-PKS	15,020	41,887	Low	inthomycin B	PKS
Region 24.1	T1PKS	1	10,474	Low	K-41A	PKS
Region 27.1	NRPS	1	31,582			
Region 30.1	T3PKS	1	33,544	High	germicidin	other:other
Region 42.1	terpene-precursor	11,963	33,093			
Region 42.2	terpene	35,353	56,279	High	albaflavenone	terpene
Region 63.1	NRPS , azoxy-crosslink	9,955	53,080	Low	Azodyrecin A/Azodyrecin B/Azodyrecin C	other:other
Region 75.1	lanthipeptide-class-ii	1	18,282	Medium	birimositide	ribosomal:RiPP:Lanthipeptide
Region 84.1	RiPP-like	6,428	16,643	Low	informatipeptin	ribosomal:RiPP:Lanthipeptide
Region 90.1	RiPP-like	1	5,543			
Region 104.1	ectoine	1	6,286	High	ectoine	other:other
Region 114.1	T3PKS	1	33,295	High	flaviolin/1,3,6,8-tetrahydroxynaphthalene	PKS
Region 132.1	terpene	52,420	67,801	High	geosmin	terpene:Sesquiterpene
Region 136.1	NI-siderophore	11,905	40,844			
Region 138.1	NI-siderophore	5,286	31,608	High	desferrioxamine	other:other
Region 139.1	terpene	1	6,240	Low	isorenieratene	terpene
Region 145.1	melanin , terpene-precursor	36,443	58,727	Medium	melanin	other:other
Region 174.1	T1PKS , PKS-like	1	39,730			
Region 183.1	T2PKS , RiPP-like	8,021	80,532	High	spore pigment	PKS
Region 190.1	redox-cofactor	92,819	114,975			
Region 197.1	NRPS-like , NRPS	97,513	156,789	Medium	JBIR-34/JBIR-35	NRPS:Type I
Region 198.1	triceptide , lanthipeptide-class-i	22,114	50,078			
Region 217.1	terpene	1	18,839	Medium	hopene	terpene
Region 221.1	RiPP-like	9,135	20,073			
Region 224.1	lassopeptide	35,502	50,559			
Region 226.1	other	3,707	31,905			
Region 233.1	NI-siderophore	42,928	72,904	Low	kinamycin	PKS
Region 258.1	PKS-like , butyrolactone	1	24,437			
Region 262.1	lanthipeptide-class-iii	1	16,046	Low	informatipeptin	ribosomal:RiPP:Lanthipeptide
Region 263.1	melanin	43,837	54,325			
Region 264.1	NRPS , NAPAA	6,337	33,996			
Region 289.1	transAT-PKS-like , NRPS , NRPS-like	1	26,131	Low	inthomycin B	PKS
Region 291.1	T1PKS	1	34,408	High	1-heptadecene	PKS:Type I

Figure S3. Bioinformatic analyses of the genomes of *S. griseorubiginosus* (left) and *S. filipinensis* (right) using antiSMASH (8.0.1)

Table S4. BLAST homology search of genes in the *bip* BGC in *S. griseorubigenosus*.

Gene	Size	Predicted Gene Function	Closest homolog (organism), accession	Identity
Sg_bipA	126 bp	Precursor Peptide	Multispecies_ hypothetical protein (Streptomyces) WP_267884975.1	100.00%
Sg_bipB	1401 bp	MFS transporter	MFS transporter [Streptomyces griseorubiginosus] WP_162951972.1	100.00%
Sg_bipC	1245 bp	UPF0489 arginase	tetratricopeptide repeat protein [Streptomyces griseorubiginosus] WP_120050100.1	100.00%
Sg_bipD	1923 bp	B12-containing rSAM	B12-binding domain-containing radical SAM protein [Streptomyces griseorubiginosus] WP_388203055.1	99.84%
Sg_bipE	939 bp	MNIO	multinuclear nonheme iron-dependent oxidase [Streptomyces griseorubiginosus] WP_388423685.1	100.00%
Sg_bipF	768 bp	MNIO partner	hypothetical protein [Streptomyces griseorubiginosus] WP_388423688.1	99.61%
Sg_bipG	816 bp	γ-Orn-hydroxylase	JmjC domain-containing protein [Streptomyces griseorubiginosus] WP_062020578.1	100.00%
Sg_bipH	1050 bp	aKG-HExxH beta-hydroxylase	aKG-HExxH-type peptide beta-hydroxylase [Streptomyces griseorubiginosus] WP_388423691.1	99.43%
Sg_bipI	1041 bp	Metalloprtoease	metallopeptidase TldD-related protein [Streptomyces griseorubiginosus] WP_366635979.1	97.41%

Table S5. BLAST homology search of genes in the *bip* BGC in *P. simplex_D*

Gene	Size	Predicted Gene Function	Closest homolog (organism), accession	Identity
Ps_bipA1	135bp	Precursor Peptide	MULTISPECIES: hypothetical protein [Peribacillus] WP_260286708.1	100.00%
Ps_bipA2	135bp	Precursor Peptide	MULTISPECIES: hypothetical protein [Peribacillus] WP_260286709.1	100.00%
Ps_bipA3	135bp	Precursor Peptide	MULTISPECIES: hypothetical protein [Peribacillus] WP_260286710.1	100.00%
Ps_bipB	1392bp	MFS transporter	MFS transporter [Peribacillus simplex] WP_387600696.1	100.00%
Ps_bipC	1245 bp	UPF0489 arginase	UPF0489 family protein [Peribacillus simplex] WP_387600706.1	100.00%
Ps_bipD	1887 bp	B12-containing rSAM	MULTISPECIES: B12-binding domain-containing radical SAM protein [Peribacillus] WP_387600700.1	100.00%
Ps_bipE	939 bp	MNIO	MULTISPECIES: multinuclear nonheme iron-dependent oxidase [Peribacillus] WP_260286712.1	100.00%
Ps_bipF	795 bp	MNIO partner	hypothetical protein [Peribacillus simplex] WP_387600703.1	100.00%
Ps_bipG	696 bp	γ-Orn-hydroxylase	cupin-like domain-containing protein [Peribacillus simplex] WP_387600712.1	99.57%
Ps_bipI	1077 bp	Metallo-protease	metallopeptidase TldD-related protein [Peribacillus simplex] WP_387600709.1	100.00%

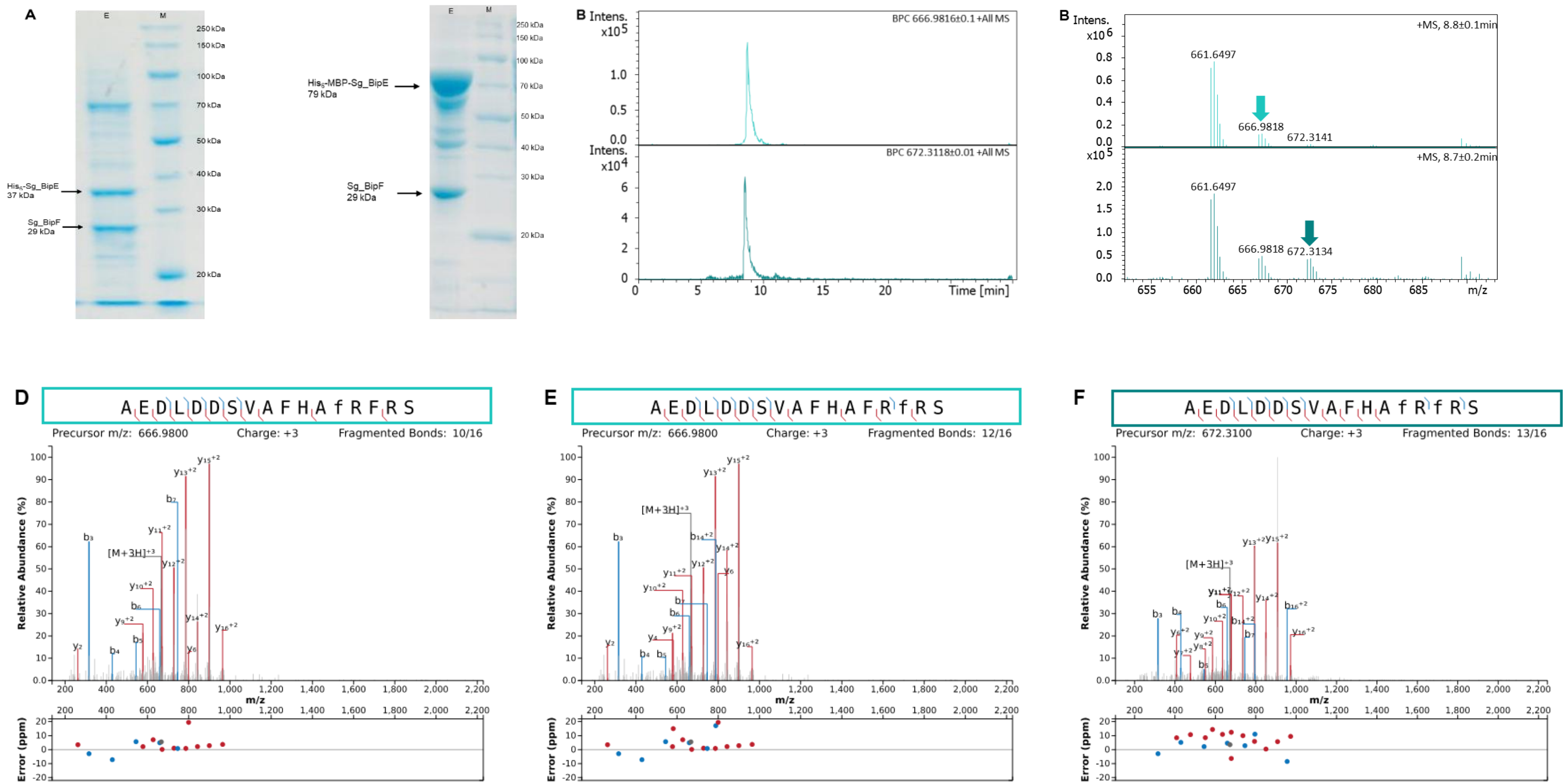

Figure S4. A. SDS-PAGE analysis of the His$_8$-tagged (left) and MBP-tagged (right) versions of Sg_BipEF. The molecular weight marker is shown in both gels (M). The purified His$_8$-Sg_BipEF-showed a distinct band at approximately 37 kDa for untagged BipE and 29 kDa for BipF, while the MBP tagged version appeared at approximately 79 kDa, consistent with the expected sizes of the fusion protein, while untagges BipF remained at 29 kDa. **B.** LC-HRMS/MS analysis of the enzymatic modification of the precursor peptide by Sg_BipEF. The EIC reveals mono- (top) and di-hydroxylation (bottom) of Sg_BipA . **C.** HRMS spectra of the corresponding product peaks confirming the respective mass shifts from *m/z* of 661.6497 to *m/z* 666.9816 $[M+3H]^{3+}$ (mono-hydroxylation, top) and to *m/z* 672.3118 $[M+3H]^{3+}$ (di-hydroxylation, bottom), respectively. **D-E.** MS/MS fragmentation patterns of each modification matched to the expected b- and y-ions for mono- (for the two possible regioisomeric products; **D**, **E**) and di-hydroxylation (**F**).

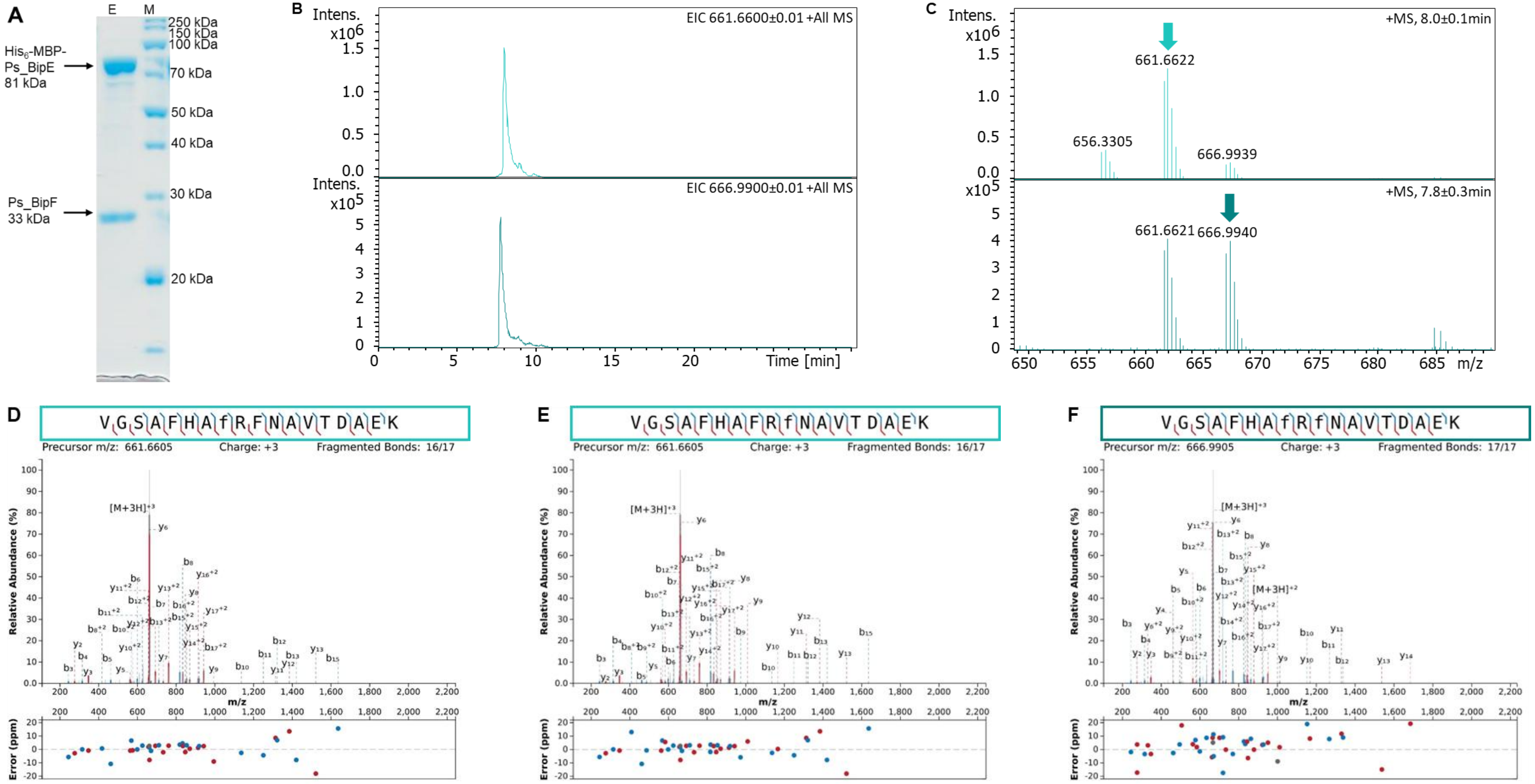

Figure S5. A. SDS-PAGE analysis of the purified heterodimeric enzyme Ps_BipEF containing a His_6-MBP tag. **A.** SDS gel showing the expected band at approx. 81 kDa for His_6-MBP-Ps_BipE, along with a second band for Ps_BipF at approx. 33 kDa . **B.** LC-HRMS/MS analysis of the enzymatic modification of the precursor peptide by Ps_BipEF. The EIC displays the mono- (top) and di-hydroxylation (bottom) of Ps_BipA1. **C.** HRMS spectra of the corresponding product peaks confirming the respective mass shifts from *m/z* of 656.3305 to *m/z* 661.6622 $[M+3H]^{3+}$ (mono-hydroxylation, top) and to *m/z* 666.9940 $[M+3H]^{3+}$ (di-hydroxylation, bottom), respectively. **D-E.** MS/MS fragmentation patterns of each modification matched the expected b- and y-ions for mono- (for the two possible regioisomeric products; **D**, **E**) and di-hydroxylation (**F**).

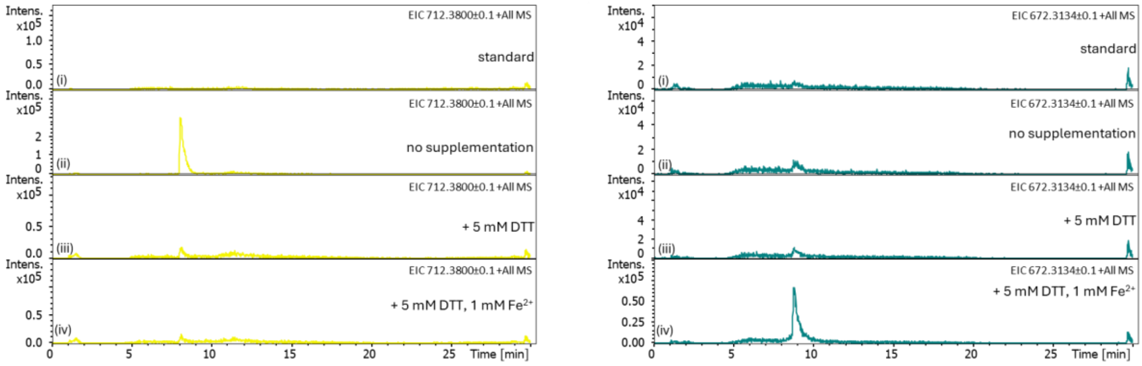

Figure S6. LC-HRMS/MS analysis demonstration the dual activity of Sg_BipEF under varying conditions. Left panel (yellow): Proteolytic cleavage of the *N*-terminal region of the CP, resulting in the appearance of the proteolysis product CP-LP (*m/z* 712.3800, $[M+H]^{+}$). The following chromatograms are shown: (i) Sg_BipA standard; (ii) Sg_BipA + 20 µM Sg_BipEF resulting in proteolysis; (iii) supplementation with 5 mM DTT and (iv) additional 1 mM Fe^{2+}, with both conditions leading the barely detectable amounts of proteolytic product. Right panel (teal): Hydroxylation activity of Sg_BipEF monitored using identical conditions (*m/z* of di-hydroxylated product 672.3118, $[M+3H]^{3+}$), with minute amounts of product without supplementation (ii, iii) and strong hydroxylation activity in the presence of DTT and Fe^{2+} (iv).

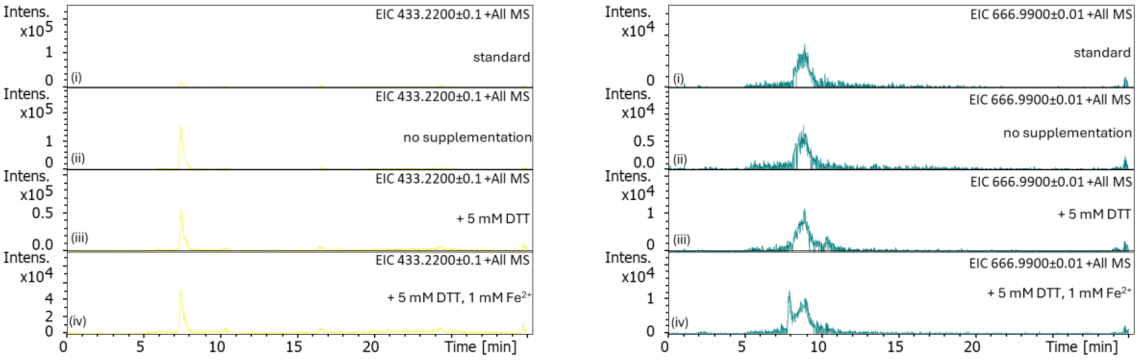

Figure S7. LC-HRMS/MS analysis demonstration the dual activity of Ps_BipEF under varying conditions. Left panel (yellow): Proteolytic cleavage of the *N*-terminal region of the CP, resulting in the appearance of CP-LP fragment (*m/z* 433.22 $[M+3H]^{3+}$). The following chromatogtamms are shown: (i) Ps_BipA1 standard, (ii) Ps_BipA1 + 20 µM Ps_BipEF resulting in proteolysis, (iii) supplementation with 5 mM DTT and (iv) additional 1 mM Fe^{2+}, with both conditions leading the barely detectable amounts of proteolytic product. Right panel (teal): Hydroxylation activity of Ps_BipEF monitored using identical conditions (*m/z* of di-hydroxylated 666.99 $[M+3H]^{3+}$), with minute amounts of product without supplementation (ii, iii) and strong hydroxylation activity in the presence of DTT and Fe^{2+} (iv).

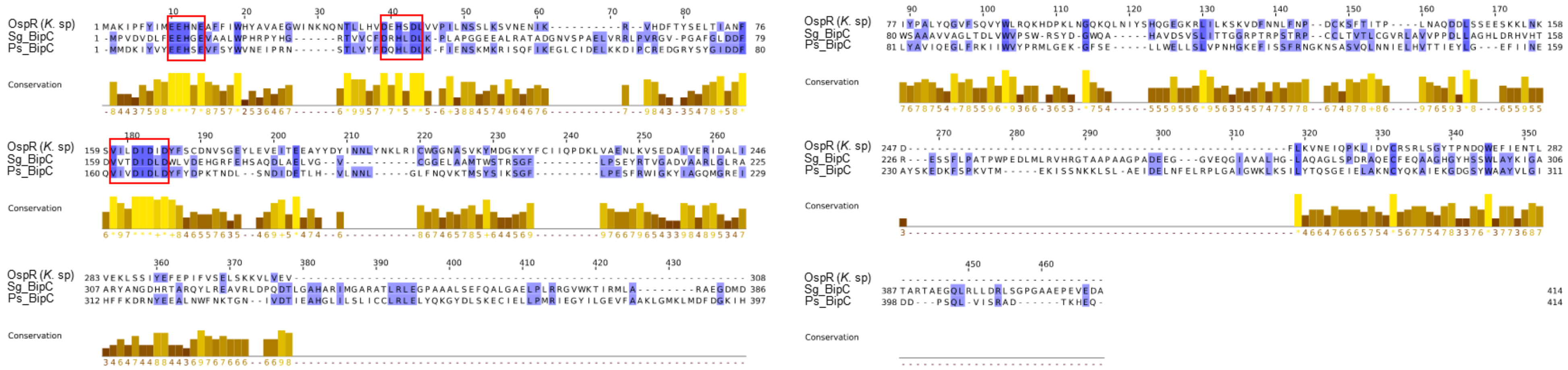

Figure S8. MSA of OspR[3] (*K.* sp.), Sg_BipC, and Ps_BipC higlighting the matching motifs (red boxes) of the AA sequences.

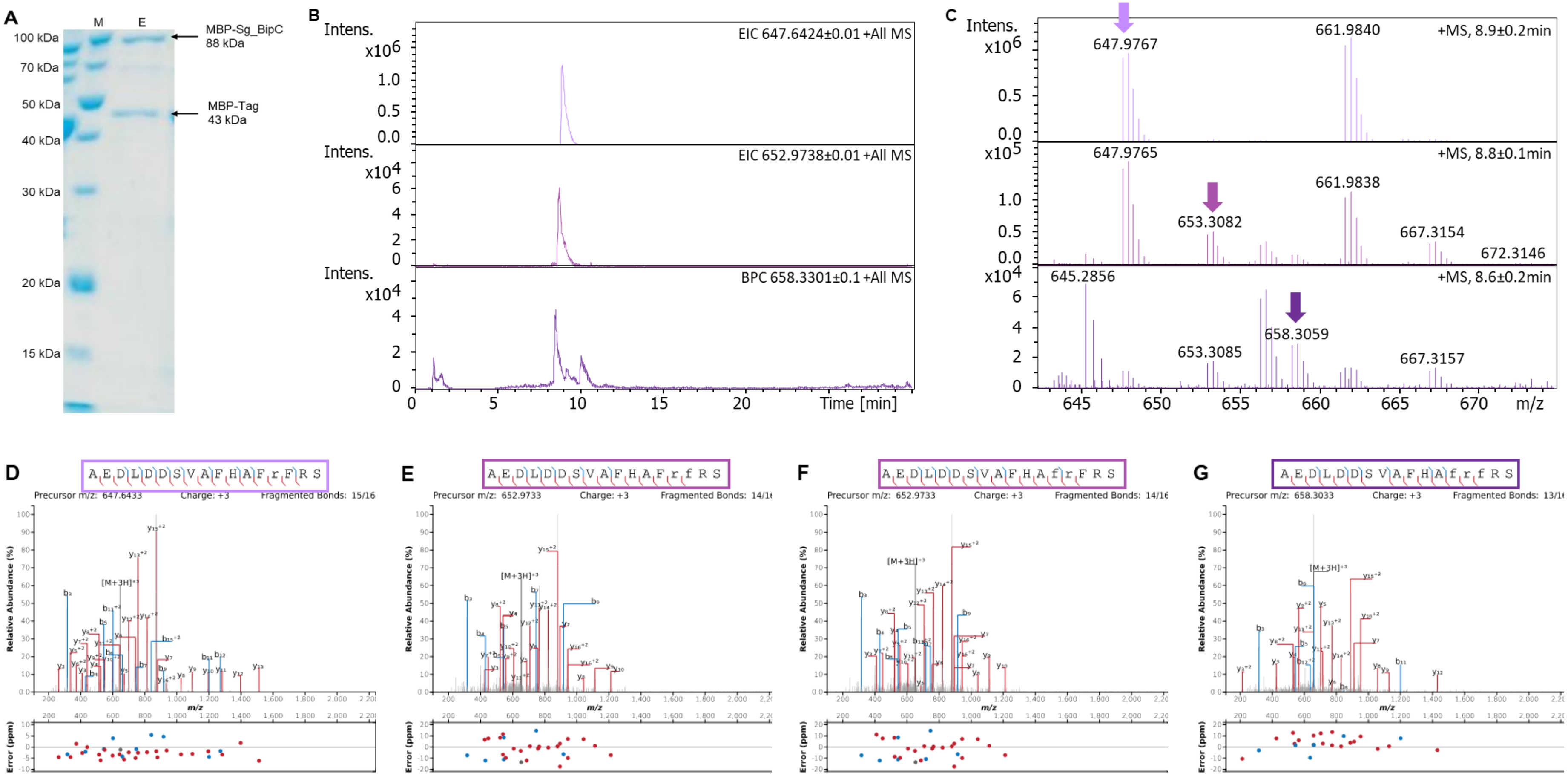

Figure S9. A. SDS-PAGE analysis of the purified protein Sg_BipC. The marker (left lane) is shown alongside the purified protein sample (right lane). The major band corresponds to the expected mass of the fusion protein including the MBP tag (88 kDa). **B.** LC-HRMS/MS analysis of the enzymatic modification of the precursor peptide by Sg_BipC showing EICs corresponding to (top to bottom) the unmodified peptide, the mono-hydroxylated intermediate, and the final di-hydroxylated product (product of Sg_BipEF). **C.** HRMS spectra depicting the corresponding mass shifts with *m/z* 647.64 $[M+3H]^{3+}$ (unmodified), *m/z* 652.97 $[M+3H]^{3+}$ (mono-hydroxylation) and *m/z* 656.33 $[M+3H]^{3+}$ (di-hydroxylation). **D-E.** MS/MS fragmentation patterns matched the expected b- and y-ions for the umodified CP (**D**), mono- (for the two possible regioisomeric products; **E**, **F**) and di-hydroxylated (**G**) product.

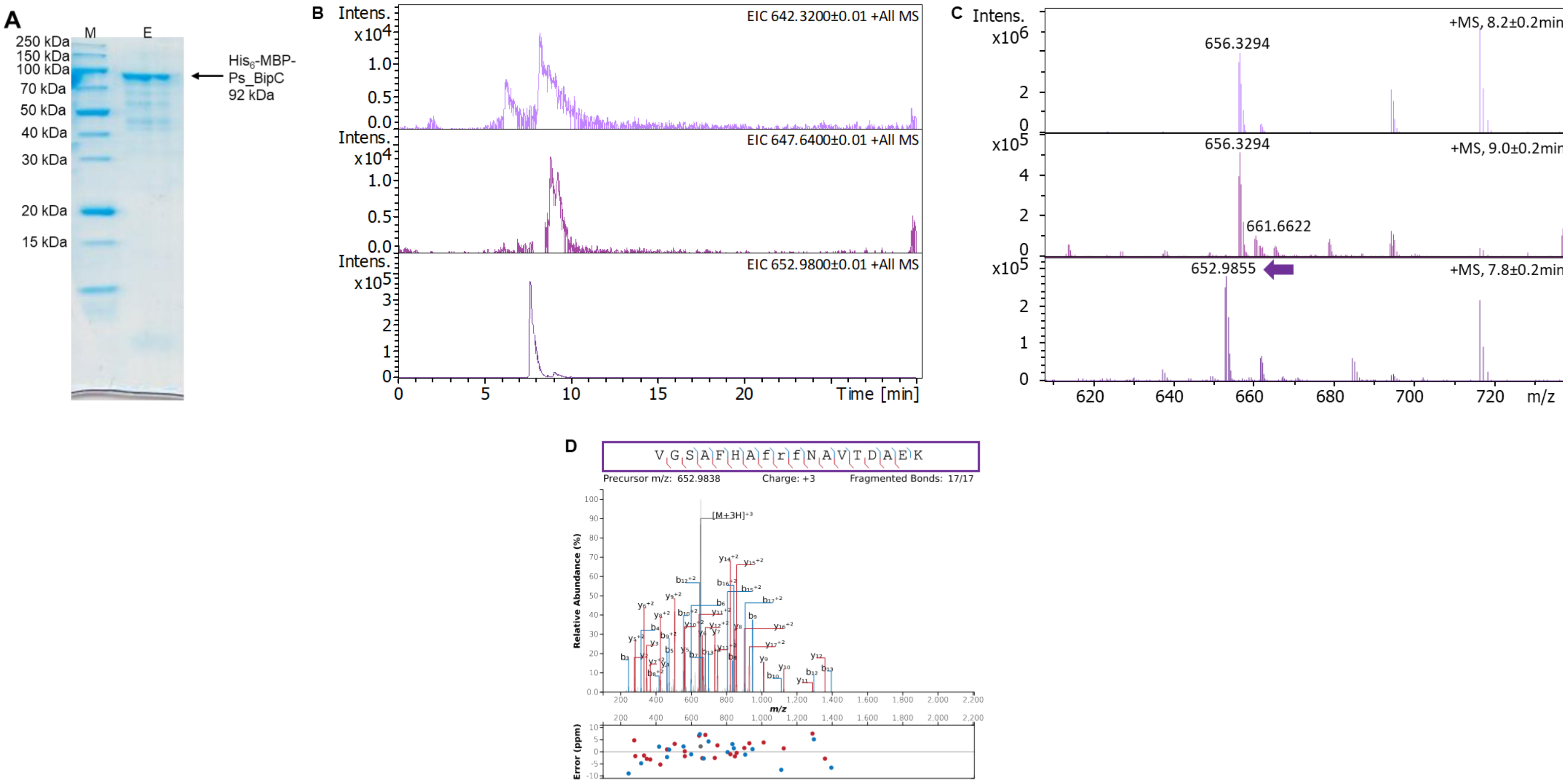

Figure S10. A. SDS-PAGE analysis of the purified His$_6$-MBP-Ps_BipC showed a strong band at the expected molecular weight of ~92 kDa with a marker (M) as reference. **B.** LC-HRMS/MS analysis of the enzymatic modification of the precursor peptide by Ps_BipC. The EICs of the putative deguanidated products resulting from (top to bottom) the unmodified peptide, the mono-, and the di-hydroxylated analog (final product of Ps_BipEF) are shown. **C.** HRMS spectra of the peaks depicted in panel B are shown. Only the di-hydroxylated species (bottom) was transformed into the expected product at *m/z* 652.9855 $[M+3H]^{3+}$; no product formation for the umodified (top) and mono-hydroxylated (middle) was detected, only the assay substrate Ps_BipA1 (*m/z* 656.3294, $[M+3H]^{3+}$) was present. **D.** MS/MS fragmentation patterns of dihydroxylated product matched the expected b- and y-ions.

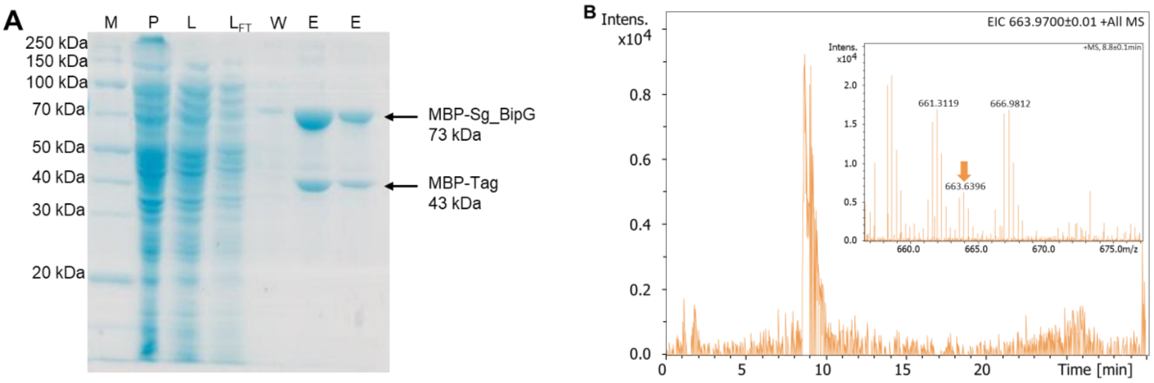

Figure S11. A. SDS-PAGE analysis of the purification process of Sg_BipG. Depicted are marker (M), pellet (P), lysate (L), lysate flow-through (L_{FT}), wash (W), and purified eluted protein sample. **B.** LC-HRMS/MS analysis of the deguanidinated and di-hydroxylated Sg_BipA (previous reactions catalyzed by Sg_BipEF/C) from an enzyme assay with Sg_BipG. The *m/z* of 663.63 corresponds to product with additional hydroxylation ($[M+3H]^{3+}$).

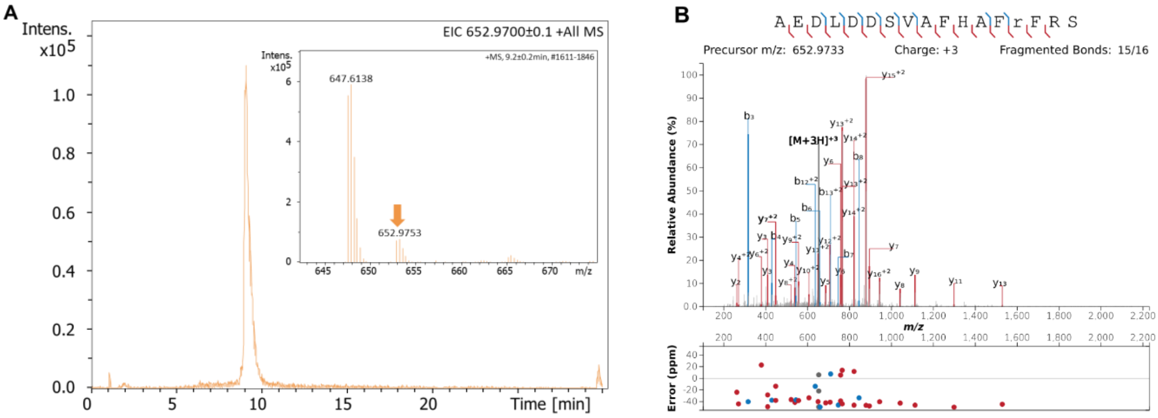

Figure S12. A. LC-HRMS/MS analysis of the deguanidinated Sg_BipA(FOrnF) from an enzyme assay with Sg_BipG. HPLC chromatogram and MS analysis of product peak (*m/z* 652.97, $[M+3H]^{3+}$) with additional hydroxylation. **B.** MS/MS data matched to the calculated fragment pattern of the hydroxylated product.

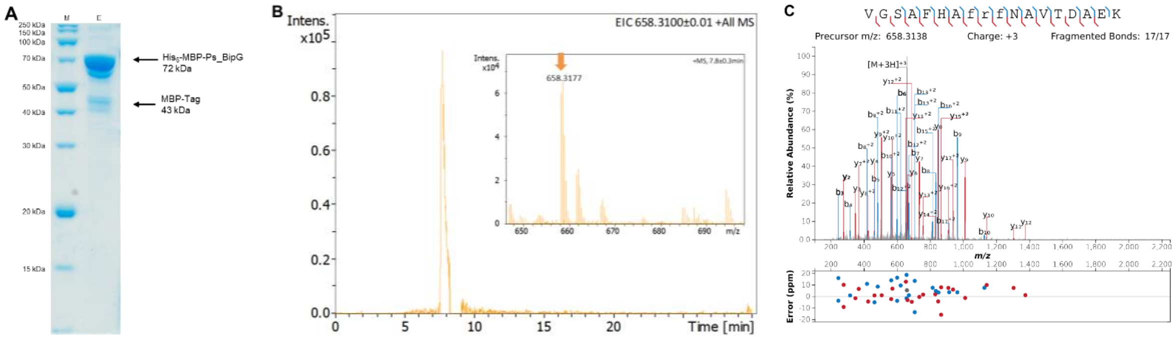

Figure S13. A. SDS-PAGE analysis of the purified His_6-MBP-Ps_BipG. **B.** LC-HRMS/MS analysis of the deguanidinated and di-hydroxylated Ps_BipA1 from an enzyme assay with Ps_BipG. The product peak (*m/z* 658.31, $[M+3H]^{3+}$) corresponds to additional hydroxylation. **C.** The MS/MS data matched to the calculated fragment pattern of the hydroxylated product.

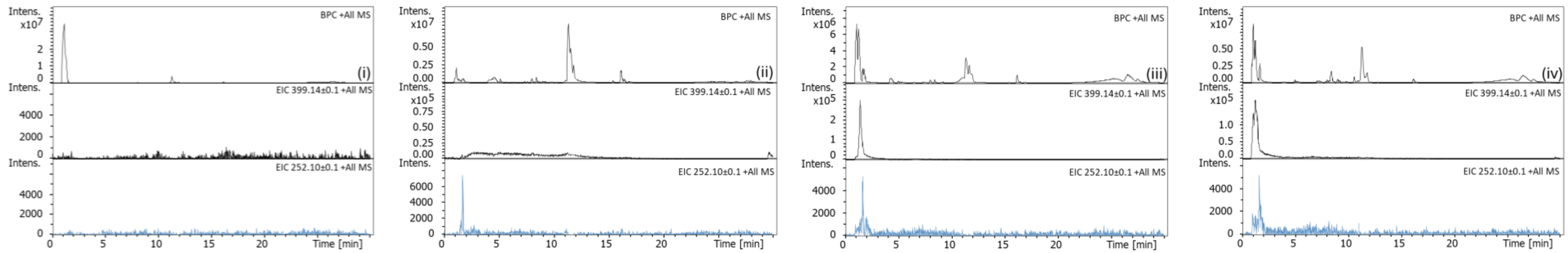

Figure S14. LC-HRMS/MS analysis of enzyme assays with MBP-Sg_BipD using various reducing systems, monitoring the cleavage of SAM (black; *m/z* 399.42, $[M+H]^+$) and 5'-dAdoH (blue: *m/z* 252.10, $[M+H]^+$). Shown are (i) the control, and the assays using (ii) titanium(III) citrate, (iii) flavodoxin/flavodoxin reductase and NADPH, and (iv) MV/NADPH.

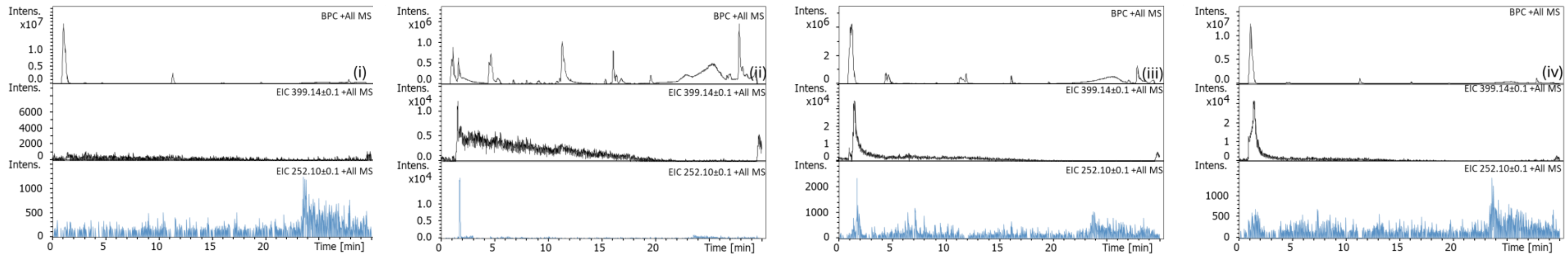

Figure S15. LC-HRMS/MS analysis of enzyme assays with His_8-Sg_BipD using various reducing systems, monitoring the cleavage of SAM (black; *m/z* 399.42, $[M+H]^+$) and 5'-dAdoH (blue: *m/z* 252.10, $[M+H]^+$). Shown are (i) the control, and the assays using (ii) titanium(III) citrate, (iii) flavodoxin/flavodoxin reductase and NADPH, and (iv) MV/NADPH.

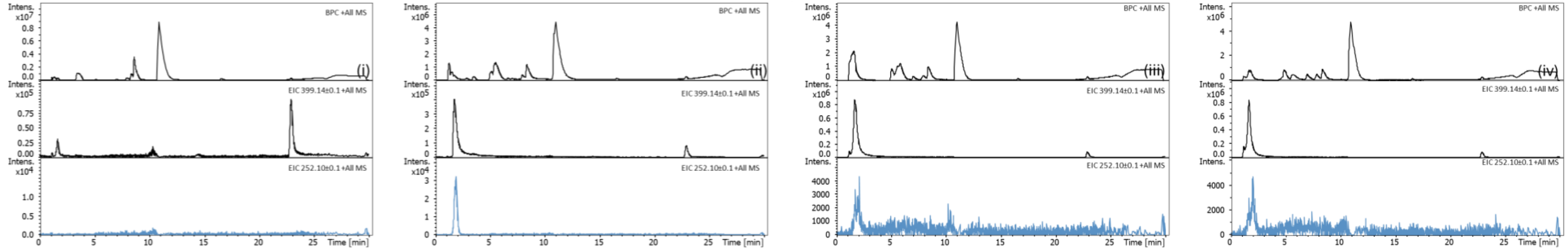

Figure S16. LC-HRMS/MS analysis of enzyme assays with His$_8$-Ps_BipD using various reducing systems, monitoring the cleavage of SAM (black; *m/z* 399.42, [M+H]$^+$) and 5'-dAdoH (blue: *m/z* 252.10, [M+H]$^+$). Shown are (i) the control, and the assays using (ii) titanium(III) citrate, (iii) flavodoxin/flavodoxin reductase and NADPH, and (iv) MV/NADPH.

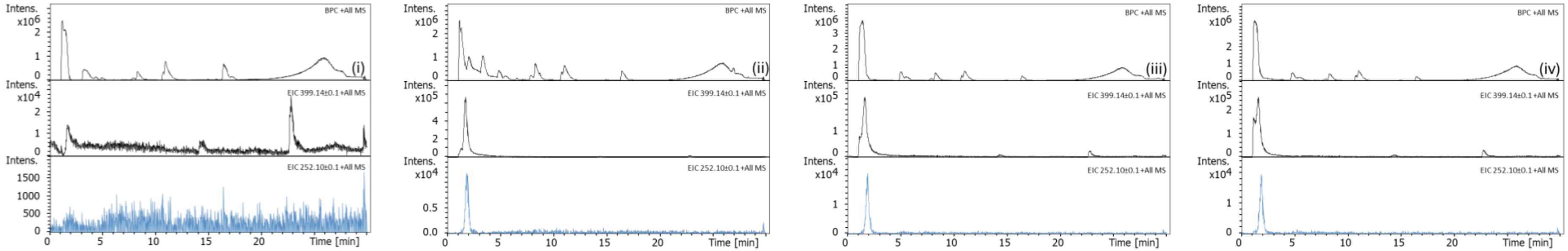

Figure S17 LC-HRMS/MS analysis of enzyme assays with His$_6$-MBP-Ps_BipD using various reducing systems, monitoring the cleavage of SAM (black; *m/z* 399.42, [M+H]$^+$) and 5'-dAdoH (blue: *m/z* 252.10, [M+H]$^+$). Shown are (i) the control, and the assays using (ii) titanium(III) citrate, (iii) flavodoxin/flavodoxin reductase and NADPH, and (iv) MV/NADPH.

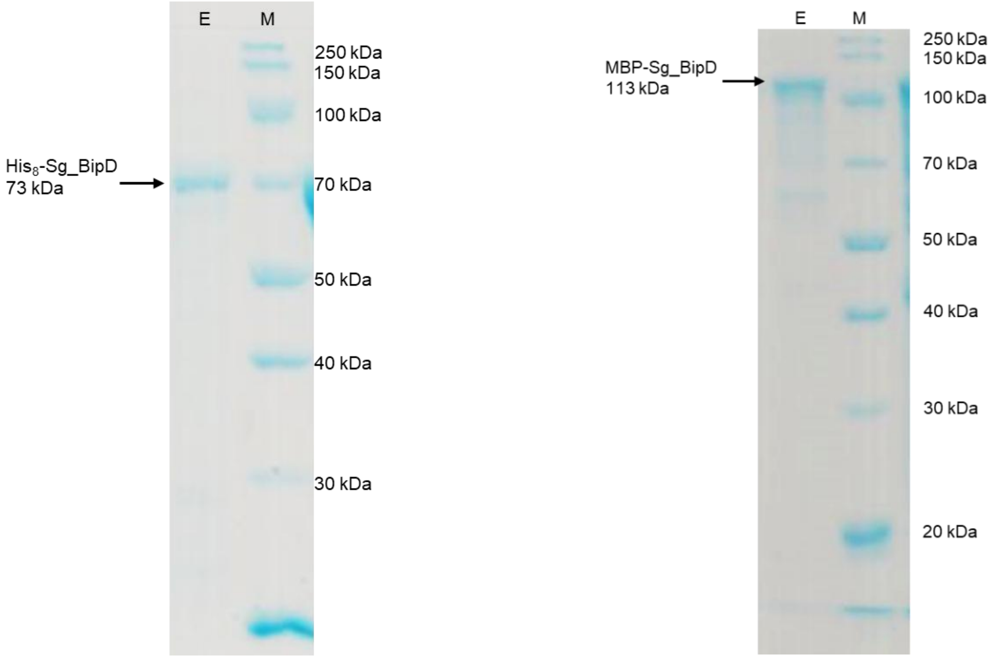

Figure S18. SDS-PAGE analysis of the His_8-tagged (left) and MBP-tagged (right) versions of Sg_BipD. The molecular weight marker is shown in both gels (M). The purified His_8-Sg_BipD appears at approximately 73 kDa, while the MBP-tagged version appears at approximately 113 kDa, consistent with the expected sizes of the respective fusion proteins.

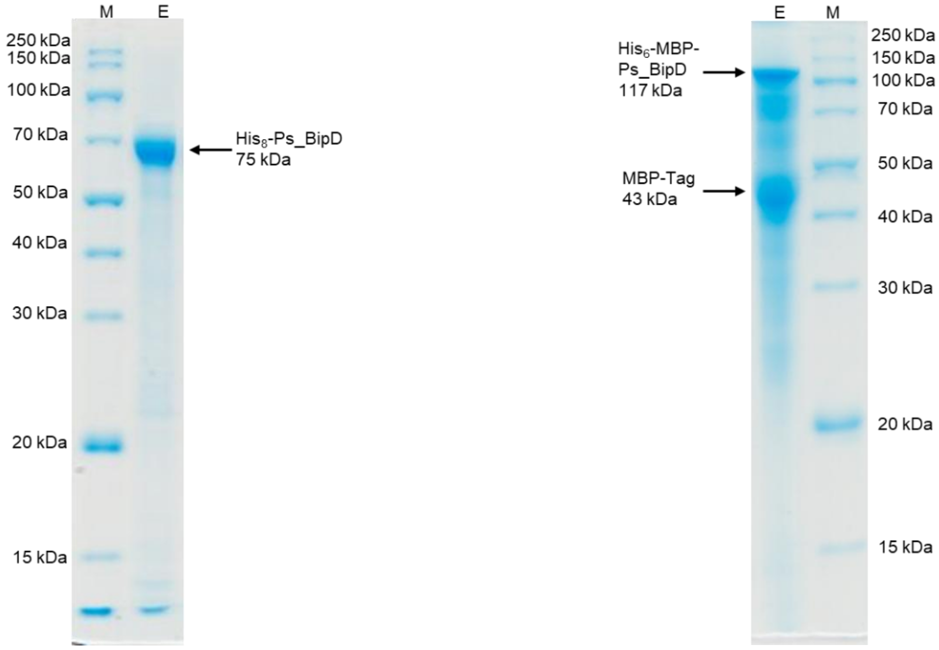

Figure S19. SDS-PAGE analysis of the His_8-tagged (left) and His_6-MBP-tagged (right) versions of Ps_BipD. The molecular weight marker is shown in both gels (M). The purified His_8-Ps_BipD appears at approximately 75 kDa, while the MBP-tagged version appears at approximately 117kDa, consistent with the expected sizes of the respective fusion proteins.

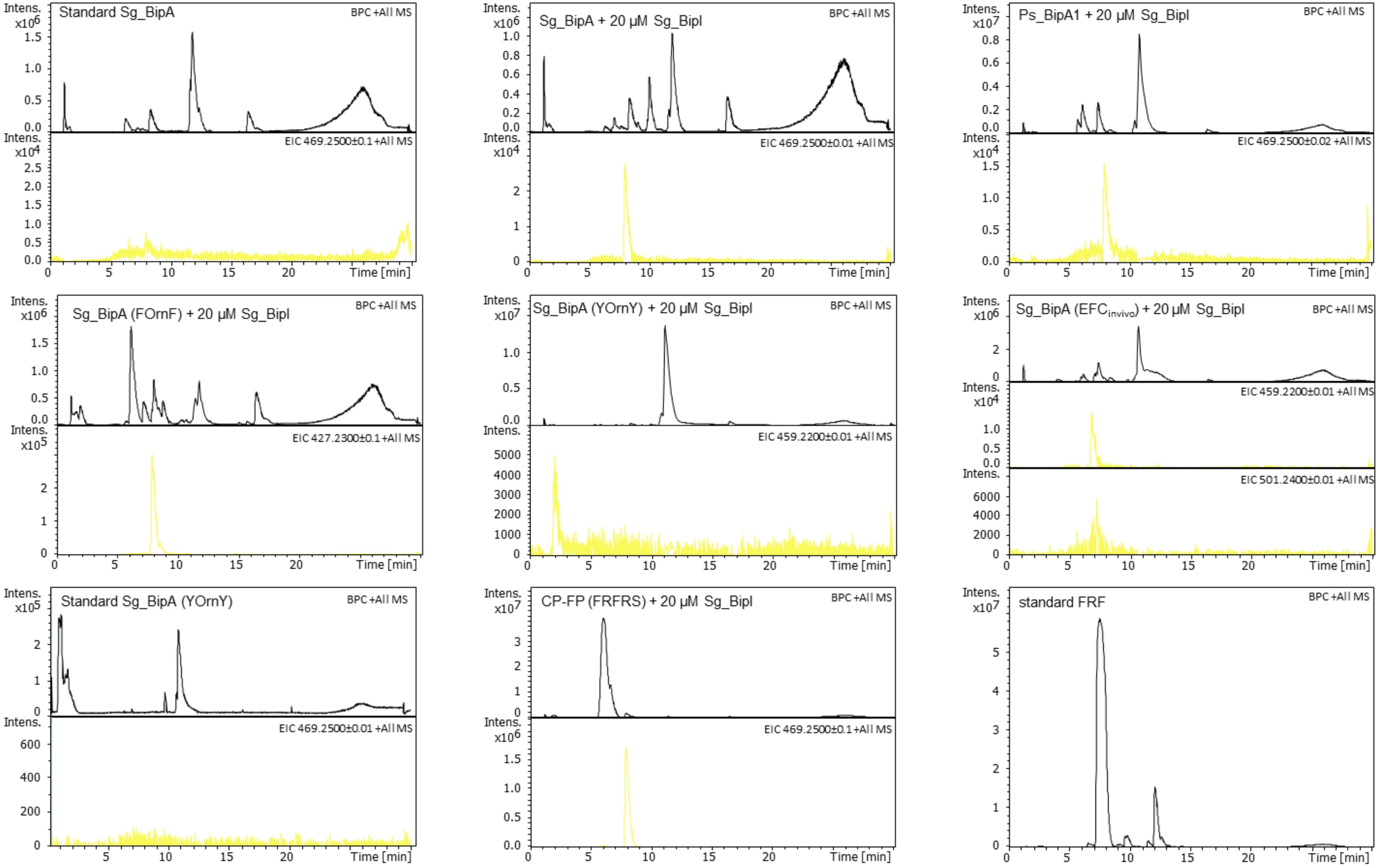

Figure S20. Enzymatic assays with Sg_BipI and various substrates analyzed by LC-HRMS/MS. Each pair of chromatograms depicts the full chromatogram (BPC, top, black) and the EIC (bottom, yellow) for the expected, released CP, corresponding to both, *N*- and *C*-terminal proteolysis.

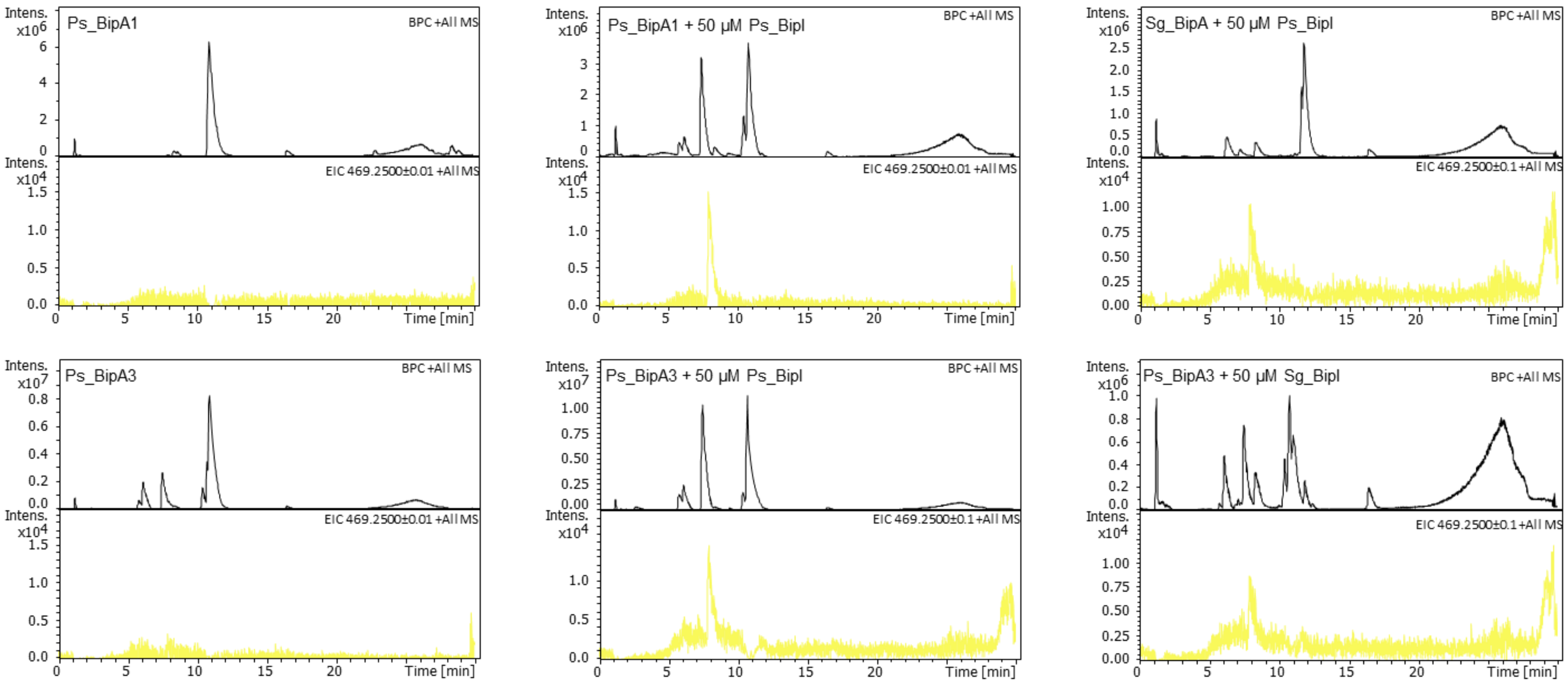

Figure S21. Enzymatic assays with Ps_BipI and various substrates analyzed by LC-HRMS/MS. Each pair of chromatograms depicts the full chromatogram (BPC, top, black) and the EIC (bottom, yellow) for the expected, released CP, corresponding to both, *N*- and *C*-terminal proteolysis.

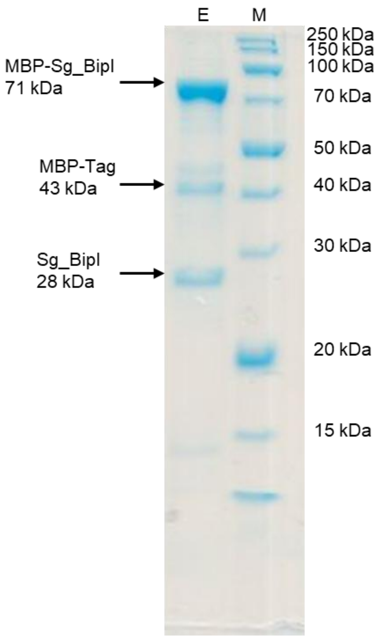

Figure S22. SDS-PAGE analysis of purified MBP-Sg_BipI (expected size of fusion protein ~43 kDa). M: molecular weight marker.

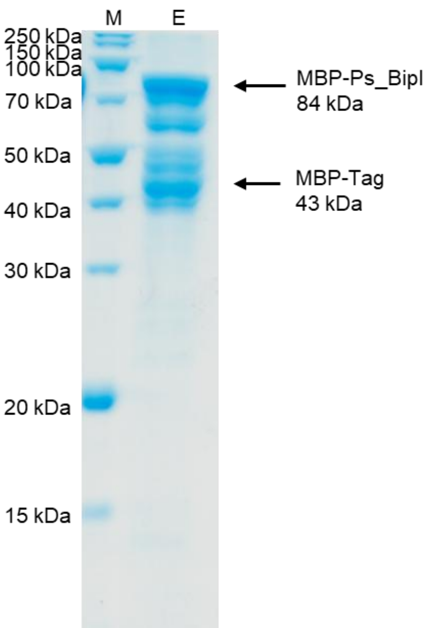

Figure S23. SDS-PAGE analysis of purified MBP-tagged Ps_BipI (expected size of fusion protein ~84 kDa). M: molecular weight marker.

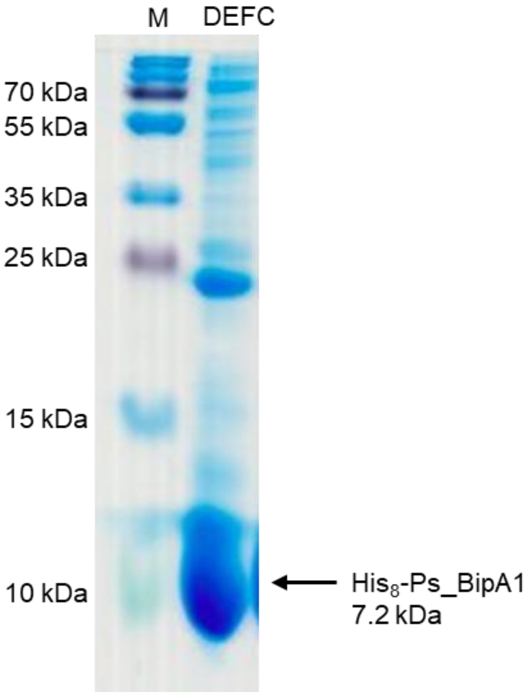

Figure S24. SDS-PAGE analysis of *in vivo* expression of pACYC::*Ps_bipDEFC* in co-expression with pHis$_8$::*Ps_bipA1*. Purification of His$_8$-Ps_BipA1, where a strong band at 7.2 kDa was visible. A molecular weight marker was included for reference (M).

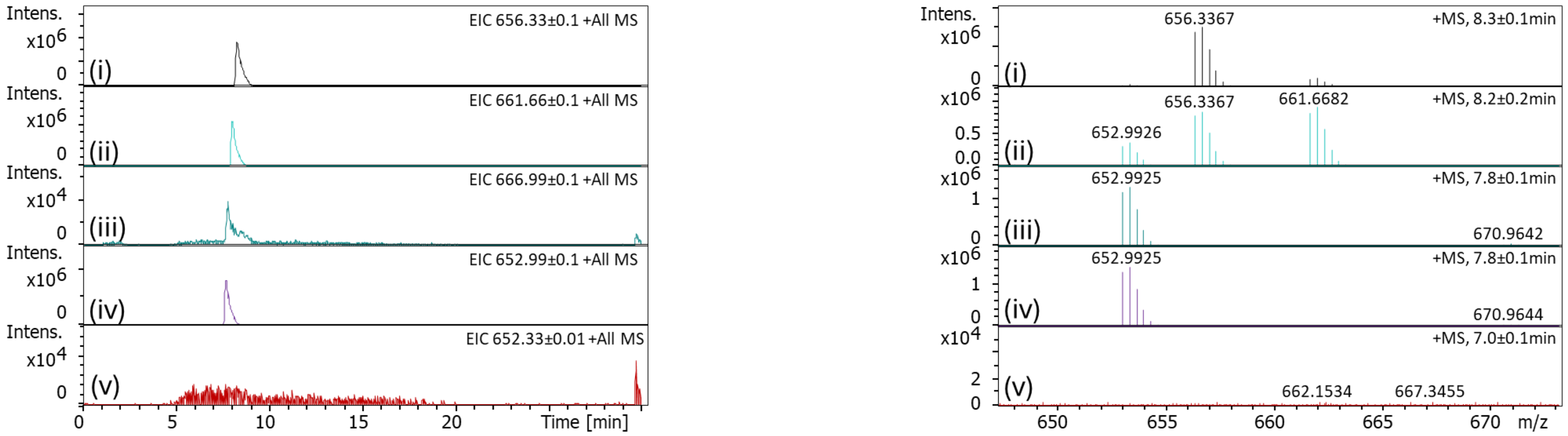

Figure S25. *In vivo* co-expression of construct pACYC::*Ps_bipEFC* with pHis$_8$::*Ps_bipA1*, illustrating the stepwise enzymatic transformation of the precursor peptide. LC-HRMS analyses (left, EICs of expected products after GluC digest) with corresponding MS data (right) are depicted: (i) unmodified precursor peptide (*m/z* 656.33, $[M+3H]^{3+}$), (ii) mono-hydroxylated intermediate (*m/z* 661.66, $[M+3H]^{3+}$), (iii) di-hydroxylated product (*m/z* 666.99, $[M+3H]^{3+}$), and (iv) the deguanidinated CP (*m/z* 652.99, $[M+3H]^{3+}$). The putative biaryl coupling product was not detected, as expected (v; expected *m/z* 652.33, $[M+3H]^{3+}$).

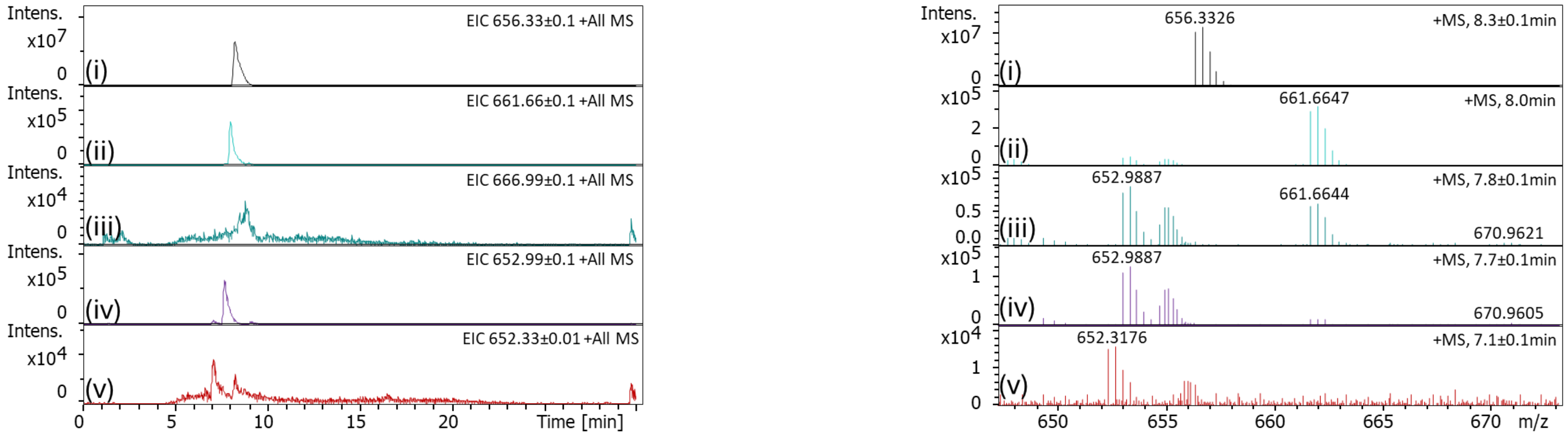

Figure S26. *In vivo* co-expression of construct pACYC::*Ps_bipDEFC* with pHis$_8$::*Ps_bipA1*, illustrating the stepwise enzymatic transformation of the precursor peptide. LC-HRMS analyses (left, EICs of expected products after GluC digest) with corresponding MS data (right) are depicted: (i) unmodified precursor peptide (*m/z* 656.33, $[M+3H]^{3+}$), (ii) mono-hydroxylated intermediate (*m/z* 661.66, $[M+3H]^{3+}$), (iii) di-hydroxylated product (*m/z* 666.99, $[M+3H]^{3+}$), and (iv) the deguanidinated CP (*m/z* 652.99 $[M+3H]^{3+}$), and the *C,C* coupled product (v: *m/z* 652.33, $[M+3H]^{3+}$).

Table S6. Nucleotide (nt) and amino acid (AA) sequences of genes and enzymes, respectively, used in this work. Underlined AA sequence parts correspond to the respective affinity tags introduced in the constructs.

pHis$_8$-TEV::*Ps_bipA*	nt	ATGAAACACCACCACCACCACCACCACCACGGTGGTGAGAATCTTTATTTTCAGGGAATGACTAAGC<br>AAGTGGAGAATACACAAGCGGAAAAGCTAGTGGAAAATCTTGAAGTAATCGAAGCTAAAGAGCTGGA<br>AGTGGGTAGTGCATTTCATGCATTCCGTTTCAATGCAGTGACGGACGCAGAAAAATAG
	AA	MKHHHHHHHHGGENLYFQGMTKQVENTQAEKLVENLEVIEAKELEVGSAFHAFRFNAVTDAEK*
pACYC_Duet::*Ps_bipEFC*		ATGGGCAGCAGCAGCACAGTCTATCAGAAAATAAAATTTCCGAATGTAAAGGAAAAACTGGGGCTTG<br>GTTTAGGCATGGATTTACCTTGGGGGGAAGATATAGGTTTTTCCAGCAAAGGGGACCATGACGACAT<br>CACGCCTAAGATGAAAGGTTTTTTTCAGAATTATAAAGACGAGTTTAATTATATTTTCTTTGCTTTTCAA<br>CCGAAAAAATAGAAGTGTTTTAAAAGCCGAGGACTACTTTGATGCCTATGATAGATTATTTGAAGCCAAT<br>CCACATCTAAAGGCGAGAGCATTCCATCAAACGATTTTAAATATGGGGGCTACCGAGTATTACAAAAA<br>AACAGAAATCATAGAATTCACAAATAAGATTATAGAAAGATATGACATAAGATGGATCGTAGAGGATCTT<br>GGCCTGTGGTCAATAAAAGGGAAAACCGTCCCTTTCCCTTTACCACCATATATGACACCAGCAGGATT<br>GGAAGCTTGTATTAAGAACATCAACGAATACCAATCTGAACTGGCTGTTCCAGTTTCAGTTGAATTTC<br>CTGGATTCACTGAAGGTACTAACTTCTTTATTGGGGAAATAAATGGATTTGATTATTTCAGAACATTGG<br>TCGAAGAGACAAACTCCCCTATCACTATAGATATTGGTCATATCCTTAGTTATCAATGGCTGTTAGGGA<br>ATACAAATGAAAAGATGTTCAATGGTTTAGAAAAATTGCCTTTTGAGAACTGTTTTGAATTGCATCTTTC<br>AGGTTGCCAAATTATTAAAGGGAAATTCAGAGATTTGCATCATGGAGTTTTAATGGATGAACAAATTCA<br>GCTTTTAGATTATTTGCTTCCTTTATGCCCTAATTTAAAAGCCATTACATATGAAGACCCTAAATATACAG<br>ATGAAGGAATATTAATTCCGAAGTCACAGAAAAATTATCAAAGAATGAAGGAGGCAGTTGAAAAATGG<br>CAAATGATTTAAAACCAAAGAATTATTCACTAGACGAGTTACTGTACAATTTATTATATGATTACCAATAT<br>AGAAATAACTTTTTAAATGATGAGTTTAATGAATTGAATTTATCAGCTGATAATTTAAATCACATAAAAAC<br>AATAGATAAAGAAGAGCTTGTAGCAACGGCTACAACGATTGTAAGAAATTTAATGAGTGGAAATATTGA<br>ACATAAAGGTGGCTTAAGAACTTCTTTTCCCGGTGTTTTTCAGGCTTTAGAAATCCTTGAAACCGATAT<br>AACGCTGTTAATGCATAAGTTCCTGGCGTCCAAACATTTCGAGTGCTACATGGAATTGCCCTATGCAG<br>GGGAGGGTACTTGTATCGAGGAAGCGTTTTACAATTATTTGTCTGAAAACGAAGAATTTATCCTTGCG<br>GCTGATAATAATCATTTACTATTAAAACACGAATTTTTAAATGCCATTTTATCTATACTAACAGTCAATAAA<br>CACCCATTTTTCAGAATAGATAGTGACCTGGTTAAAAATAATGGCCATATTTACTATGCGTACCAAACAT<br>ACTCCAAAGAAATATCCGAGGCACTGAGTGGAAAAAAAGTAGCTAGTGATTCCGAAAAGGTTATATGG<br>CTATATGCAGCGACCGAAAAAAATTTAATTAGGGGCCCTATTCATCCAAGTGTATTAGAAATGGTAGAA<br>ATAGGAGGTTCAAGGGAAATAAACCGCCAACAAAAGTTCAATCAGGACCAGATAGACTGGATTCTAAA<br>CTTAGGATTAATCAAGAATGATGGATAAAATATATGTATATGAGGAACATAGTGAAGTATTTTCATATTGG<br>GTCAATGAAATTCCTAGGAATAGTACCTTGGTTTATTTTGATCAACACTTAGATTTAAAGTTCATAGAAA<br>ATTCAAAAATGAAAAGAATCAGTCAGTTCATTAAAGAGGGTCTATGTATTGATGAATTGAAAAAAGATAT<br>TCCTTGTAGGGAAGATGGAAGATATTCTTATGGGATCGATGACTTTTTGTACGCTGTAATACAAGAGG<br>GCCTTTTTCGGAAAATAATATGGGTATATCCTCGTATGTTAGGAGAAAAAGGGTTTAGTGAATTACTTT<br>GGGAGCTATTATCCCTTGTACCCAACCATGGAAAAGAATTCATATCAAGCTTTAGAAACGGAAAAAATT<br>CTGCTTCTGTACAATTAAATAATATTGAGTTACATGTCACAACTATTGAATATCTAGGCGAATTCATAATT<br>AATGAACAGGTTATTGTAGATATCGATTTGGATTATTTTTATGATCCCAAGACTAATGATTTATCGAATGA<br>TATAGACGAAACCTTACATGTATTGAACAATCTAGGCCTCTTTAATCAAGTGAAGACAATGTCCTATTCT<br>ATCAAAAGTGGGTTTCTACCTGAAAGTTTCAGGTGGATAGGGAAATATATAGCAGGACAAATGGGGA<br>GGGAGATTGCATATTCCAAGGAAGATAAGTTTTCTCCGAAGGTAACGATGGAGAAAATTTCATCGAAT<br>AAAAAGTTGAGTCTAGCAGAAATAGACGAATTAAACTTTGAATTGAGACCATTAGGAGCAATAGGTTG<br>GAAATTGAAATCGATTCTCTATACCCAGTCTGGTGAAATTGAACTTGCTAAGAATTGCTATCAAAAAGC<br>GATTGAAAAAGGTGATGGTTCATATTGGGCTGCCTATGTTTTAGGCATTCACTTTTTCAAGGATAGGAA<br>TTATGAAGAGGCTTTAAATTGGTTTAATAAAACTGGAAATATAGTCGATACCATCGAGGCACATGGCCT<br>AATATTAAGTTTAATTTGTTGCCTTCGTTTGGAGCTTTATCAAAAAGGATATGATTTATCAAAAGAGTGC<br>ATCGAATTATTACCTATGAGAATAGAGGGGTATATATTGGGTGAAGTATTCGCAGCAAAACTAGGAATG<br>AAATTAATGGATTTTGATGGCAAGATCCATGACGATCCTTCGCAATTAGTAATTAGTCGGGCTGATACG<br>AAACATGAACAATGA
pACYC_Duet::*Ps_bipDEFC*	nt	ATGGGCAGCAGCGAAAATTCAGATGCAATGATAGTTTTTCCTCCTCTAACTGAAGCCAGGCTCTTTCC<br>ATATCTAAGTTTACCTATGATTACTAGTTTTTTGAGGAACAAGGGAATGAGTGTAAGTCAAATTGATTTA<br>AATATTGAATTATGCCATACCCTCTTTAGTGAAGATTGTTTAGCTGAATATGTGAGCATCAATGAAAACG<br>GAAGTAAAGACTTAAAATTGATCTATAAAGTCGAAATGGCTAAATACTTATACAAAGAACAAAAGCAAC<br>TTTACAATAATGTATTCATTGAGAAAAGATCTTCTGATTCATTGAAGTATGACGTTCGGTTGGTGAGAC<br>AAGGCATAGAACTATTACTATTAAATTCGTTTTTAAAACTTGAGATTACCTCATTGGAGGAAATCCTTGA<br>ATTGGTAAGGAATTTTAGTTGGAAAAAAAGTGATATTGCAACAAAGGTTTTATACGAAAATATCAAAGA<br>AAAAATTCTTAACGATAAGCCCAAAATCTTCGCGATTTCTATTGCATATTATAGCCAAATATTACCTTCCC<br>TTTTAATATGCAAGTGGATTAGAGAACTTTCCCCTAATACTCACATTATTCTGGGTGGACAACAAATCAT<br>GATTCGTCAATCATCTTTCTTAAGCCTAAATGGATTAAATCAATTTGTGGATTCATTAGGAATATCAGCA<br>GGTGAAGAAACATTATTCATGTTAGACAGATATTTGAAAAACGATTGCCAAATCGGGAAGGTGCCGGA<br>TATTGTTTGGCTTAATGAGAAAAATGGTGAAAATATTCCATCTAAGTCTGCATATAGGATCACCGACGC<br>CTTACCACCTGACTTTTCTGATTTACCGTACAAAAACTATTTAGATGAAGAAGTGCATATGTCTTTGATT<br>ACATGTGTAGGGTGTTACTGGGGACGATGTACTTTTTGTTCATATGGTAATAGATCCAGAAAAGAAAAA<br>AGTTATCAGCAAAAAACGGCAAGGCAAGTAGCAAACGAGTGCGAGGATATCATTAACAAATACGGGG<br>TTAATAGAATTAATTTCATAGATGAGAATACTAATTTGAGGCTTGTCGTCAATGCAGTCAAAATACTTAA<br>ATCCAGAGGATATGAAATTAATTTCAGTACACGCAACCGTCTTGAGAATGTGTTGTTAGACGCTGAATT<br>TTGTTTTGAGCTAAGTAATTTAGGTTGTATTTTAATGTCTGTTGGCTATGAAACCAATTCTCAAAGGATT<br>TTAGATTCCTTGGATAAGGGAGTGCAATCTAGTAATTATCAACAGATAATAGATAACTTACATAATGCCA<br>ATATCACGCTGCGAATGTCCATAATTGGTGGCTTACCTGGTGAAACCGAGGATGAAATTAAATGTTCT<br>GAGGAATTTTTACTTAAAAATCAAGATAAGATTGGCATCGACGTCATGCAAATGCTCGTTTTAGAACCA<br>GGTACCTATATTTATGAAGATACAAATAATCCTGATATCCATATTCAATCAAGCAAGAATCTAAGAGGAA<br>ACAAATTACTCAATTATGGGATGGGTAGAATGGGAGCTACATTTCAATACTCTGACGGAAAAACGTTT<br>GAAGAAAAGCTGAATAGATTCTTGCAATTGCACAAAAATGTCAACCCCCAAAAGAATGATGAGCTACC<br>TCCTGATAAATACAAAACGGTAGGACAGGATATCACTTCAAATACCTTATTGATAAACCCATGGACTAA<br>AATCATTAAGTTAGATAGGACGTATATCATGGATTTTGTATGGCAAAGAGTCTTCTTAGTGCCAGAATC<br>CATAGAAGTATTCGGAAACACACTGATAATTAGCAAGGCTGACGATAAAAAGTACCTTGAGTATTTTGT<br>CGAAAAAGGTGTGATTAAGCTTAAATAAAATAACTTATGGGAGGAATGACAAATGAGCACAGTCTATCA<br>GAAAATAAAATTTCCGAATGTAAAGGAAAAACTGGGGCTTGGTTTAGGCATGGATTTACCTTGGGGG

GAAGATATAGGTTTTTCCAGCAAAGGGGACCATGACGACATCACGCCTAAGATGAAAGGTTTTTTTCA
GAATTATAAAGACGAGTTTAATTATATTTTCTTTGCTTTTCAACCGAAAAATAGAAGTGTTTTAAAAGCC
GAGGACTACTTTGATGCCTATGATAGATTATTTGAAGCCAATCCACATCTAAAGGCGAGAGCATTCCAT
CAAACGATTTTAAATATGGGGGCTACCGAGTATTACAAAAAAACAGAAATCATAGAATTCACAAATAAG
ATTATAGAAAGATATGACATAAGATGGATCGTAGAGGATCTTGGCCTGTGGTCAATAAAAGGGAAAAC
CGTCCCTTTCCCTTTACCACCATATATGACACCAGCAGGATTGGAAGCTTGTATTAAGAACATCAACG
AATACCAATCTGAACTGGCTGTTCCAGTTTCAGTTGAATTTCCTGGATTCACTGAAGGTACTAACTTCT
TTATTGGGGAAATAAATGGATTTGATTATTTCAGAACATTGGTCGAAGAGACAAACTCCCCTATCACTA
TAGATATTGGTCATATCCTTAGTTATCAATGGCTGTTAGGGAATACAAATGAAAAGATGTTCAATGGTTT
AGAAAAATTGCCTTTTGAGAACTGTTTTGAATTGCATCTTTCAGGTTGCCAAATTATTAAAGGGAAATT
CAGAGATTTGCATCATGGAGTTTTAATGGATGAACAAATTCAGCTTTTAGATTATTTGCTTCCTTTATGC
CCTAATTTAAAAGCCATTACATATGAAGACCCTAAATATACAGATGAAGGAATATTAATTCCGAAGTCAC
AGAAAAATTATCAAAGAATGAAGGAGGCAGTTGAAAAATGGCAAATGATTTAAAACCAAAGAATTATTC
ACTAGACGAGTTACTGTACAATTTATTATATGATTACCAATATAGAAATAACTTTTTAAATGATGAGTTTAA
TGAATTGAATTTATCAGCTGATAATTTAAATCACATAAAAACAATAGATAAAGAAGAGCTTGTAGCAACG
GCTACAACGATTGTAAGAAATTTAATGAGTGGAAATATTGAACATAAAGGTGGCTTAAGAACTTCTTTT
CCCGGTGTTTTTCAGGCTTTAGAAATCCTTGAAACCGATATAACGCTGTTAATGCATAAGTTCCTGGC
GTCCAAACATTTCGAGTGCTACATGGAATTGCCCTATGCAGGGGAGGGTACTTGTATCGAGGAAGCG
TTTTACAATTATTTGTCTGAAAACGAAGAATTTATCCTTGCGGCTGATAATAATCATTTACTATTAAAACA
CGAATTTTTAAATGCCATTTTATCTATACTAACAGTCAATAAACACCCATTTTTCAGAATAGATAGTGACC
TGGTTAAAAATAATGGCCATATTTACTATGCGTACCAAACATACTCCAAAGAAATATCCGAGGCACTGA
GTGGAAAAAAAGTAGCTAGTGATTCCGAAAAGGTTATATGGCTATATGCAGCGACCGAAAAAAATTTA
ATTAGGGGCCCTATTCATCCAAGTGTATTAGAAATGGTAGAAATAGGAGGTTCAAGGGAAATAAACCG
CCAACAAAAGTTCAATCAGGACCAGATAGACTGGATTCTAAACTTAGGATTAATCAAGAATGATGGATA
AAATATATGTATATGAGGAACATAGTGAAGTATTTTCATATTGGGTCAATGAAATTCCTAGGAATAGTAC
CTTGGTTTATTTTGATCAACACTTAGATTTAAAGTTCATAGAAAATTCAAAAATGAAAAGAATCAGTCAG
TTCATTAAAGAGGGTCTATGTATTGATGAATTGAAAAAAGATATTCCTTGTAGGGAAGATGGAAGATATT
CTTATGGGATCGATGACTTTTTGTACGCTGTAATACAAGAGGGCCTTTTTCGGAAAATAATATGGGTAT
ATCCTCGTATGTTAGGAGAAAAAGGGTTTAGTGAATTACTTTGGGAGCTATTATCCCTTGTACCCAACC
ATGGAAAAGAATTCATATCAAGCTTTAGAAACGGAAAAAATTCTGCTTCTGTACAATTAAATAATATTGA
GTTACATGTCACAACTATTGAATATCTAGGCGAATTCATAATTAATGAACAGGTTATTGTAGATATCGATT
TGGATTATTTTTATGATCCCAAGACTAATGATTTATCGAATGATATAGACGAAACCTTACATGTATTGAAC
AATCTAGGCCTCTTTAATCAAGTGAAGACAATGTCCTATTCTATCAAAAGTGGGTTTCTACCTGAAAGT
TTCAGGTGGATAGGGAAATATATAGCAGGACAAATGGGGAGGGAGATTGCATATTCCAAGGAAGATAA
GTTTTCTCCGAAGGTAACGATGGAGAAAATTTCATCGAATAAAAAGTTGAGTCTAGCAGAAATAGACG
AATTAAACTTTGAATTGAGACCATTAGGAGCAATAGGTTGGAAATTGAAATCGATTCTCTATACCCAGT
CTGGTGAAATTGAACTTGCTAAGAATTGCTATCAAAAAGCGATTGAAAAAGGTGATGGTTCATATTGG
GCTGCCTATGTTTTAGGCATTCACTTTTTCAAGGATAGGAATTATGAAGAGGCTTTAAATTGGTTTAATA
AAACTGGAAATATAGTCGATACCATCGAGGCACATGGCCTAATATTAAGTTTAATTTGTTGCCTTCGTTT
GGAGCTTTATCAAAAAGGATATGATTTATCAAAAGAGTGCATCGAATTATTACCTATGAGAATAGAGGG
GTATATATTGGGTGAAGTATTCGCAGCAAAACTAGGAATGAAATTAATGGATTTTGATGGCAAGATCCA
TGACGATCCTTCGCAATTAGTAATTAGTCGGGCTGATACGAAACATGAACAATGA

pMAL-c5x::*Sg_bipC* nt

<u>ATGAAAATCGAAGAAGGTAAACTGGTAATCTGGATTAACGGCGATAAAGGCTATAACGGTCTCGCTGA</u>
<u>AGTCGGTAAGAAATTCGAGAAAGATACCGGAATTAAAGTCACCGTTGAGCATCCGGATAAACTGGAA</u>
<u>GAGAAATTCCCACAGGTTGCGGCAACTGGCGATGGCCCTGACATTATCTTCTGGGCACACGACCGC</u>
<u>TTTGGTGGCTACGCTCAATCTGGCCTGTTGGCTGAAATCACCCCGGACAAAGCGTTCCAGGACAAG</u>
<u>CTGTATCCGTTTACCTGGGATGCCGTACGTTACAACGGCAAGCTGATTGCTTACCCGATCGCTGTTG</u>
<u>AAGCGTTATCGCTGATTTATAACAAAGATCTGCTGCCGAACCCGCCAAAAACCTGGGAAGAGATCCC</u>
<u>GGCGCTGGATAAAGAACTGAAAGCGAAAGGTAAGAGCGCGCTGATGTTCAACCTGCAAGAACCGTA</u>
<u>CTTCACCTGGCCGCTGATTGCTGCTGACGGGGGTTATGCGTTCAAGTATGAAAACGGCAAGTACGA</u>
<u>CATTAAAGACGTGGGCGTGGATAACGCTGGCGCGAAAGCGGGTCTGACCTTCCTGGTTGACCTGAT</u>
<u>TAAAAACAAACACATGAATGCAGACACCGATTACTCCATCGCAGAAGCTGCCTTTAATAAAGGCGAAA</u>
<u>CAGCGATGACCATCAACGGCCCGTGGGCATGGTCCAACATCGACACCAGCAAAGTGAATTATGGTG</u>
<u>TAACGGTACTGCCGACCTTCAAGGGTCAACCATCCAAACCGTTCGTTGGCGTGCTGAGCGCAGGTA</u>
<u>TTAACGCCGCCAGTCCGAACAAAGAGCTGGCAAAAGAGTTCCTCGAAAACTATCTGCTGACTGATGA</u>
<u>AGGTCTGGAAGCGGTTAATAAAGACAAACCGCTGGGTGCCGTAGCGCTGAAGTCTTACGAGGAAGA</u>
<u>GTTGGTGAAAGATCCGCGTATTGCCGCCACTATGGAAAACGCCCAGAAAGGTGAAATCATGCCGAA</u>
<u>CATCCCGCAGATGTCCGCTTTCTGGTATGCCGTGCGTACTGCGGTGATCAACGCCGCCAGCGGTCG</u>
<u>TCAGACTGTCGATGAAGCCCTGAAAGACGCGCAGACTAATTCGAGCTCGAACAACAACAACAATAAC</u>
<u>AATAACAACAACCTCGGGATCGAGGGAAGGATTTCACATATGTCCATGGGC</u>ATGCCGGTGGACGTCG
ATCTCTTCGAGGAACACGGCGAGGTTGCGGCACTCTGGCCACACCGCCCCTACCACGGCCGTACG
GTCGTGTGCTTCGACCGCCATCTCGACCTCAAGCCCTTGGCGCCGGGGGGCGAGGAGGCGCTGC
GCGCCACCGCCGACGGGAACGTCTCCCCGGCCGAGCTGGTGCGCAGGCTCCCTGTCCGCGGGGT
GCCCGGCGCCTTCGGTCTCGACGACTTCTGGTCCGCCGCCGCAGTGGTCGCCGGCCTCACCGAC
CTGGTGTGGGTGCCCAGTTGGCGGTCGTACGACGGCTGGCAGGCGCACGCGGTGGACTCCGTGT
CCCTCATCACGACCGGCGGCAGACCGACCCGGCCCAGCACGCGGCCGTGCTGTCTGACGGTCAC
CCTGTGCGGCGTCAGGCTGGCCGTCGTACCACCCGATCTCCTGGCCGGGCACCTGGACCGGCATG
TGCACACGGACGTCGTGACCGACATCGACCTGGACTGGCTGGTCGACGAGCACGGCAGGTTCGAG
CACTCCGCGCAGGACCTGGCCGAACTCGTCGGCGTCTGCGGCGGCGAGCTGGCCGCGATGACCT
GGTCGACCCGGTCCGGGTTCCTGCCCTCCGAGTACCGGACCGTGGGAGCGGACGTCGCCGCACG
GCTCGGGCTGCGCGCCCGGGAGTCCTCCTTCCTGCCCGCCACGCCCTGGCCGGAGGACCTGATG
CTGCGAGTCCACCGGGGCACCGCGGCCCCCGCGGCCGGACCGGCTGACGAGGAGGGAGGGGTG
GAGCAGGGCATCGCCGTCGCCCTGCACGGCCTGGCCCAGGCCGGTCTCAGTCCCGACCGGGCAC
AGGAGTGTTTCGAGCAGGCGGCCGGGCACGGCTACCACTCCAGCTGGCTCGCCTACAAGATCGGC
GCAGCCCGCTATGCCAACGGCGACCATCGCACGGCACGCCAGTACCTGCGGGAGGCCGTCCGCC
TCGACCCGCAGGACACCCTCGGCGCACACGCCCGGATCATGGGTGCCCGAGCCACCCTGCGCCT
GGAGGGACCGGCCGCCGCCCTGTCCGAATTCCAGGCTCTCGGCGCCGAGTTGCCGCTGCGCAGA
GGCGTCTGGAAGACGATACGGATGCTTGCCCGCGCCGAGGGCGACATGGACACGGCACGGACCG
CCGAGGGCCAACTGCGGCTGCTGGACCGGCTGTCCGGGCCCGGCGCCGCCGAACCGGAGGTGG
AGGACGCGTGA

AA

<u>MKIEEGKLVIWINGDKGYNGLAEVGKKFEKDTGIKVTVEHPDKLEEKFPQVAATGDGPDIIFWAHDRFGG</u>
<u>YAQSGLLAEITPDKAFQDKLYPFTWDAVRYNGKLIAYPIAVEALSLIYNKDLLPNPPKTWEEIPALDKELKA</u>
<u>KGKSALMFNLQEPYFTWPLIAADGGYAFKYENGKYDIKDVGVDNAGAKAGLTFLVDLIKNKHMNADTDY</u>
<u>SIAEAAFNKGETAMTINGPWAWSNIDTSKVNYGVTVLPTFKGQPSKPFVGVLSAGINAASPNKELAKEFL</u>
<u>ENYLLTDEGLEAVNKDKPLGAVALKSYEEELVKDPRIAATMENAQKGEIMPNIPQMSAFWYAVRTAVINAA</u>
<u>SGRQTVDEALKDAQTNSSSNNNNNNNNNNLGIEGRISHMSMG</u>MPVDVDLFEEHGEVAALWPHRPYHG

RTVVCFDRHLDLKPLAPGGEEALRATADGNVSPAELVRRLPVRGVPGAFGLDDFWSAAAVVAGLTDLVW
VPSWRSYDGWQAHAVDSVSLITTGGRPTRPSTRPCCLTVTLCGVRLAVVPPDLLAGHLDRHVHTDVVT
DIDLDWLVDEHGRFEHSAQDLAELVGVCGGELAAMTWSTRSGFLPSEYRTVGADVAARLGLRARESSF
LPATPWPEDLMLRVHRGTAAPAAGPADEEGGVEQGIAVALHGLAQAGLSPDRAQECFEQAAGHGYHSS
WLAYKIGAARYANGDHRTARQYLREAVRLDPQDTLGAHARIMGARATLRLEGPAAALSEFQALGAELPL
RRGVWKTIRMLARAEGDMDTARTAEGQLRLLDRLSGPGAAEPEVEDA*

pMAL-c6t::*Ps_bipC*

nt

ATGAAAATCCACCATCACCACCACCACGAAGAAGGTAAACTGGTAATCTGGATTAACGGCGATAAAG
GCTATAACGGTCTCGCTGAAGTCGGTAAGAAATTCGAGAAAGATACCGGAATTAAAGTCACCGTTGA
GCATCCGGATAAACTGGAAGAGAAATTCCCACAGGTTGCGGCAACTGGCGATGGCCCTGACATTATC
TTCTGGGCACACGACCGCTTTGGTGGCTACGCTCAATCTGGCCTGTTGGCTGAAATCACCCCGGAC
AAAGCGTTCCAGGACAAGCTGTATCCGTTTACCTGGGATGCCGTACGTTACAACGGCAAGCTGATTG
CTTACCCGATCGCTGTTGAAGCGTTATCGCTGATTTATAACAAAGATCTGCTGCCGAACCCGCCAAAA
ACCTGGGAAGAGATCCCGGCGCTGGATAAAGAACTGAAAGCGAAAGGTAAGAGCGCGCTGATGTTC
AACCTGCAAGAACCGTACTTCACCTGGCCGCTGATTGCTGCTGACGGGGGTTATGCGTTCAAGTATG
AAAACGGCAAGTACGACATTAAAGACGTGGGCGTGGATAACGCTGGCGCGAAAGCGGGTCTGACCT
TCCTGGTTGACCTGATTAAAAACAAACACATGAATGCAGACACCGATTACTCCATCGCAGAAGCAGC
CTTTAATAAAGGCGAAACAGCGATGACCATCAACGGCCCGTGGGCATGGTCCAACATCGACACCAG
CAAAGTGAATTATGGTGTAACGGTACTGCCGACCTTCAAGGGTCAACCATCCAAACCGTTCGTTGGC
GTGCTGAGCGCAGGTATTAACGCCGCCAGTCCGAACAAAGAGCTGGCAAAAGAGTTCCTCGAAAAC
TATCTGCTGACTGATGAAGGTCTGGAAGCGGTTAATAAAGACAAACCGCTGGGTGCCGTAGCGCTG
AAGTCTTACGAGGAAGAGTTGGTGAAAGATCCGCGTATTGCCGCCACTATGGAAAACGCCCAGAAA
GGTGAAATCATGCCGAACATCCCGCAGATGTCCGCTTTCTGGTATGCCGTGCGTACTGCGGTGATCA
ACGCCGCCAGCGGTCGTCAGACTGTCGATGAAGCCCTGAAAGACGCGCAGACTAATTCGAGCTCG
AACAACAACAACAATAACAATAACAACAACCTCGGGGAGAACCTGTACTTCCAGATGCTGATGGGCA
TGATGGATAAAATATATGTATATGAGGAACATAGTGAAGTATTTTCATATTGGGTCAATGAAATTCCTAG
GAATAGTACCTTGGTTTATTTTGATCAACACTTAGATTTAAAGTTCATAGAAAATTCAAAAATGAAAAGA
ATCAGTCAGTTCATTAAAGAGGGTCTATGTATTGATGAATTGAAAAAAGATATTCCTTGTAGGGAAGAT
GGAAGATATTCTTATGGGATCGATGACTTTTTGTACGCTGTAATACAAGAGGGCCTTTTTCGGAAAATA
ATATGGGTATATCCTCGTATGTTAGGAGAAAAAGGGTTTAGTGAATTACTTTGGGAGCTATTATCCCTT
GTACCCAACCATGGAAAAGAATTCATATCAAGCTTTAGAAACGGAAAAAATTCTGCTTCTGTACAATTA
AATAATATTGAGTTACATGTCACAACTATTGAATATCTAGGCGAATTCATAATTAATGAACAGGTTATTGT
AGATATCGATTTGGATTATTTTTATGATCCCAAGACTAATGATTTATCGAATGATATAGACGAAACCTTAC
ATGTATTGAACAATCTAGGCCTCTTTAATCAAGTGAAGACAATGTCCTATTCTATCAAAAGTGGGTTTC
TACCTGAAAGTTTCAGGTGGATAGGGAAATATATAGCAGGACAAATGGGGAGGGAGATTGCATATTCC
AAGGAAGATAAGTTTTCTCCGAAGGTAACGATGGAGAAAATTTCATCGAATAAAAAGTTGAGTCTAGC
AGAAATAGACGAATTAAACTTTGAATTGAGACCATTAGGAGCAATAGGTTGGAAATTGAAATCGATTCT
CTATACCCAGTCTGGTGAAATTGAACTTGCTAAGAATTGCTATCAAAAAGCGATTGAAAAAGGTGATG
GTTCATATTGGGCTGCCTATGTTTTAGGCATTCACTTTTTCAAGGATAGGAATTATGAAGAGGCTTTAA
ATTGGTTTAATAAAACTGGAAATATAGTCGATACCATCGAGGCACATGGCCTAATATTAAGTTTAATTTG
TTGCCTTCGTTTGGAGCTTTATCAAAAAGGATATGATTTATCAAAAGAGTGCATCGAATTATTACCTATG
AGAATAGAGGGGTATATATTGGGTGAAGTATTCGCAGCAAAACTAGGAATGAAATTAATGGATTTTGAT
GGCAAGATCCATGACGATCCTTCGCAATTAGTAATTAGTCGGGCTGATACGAAACATGAACAATGA

AA

MKIHHHHHHEEGKLVIWINGDKGYNGLAEVGKKFEKDTGIKVTVEHPDKLEEKFPQVAATGDGPDIIFWA
HDRFGGYAQSGLLAEITPDKAFQDKLYPFTWDAVRYNGKLIAYPIAVEALSLIYNKDLLPNPPKTWEEIPAL
DKELKAKGKSALMFNLQEPYFTWPLIAADGGYAFKYENGKYDIKDVGVDNAGAKAGLTFLVDLIKNKHMN
ADTDYSIAEAAFNKGETAMTINGPWAWSNIDTSKVNYGVTVLPTFKGQPSKPFVGVLSAGINAASPNKEL
AKEFLENYLLTDEGLEAVNKDKPLGAVALKSYEEELVKDPRIAATMENAQKGEIMPNIPQMSAFWYAVRTA
VINAASGRQTVDEALKDAQTNSSSNNNNNNNNNNLGENLYFQMLMGMMDKIYVYEEHSEVFSYWVNEI
PRNSTLVYFDQHLDLKFIENSKMKRISQFIKEGLCIDELKKDIPCREDGRYSYGIDDFLYAVIQEGLFRKIIW
VYPRMLGEKGFSELLWELLSLVPNHGKEFISSFRNGKNSASVQLNNIELHVTTIEYLGEFIINEQVIVDIDLD
YFYDPKTNDLSNDIDETLHVLNNLGLFNQVKTMSYSIKSGFLPESFRWIGKYIAGQMGREIAYSKEDKFSP
KVTMEKISSNKKLSLAEIDELNFELRPLGAIGWKLKSILYTQSGEIELAKNCYQKAIEKGDGSYWAAYVLGI
HFFKDRNYEEALNWFNKTGNIVDTIEAHGLILSLICCLRLELYQKGYDLSKECIELLPMRIEGYILGEVFAAK
LGMKLMDFDGKIHDDPSQLVISRADTKHEQ*

pMAL-c5x::*Sg_bipD*

nt

ATGAAAATCGAAGAAGGTAAACTGGTAATCTGGATTAACGGCGATAAAGGCTATAACGGTCTCGCTGA
AGTCGGTAAGAAATTCGAGAAAGATACCGGAATTAAAGTCACCGTTGAGCATCCGGATAAACTGGAA
GAGAAATTCCCACAGGTTGCGGCAACTGGCGATGGCCCTGACATTATCTTCTGGGCACACGACCGC
TTTGGTGGCTACGCTCAATCTGGCCTGTTGGCTGAAATCACCCCGGACAAAGCGTTCCAGGACAAG
CTGTATCCGTTTACCTGGGATGCCGTACGTTACAACGGCAAGCTGATTGCTTACCCGATCGCTGTTG
AAGCGTTATCGCTGATTTATAACAAAGATCTGCTGCCGAACCCGCCAAAAACCTGGGAAGAGATCCC
GGCGCTGGATAAAGAACTGAAAGCGAAAGGTAAGAGCGCGCTGATGTTCAACCTGCAAGAACCGTA
CTTCACCTGGCCGCTGATTGCTGCTGACGGGGGTTATGCGTTCAAGTATGAAAACGGCAAGTACGA
CATTAAAGACGTGGGCGTGGATAACGCTGGCGCGAAAGCGGGTCTGACCTTCCTGGTTGACCTGAT
TAAAAACAAACACATGAATGCAGACACCGATTACTCCATCGCAGAAGCTGCCTTTAATAAAGGCGAAA
CAGCGATGACCATCAACGGCCCGTGGGCATGGTCCAACATCGACACCAGCAAAGTGAATTATGGTG
TAACGGTACTGCCGACCTTCAAGGGTCAACCATCCAAACCGTTCGTTGGCGTGCTGAGCGCAGGTA
TTAACGCCGCCAGTCCGAACAAAGAGCTGGCAAAAGAGTTCCTCGAAAACTATCTGCTGACTGATGA
AGGTCTGGAAGCGGTTAATAAAGACAAACCGCTGGGTGCCGTAGCGCTGAAGTCTTACGAGGAAGA
GTTGGTGAAAGATCCGCGTATTGCCGCCACTATGGAAAACGCCCAGAAAGGTGAAATCATGCCGAA
CATCCCGCAGATGTCCGCTTTCTGGTATGCCGTGCGTACTGCGGTGATCAACGCCGCCAGCGGTCG
TCAGACTGTCGATGAAGCCCTGAAAGACGCGCAGACTAATTCGAGCTCGAACAACAACAACAATAAC
AATAACAACAACCTCGGGATCGAGGGAAGGATTTCACATATGTCCATGGGCATCGACGCACTGCTCG
TCATGCCACCCGTCAGCGAAGCCGTGCAGTTCCCCTACCTGGCCCTGCCCCAGCTGACCGCGGCC
TGGACCGCCCGCGGCCACACGGTGCGCGCCCTCGACCTCAACCTGGAATACCGCGACGAGGTGC
TGGTGTGCCGCGGTCCGGCCCGGGAGCGTGCGGACGACGCCCCCGCGCGGAGCGCCGGGGACA
TCTACCGCAACGTCAGCGAGCGCTACCGGTCGGATCACGGAGCCCTGCTGCTCAGCACCGCCCGG
GACCGCTCCACGGGCTTCGCCCAGGAGACCGCCATCCAGGCCACCGGCCGTTATGTGGCGCAGCA
GGCCGCCCGGGACGGCTGGGTCATCTCCGGGACGCCCCGGCTGGGACAGCTGGACAAACTGGTC
GAGGCGGCCCGCGACACCTGGTCGGCGCGCTGGTGCACCGGCCGACTGGAGGCGGTGATCGAG
GAGGAGCGGCCACGGGTCCTGGCCTTCTCCGTTCCCTTCTTCAGCCAAGTGGTCCCCACCCTCGT
CCTGACCACCCTGCTCAAGCAGCGCCGGCCGGACCTGAGGATCGCGCTCGGTGGGCCGACCGTG
CAGATGTGGGCGGCCCTTCTGCTGCGCCGCACCGAGGCTGCCCGCTCGGTGGACCACTGGTGCC
TCGGCCACGGCGAGGACTTCCTCACGAACATCCTGCCGCCGCGATCGGCAGGCGCGGCGGCGTC
GGTCCGACAGCCGACTCCCCTGCCCGCACACGCCGCGGAGGGCCTGCGGGACGCCTTCGTGCTC
AACGATCAGCCGATGCCGGACTTCGGCCAGTTCGACTTCGCCGACTACAGCAACCAGGCCCACCA
GTTCCCCTATCGCCTCACCGTCGGCTGCTACTGGGGCAGATGCACGTTCTGCTCGTACGGCAACCG

		CTACCGCGACGCCCGCGCTTTCCAGCAGATCCACCCCGACACCGCGGCCGACCACCTGATCGCGC<br>TCTCCGAACGGCTCGGCATCACCGATGTGGCGGTGGCCGACGAGAACACCGGGCTGCGGCATCTG<br>CTGCGGGTCATGGAGGCGGTACGCCGCAGGGGCGCCGAGCTGACGTTCCGGGTCCGGGCGCGG<br>CTGGAACCGGAGCTCGCCGACCCCGGCTTCTGTCGTCGGCTGCGGGAGTCGGGCTGTGTGCAGC<br>TCTCGACGGGATTCGAGACCGCCCGTCAGGAGATCCTCGACTCCCTGGGCAAGGGACAGGACGCG<br>GCGCACGCCGAACGCGCGGTGCTCAACCTCACCCGTGCGGGCATCGTCACCAACCTGTCCTTCAT<br>GGACGGCTATGACCATCCGAACGCCGTCGACGGCTACCACGACACCGTCGGTGTGATCCAGCGGC<br>ACCCGGCGGAGCTGGGCCTGGACACCATGCAGCTCGTGGTGGCCGAACCCGGCAGCTACCTGTG<br>GGCGCAACGAGAGCGGCCCGACGACGAGCCTCTGGTCACCAACGACGGTCTGGCGTTCGCCGCG<br>GGACGGGTGGGTGGAGCGGTCCTGGACGACACCGCGACCGAGGCCGCCAGGCAGCGACTGCTG<br>CGGATGACCGTCGAGGCGGTCCCGGACGCCGAACGGGCCGGCCGGCCGGACCTGCCGCACCGT<br>GGCGGCTCGCCGCCCTCCGAGGACCGGGCGGCCCACCGGCGGCCCGCGCCGGGGCCGGCGCC<br>GGCGGCCCGCCCGCGCTTCGCGGTCGCCCTGGAGCAGGTGGGCGGCCGCTGGTTCCTGGCGGA<br>CGTGGCCTGGCCCCGGATGGCAGCCGTGCCTCCTGCCCTCGCCCCCGACCGGGACGGCCGGTTC<br>ACGGCCGACGACCCGGCCACCCACCGCTGGCTGCACCGGATGCTCGACAAACGCCTGCTGGTGC<br>CGGATCCGTCGGCGCCCGACGCCCTCGAAGGGAGGAGTGCGTGA
	AA	MKIEEGKLVIWINGDKGYNGLAEVGKKFEKDTGIKVTVEHPDKLEEKFPQVAATGDGPDIIFWAHDRFGG<br>YAQSGLLAEITPDKAFQDKLYPFTWDAVRYNGKLIAYPIAVEALSLIYNKDLLPNPPKTWEEIPALDKELKA<br>KGKSALMFNLQEPYFTWPLIAADGGYAFKYENGKYDIKDVGVDNAGAKAGLTFLVDLIKNKHMNADTDY<br>SIAEAAFNKGETAMTINGPWAWSNIDTSKVNYGVTVLPTFKGQPSKPFVGVLSAGINAASPNKELAKEFL<br>ENYLLTDEGLEAVNKDKPLGAVALKSYEEELVKDPRIAATMENAQKGEIMPNIPQMSAFWYAVRTAVINAA<br>SGRQTVDEALKDAQTNSSSNNNNNNNNNNLGIEGRISHMSMGIDALLVMPPVSEAVQFPYLALPQLTAA<br>WTARGHTVRALDLNLEYRDEVLVCRGPARERADDAPARSAGDIYRNVSERYRSDHGALLLSTARDRST<br>GFAQETAIQATGRYVAQQAARDGWVISGTPRLGQLDKLVEAARDTWSARWCTGRLEAVIEEERPRVLAF<br>SVPFFSQVVPTLVLTTLLKQRRPDLRIALGGPTVQMWAALLLRRTEAARSVDHWCLGHGEDFLTNILPPR<br>SAGAAASVRQPTPLPAHAAEGLRDAFVLNDQPMPDFGQFDFADYSNQAHQFPYRLTVGCYWGRCTFC<br>SYGNRYRDARAFQQIHPDTAADHLIALSERLGITDVAVADENTGLRHLLRVMEAVRRRGAELTFRVRARL<br>EPELADPGFCRRLRESGCVQLSTGFETARQEILDSLGKGQDAAHAERAVLNLTRAGIVTNLSFMDGYDH<br>PNAVDGYHDTVGVIQRHPAELGLDTMQLVVAEPGSYLWAQRERPDDEPLVTNDGLAFAAGRVGGAVLD<br>DTATEAARQRLLRMTVEAVPDAERAGRPDLPHRGGSPPSEDRAAHRRPAPGPAPAARPRFAVALEQVG<br>GRWFLADVAWPRMAAVPPALAPDRDGRFTADDPATHRWLHRMLDKRLLVPDPSAPDALEGRSA*
pHis$_8$-TEV::*Sg_bipD*	nt	ATGAAACACCACCACCACCACCACCACCACGGTGGTGAGAATCTTTATTTTCAGGGAATGATCGACG<br>CACTGCTCGTCATGCCACCCGTCAGCGAAGCCGTGCAGTTCCCCTACCTGGCCCTGCCCCAGCTG<br>ACCGCGGCCTGGACCGCCCGCGGCCACACGGTGCGCGCCCTCGACCTCAACCTGGAATACCGCG<br>ACGAGGTGCTGGTGTGCCGCGGTCCGGCCCGGGAGCGTGCGGACGACGCCCCCGCGCGGAGCG<br>CCGGGGACATCTACCGCAACGTCAGCGAGCGCTACCGGTCGGATCACGGAGCCCTGCTGCTCAGC<br>ACCGCCCGGGACCGCTCCACGGGCTTCGCCCAGGAGACCGCCATCCAGGCCACCGGCCGTTATGT<br>GGCGCAGCAGGCCGCCCGGGACGGCTGGGTCATCTCCGGGACGCCCCGGCTGGGACAGCTGGA<br>CAAACTGGTCGAGGCGGCCCGCGACACCTGGTCGGCGCGCTGGTGCACCGGCCGACTGGAGGC<br>GGTGATCGAGGAGGAGCGGCCACGGGTCCTGGCCTTCTCCGTTCCCTTCTTCAGCCAAGTGGTCC<br>CCACCCTCGTCCTGACCACCCTGCTCAAGCAGCGCCGGCCGGACCTGAGGATCGCGCTCGGTGG<br>GCCGACCGTGCAGATGTGGGCGGCCCTTCTGCTGCGCCGCACCGAGGCTGCCCGCTCGGTGGAC<br>CACTGGTGCCTCGGCCACGGCGAGGACTTCCTCACGAACATCCTGCCGCCGCGATCGGCAGGCG<br>CGGCGGCGTCGGTCCGACAGCCGACTCCCCTGCCCGCACACGCCGCGGAGGGCCTGCGGGACG<br>CCTTCGTGCTCAACGATCAGCCGATGCCGGACTTCGGCCAGTTCGACTTCGCCGACTACAGCAACC<br>AGGCCCACCAGTTCCCCTATCGCCTCACCGTCGGCTGCTACTGGGGCAGATGCACGTTCTGCTCGT<br>ACGGCAACCGCTACCGCGACGCCCGCGCTTTCCAGCAGATCCACCCCGACACCGCGGCCGACCA<br>CCTGATCGCGCTCTCCGAACGGCTCGGCATCACCGATGTGGCGGTGGCCGACGAGAACACCGGGC<br>TGCGGCATCTGCTGCGGGTCATGGAGGCGGTACGCCGCAGGGGCGCCGAGCTGACGTTCCGGGT<br>CCGGGCGCGGCTGGAACCGGAGCTCGCCGACCCCGGCTTCTGTCGTCGGCTGCGGGAGTCGGG<br>CTGTGTGCAGCTCTCGACGGGATTCGAGACCGCCCGTCAGGAGATCCTCGACTCCCTGGGCAAGG<br>GACAGGACGCGGCGCACGCCGAACGCGCGGTGCTCAACCTCACCCGTGCGGGCATCGTCACCAA<br>CCTGTCCTTCATGGACGGCTATGACCATCCGAACGCCGTCGACGGCTACCACGACACCGTCGGTGT<br>GATCCAGCGGCACCCGGCGGAGCTGGGCCTGGACACCATGCAGCTCGTGGTGGCCGAACCCGGC<br>AGCTACCTGTGGGCGCAACGAGAGCGGCCCGACGACGAGCCTCTGGTCACCAACGACGGTCTGG<br>CGTTCGCCGCGGGACGGGTGGGTGGAGCGGTCCTGGACGACACCGCGACCGAGGCCGCCAGGC<br>AGCGACTGCTGCGGATGACCGTCGAGGCGGTCCCGGACGCCGAACGGGCCGGCCGGCCGGACC<br>TGCCGCACCGTGGCGGCTCGCCGCCCTCCGAGGACCGGGCGGCCCACCGGCGGCCCGCGCCGG<br>GGCCGGCGCCGGCGGCCCGCCCGCGCTTCGCGGTCGCCCTGGAGCAGGTGGGCGGCCGCTGGT<br>TCCTGGCGGACGTGGCCTGGCCCCGGATGGCAGCCGTGCCTCCTGCCCTCGCCCCCGACCGGGA<br>CGGCCGGTTCACGGCCGACGACCCGGCCACCCACCGCTGGCTGCACCGGATGCTCGACAAACGC<br>CTGCTGGTGCCGGATCCGTCGGCGCCCGACGCCCTCGAAGGGAGGAGTGCGTGA
	AA	MKHHHHHHHHGGENLYFQGMIDALLVMPPVSEAVQFPYLALPQLTAAWTARGHTVRALDLNLEYRDEVL<br>VCRGPARERADDAPARSAGDIYRNVSERYRSDHGALLLSTARDRSTGFAQETAIQATGRYVAQQAARDG<br>WVISGTPRLGQLDKLVEAARDTWSARWCTGRLEAVIEEERPRVLAFSVPFFSQVVPTLVLTTLLKQRRPD<br>LRIALGGPTVQMWAALLLRRTEAARSVDHWCLGHGEDFLTNILPPRSAGAAASVRQPTPLPAHAAEGLR<br>DAFVLNDQPMPDFGQFDFADYSNQAHQFPYRLTVGCYWGRCTFCSYGNRYRDARAFQQIHPDTAADH<br>LIALSERLGITDVAVADENTGLRHLLRVMEAVRRRGAELTFRVRARLEPELADPGFCRRLRESGCVQLST<br>GFETARQEILDSLGKGQDAAHAERAVLNLTRAGIVTNLSFMDGYDHPNAVDGYHDTVGVIQRHPAELGL<br>DTMQLVVAEPGSYLWAQRERPDDEPLVTNDGLAFAAGRVGGAVLDDTATEAARQRLLRMTVEAVPDAE<br>RAGRPDLPHRGGSPPSEDRAAHRRPAPGPAPAARPRFAVALEQVGGRWFLADVAWPRMAAVPPALAP<br>DRDGRFTADDPATHRWLHRMLDKRLLVPDPSAPDALEGRSA
pMAL-c6t::*Ps_bipD*	nt	ATGAAAATCCACCATCACCACCACCACGAAGAAGGTAAACTGGTAATCTGGATTAACGGCGATAAAG<br>GCTATAACGGTCTCGCTGAAGTCGGTAAGAAATTCGAGAAAGATACCGGAATTAAAGTCACCGTTGA<br>GCATCCGGATAAACTGGAAGAGAAATTCCCACAGGTTGCGGCAACTGGCGATGGCCCTGACATTATC<br>TTCTGGGCACACGACCGCTTTGGTGGCTACGCTCAATCTGGCCTGTTGGCTGAAATCACCCCGGAC<br>AAAGCGTTCCAGGACAAGCTGTATCCGTTTACCTGGGATGCCGTACGTTACAACGGCAAGCTGATTG<br>CTTACCCGATCGCTGTTGAAGCGTTATCGCTGATTTATAACAAAGATCTGCTGCCGAACCCGCCAAAA<br>ACCTGGGAAGAGATCCCGGCGCTGGATAAAGAACTGAAAGCGAAAGGTAAGAGCGCGCTGATGTTC<br>AACCTGCAAGAACCGTACTTCACCTGGCCGCTGATTGCTGCTGACGGGGGTTATGCGTTCAAGTATG<br>AAAACGGCAAGTACGACATTAAAGACGTGGGCGTGGATAACGCTGGCGCGAAAGCGGGTCTGACCT<br>TCCTGGTTGACCTGATTAAAAACAAACACATGAATGCAGACACCGATTACTCCATCGCAGAAGCAGC<br>CTTTAATAAAGGCGAAACAGCGATGACCATCAACGGCCCGTGGGCATGGTCCAACATCGACACCAG<br>CAAAGTGAATTATGGTGTAACGGTACTGCCGACCTTCAAGGGTCAACCATCCAAACCGTTCGTTGGC<br>GTGCTGAGCGCAGGTATTAACGCCGCCAGTCCGAACAAAGAGCTGGCAAAAGAGTTCCTCGAAAAC<br>TATCTGCTGACTGATGAAGGTCTGGAAGCGGTTAATAAAGACAAACCGCTGGGTGCCGTAGCGCTG

AAGTCTTACGAGGAAGAGTTGGTGAAAGATCCGCGTATTGCCGCCACTATGGAAAACGCCCAGAAA
GGTGAAATCATGCCGAACATCCCGCAGATGTCCGCTTTCTGGTATGCCGTGCGTACTGCGGTGATCA
ACGCCGCCAGCGGTCGTCAGACTGTCGATGAAGCCCTGAAAGACGCGCAGACTAATTCGAGCTCG
AACAACAACAACAATAACAATAACAACAACCTCGGGGAGAACCTGTACTTCCAGATGCTGATGGGCA
TGGAAAATTCAGATGCAATGATAGTTTTTCCTCCTCTAACTGAAGCCAGGCTCTTTCCATATCTAAGTT
TACCTATGATTACTAGTTTTTTGAGGAACAAGGGAATGAGTGTAAGTCAAATTGATTTAAATATTGAATT
ATGCCATACCCTCTTTAGTGAAGATTGTTTAGCTGAATATGTGAGCATCAATGAAAACGGAAGTAAAGA
CTTAAAATTGATCTATAAAGTCGAAATGGCTAAATACTTATACAAAGAACAAAAGCAACTTTACAATAAT
GTATTCATTGAGAAAAGATCTTCTGATTCATTGAAGTATGACGTTCGGTTGGTGAGACAAGGCATAGA
ACTATTACTATTAAATTCGTTTTTAAAACTTGAGATTACCTCATTGGAGGAAATCCTTGAATTGGTAAGG
AATTTTAGTTGGAAAAAAAGTGATATTGCAACAAAGGTTTTATACGAAAATATCAAAGAAAAAATTCTTA
ACGATAAGCCCAAAATCTTCGCGATTTCTATTGCATATTATAGCCAAATATTACCTTCCCTTTTAATATGC
AAGTGGATTAGAGAACTTTCCCCTAATACTCACATTATTCTGGGTGGACAACAAATCATGATTCGTCAA
TCATCTTTCTTAAGCCTAAATGGATTAAATCAATTTGTGGATTCATTAGGAATATCAGCAGGTGAAGAAA
CATTATTCATGTTAGACAGATATTTGAAAAACGATTGCCAAATCGGGAAGGTGCCGGATATTGTTTGGC
TTAATGAGAAAAATGGTGAAAATATTCCATCTAAGTCTGCATATAGGATCACCGACGCCTTACCACCTG
ACTTTTCTGATTTACCGTACAAAAACTATTTAGATGAAGAAGTGCATATGTCTTTGATTACATGTGTAGG
GTGTTACTGGGGACGATGTACTTTTTGTTCATATGGTAATAGATCCAGAAAAGAAAAAAGTTATCAGCA
AAAAACGGCAAGGCAAGTAGCAAACGAGTGCGAGGATATCATTAACAAATACGGGGTTAATAGAATTA
ATTTCATAGATGAGAATACTAATTTGAGGCTTGTCGTCAATGCAGTCAAAATACTTAAATCCAGAGGAT
ATGAAATTAATTTCAGTACACGCAACCGTCTTGAGAATGTGTTGTTAGACGCTGAATTTTGTTTTGAGC
TAAGTAATTTAGGTTGTATTTTAATGTCTGTTGGCTATGAAACCAATTCTCAAAGGATTTTAGATTCCTT
GGATAAGGGAGTGCAATCTAGTAATTATCAACAGATAATAGATAACTTACATAATGCCAATATCACGCTG
CGAATGTCCATAATTGGTGGCTTACCTGGTGAAACCGAGGATGAAATTAAATGTTCTGAGGAATTTTT
ACTTAAAAATCAAGATAAGATTGGCATCGACGTCATGCAAATGCTCGTTTTAGAACCAGGTACCTATAT
TTATGAAGATACAAATAATCCTGATATCCATATTCAATCAAGCAAGAATCTAAGAGGAAACAAATTACTC
AATTATGGGATGGGTAGAATGGGAGCTACATTTCAATACTCTGACGGAAAAACGTTTGAAGAAAAGCT
GAATAGATTCTTGCAATTGCACAAAAATGTCAACCCCCAAAAGAATGATGAGCTACCTCCTGATAAATA
CAAAACGGTAGGACAGGATATCACTTCAAATACCTTATTGATAAACCCATGGACTAAAATCATTAAGTTA
GATAGGACGTATATCATGGATTTTGTATGGCAAAGAGTCTTCTTAGTGCCAGAATCCATAGAAGTATTC
GGAAACACACTGATAATTAGCAAGGCTGACGATAAAAAGTACCTTGAGTATTTTGTCGAAAAAGGTGT
GATTAAGCTTAAATAA

AA MKIHHHHHHEEGKLVIWINGDKGYNGLAEVGKKFEKDTGIKVTVEHPDKLEEKFPQVAATGDGPDIIFWA
HDRFGGYAQSGLLAEITPDKAFQDKLYPFTWDAVRYNGKLIAYPIAVEALSLIYNKDLLPNPPKTWEEIPAL
DKELKAKGKSALMFNLQEPYFTWPLIAADGGYAFKYENGKYDIKDVGVDNAGAKAGLTFLVDLIKNKHMN
ADTDYSIAEAAFNKGETAMTINGPWAWSNIDTSKVNYGVTVLPTFKGQPSKPFVGVLSAGINAASPNKEL
AKEFLENYLLTDEGLEAVNKDKPLGAVALKSYEEELVKDPRIAATMENAQKGEIMPNIPQMSAFWYAVRTA
VINAASGRQTVDEALKDAQTNSSSNNNNNNNNNNLGENLYFQMLMGMENSDAMIVFPPLTEARLFPYL
SLPMITSFLRNKGMSVSQIDLNIELCHTLFSEDCLAEYVSINENGSKDLKLIYKVEMAKYLYKEQKQLYNN
VFIEKRSSDSLKYDVRLVRQGIELLLLNSFLKLEITSLEEILELVRNFSWKKSDIATKVLYENIKEKILNDKPKI
FAISIAYYSQILPSLLICKWIRELSPNTHIILGGQQIMIRQSSFLSLNGLNQFVDSLGISAGEETLFMLDRYLK
NDCQIGKVPDIVWLNEKNGENIPSKSAYRITDALPPDFSDLPYKNYLDEEVHMSLITCVGCYWGRCTFCS
YGNRSRKEKSYQQKTARQVANECEDIINKYGVNRINFIDENTNLRLVVNAVKILKSRGYEINFSTRNRLEN
VLLDAEFCFELSNLGCILMSVGYETNSQRILDSLDKGVQSSNYQQIIDNLHNANITLRMSIIGGLPGETEDE
IKCSEEFLLKNQDKIGIDVMQMLVLEPGTYIYEDTNNPDIHIQSSKNLRGNKLLNYGMGRMGATFQYSDG
KTFEEKLNRFLQLHKNVNPQKNDELPPDKYKTVGQDITSNTLLINPWTKIIKLDRTYIMDFVWQRVFLVPE
SIEVFGNTLIISKADDKKYLEYFVEKGVIKLK*

pHis$_8$-TEV::*Ps_bipD* nt ATGAAACACCACCACCACCACCACCACCACGGTGGTGAGAATCTTTATTTTCAGGGAATGGAAAATT
CAGATGCAATGATAGTTTTTCCTCCTCTAACTGAAGCCAGGCTCTTTCCATATCTAAGTTTACCTATGA
TTACTAGTTTTTTGAGGAACAAGGGAATGAGTGTAAGTCAAATTGATTTAAATATTGAATTATGCCATAC
CCTCTTTAGTGAAGATTGTTTAGCTGAATATGTGAGCATCAATGAAAACGGAAGTAAAGACTTAAAATT
GATCTATAAAGTCGAAATGGCTAAATACTTATACAAAGAACAAAAGCAACTTTACAATAATGTATTCATT
GAGAAAAGATCTTCTGATTCATTGAAGTATGACGTTCGGTTGGTGAGACAAGGCATAGAACTATTACT
ATTAAATTCGTTTTTAAAACTTGAGATTACCTCATTGGAGGAAATCCTTGAATTGGTAAGGAATTTTAGT
TGGAAAAAAAGTGATATTGCAACAAAGGTTTTATACGAAAATATCAAAGAAAAAATTCTTAACGATAAG
CCCAAAATCTTCGCGATTTCTATTGCATATTATAGCCAAATATTACCTTCCCTTTTAATATGCAAGTGGAT
TAGAGAACTTTCCCCTAATACTCACATTATTCTGGGTGGACAACAAATCATGATTCGTCAATCATCTTT
CTTAAGCCTAAATGGATTAAATCAATTTGTGGATTCATTAGGAATATCAGCAGGTGAAGAAACATTATTC
ATGTTAGACAGATATTTGAAAAACGATTGCCAAATCGGGAAGGTGCCGGATATTGTTTGGCTTAATGA
GAAAAATGGTGAAAATATTCCATCTAAGTCTGCATATAGGATCACCGACGCCTTACCACCTGACTTTTC
TGATTTACCGTACAAAAACTATTTAGATGAAGAAGTGCATATGTCTTTGATTACATGTGTAGGGTGTTAC
TGGGGACGATGTACTTTTTGTTCATATGGTAATAGATCCAGAAAAGAAAAAAGTTATCAGCAAAAAACG
GCAAGGCAAGTAGCAAACGAGTGCGAGGATATCATTAACAAATACGGGGTTAATAGAATTAATTTCATA
GATGAGAATACTAATTTGAGGCTTGTCGTCAATGCAGTCAAAATACTTAAATCCAGAGGATATGAAATT
AATTTCAGTACACGCAACCGTCTTGAGAATGTGTTGTTAGACGCTGAATTTTGTTTTGAGCTAAGTAAT
TTAGGTTGTATTTTAATGTCTGTTGGCTATGAAACCAATTCTCAAAGGATTTTAGATTCCTTGGATAAGG
GAGTGCAATCTAGTAATTATCAACAGATAATAGATAACTTACATAATGCCAATATCACGCTGCGAATGTC
CATAATTGGTGGCTTACCTGGTGAAACCGAGGATGAAATTAAATGTTCTGAGGAATTTTTACTTAAAAA
TCAAGATAAGATTGGCATCGACGTCATGCAAATGCTCGTTTTAGAACCAGGTACCTATATTTATGAAGA
TACAAATAATCCTGATATCCATATTCAATCAAGCAAGAATCTAAGAGGAAACAAATTACTCAATTATGGG
ATGGGTAGAATGGGAGCTACATTTCAATACTCTGACGGAAAAACGTTTGAAGAAAAGCTGAATAGATT
CTTGCAATTGCACAAAAATGTCAACCCCCAAAAGAATGATGAGCTACCTCCTGATAAATACAAAACGG
TAGGACAGGATATCACTTCAAATACCTTATTGATAAACCCATGGACTAAAATCATTAAGTTAGATAGGAC
GTATATCATGGATTTTGTATGGCAAAGAGTCTTCTTAGTGCCAGAATCCATAGAAGTATTCGGAAACAC
ACTGATAATTAGCAAGGCTGACGATAAAAAGTACCTTGAGTATTTTGTCGAAAAAGGTGTGATTAAGCT
TAAATAA

AA MKHHHHHHHHGGENLYFQGMENSDAMIVFPPLTEARLFPYLSLPMITSFLRNKGMSVSQIDLNIELCHTL
FSEDCLAEYVSINENGSKDLKLIYKVEMAKYLYKEQKQLYNNVFIEKRSSDSLKYDVRLVRQGIELLLLNSF
LKLEITSLEEILELVRNFSWKKSDIATKVLYENIKEKILNDKPKIFAISIAYYSQILPSLLICKWIRELSPNTHIILG
GQQIMIRQSSFLSLNGLNQFVDSLGISAGEETLFMLDRYLKNDCQIGKVPDIVWLNEKNGENIPSKSAYRI
TDALPPDFSDLPYKNYLDEEVHMSLITCVGCYWGRCTFCSYGNRSRKEKSYQQKTARQVANECEDIINK
YGVNRINFIDENTNLRLVVNAVKILKSRGYEINFSTRNRLENVLLDAEFCFELSNLGCILMSVGYETNSQRI
LDSLDKGVQSSNYQQIIDNLHNANITLRMSIIGGLPGETEDEIKCSEEFLLKNQDKIGIDVMQMLVLEPGTY
IYEDTNNPDIHIQSSKNLRGNKLLNYGMGRMGATFQYSDGKTFEEKLNRFLQLHKNVNPQKNDELPPDK
YKTVGQDITSNTLLINPWTKIIKLDRTYIMDFVWQRVFLVPESIEVFGNTLIISKADDKKYLEYFVEKGVIKLK
*

pACYC-Duet::*His*$_6$-*Sg_bipEF*

nt

MCS1:
ATGGGCAGCAGCCATCACCATCATCACCACAGCCAGGATCCGATGGAGAAACTCGGACTCGGACTG
GGGATGGACCTGGTCTGGGGGGAACGCATCGGCTTCGACAAGACCGGCTCCGGCCGTCCGACCG
ACCAGGTGGCCGCCTTCCTCGAGCGCAACGCCCATGCCTACGACTACATGTTCGTGGCCTTCCAGC
CCATCGACTACGGCCCGCTCGCTCCCGAGCGCTACGTCCCGGCGTACGACCGGCTCTTCGAGCTG
TTCGGAGCACGGCGCCCACTCGCCTTCCACCACACGCTGCTCAACACCGGCAGCCCGGAGGACTA
CGAGCGGGCCGCGATTGCCGACTTCACCAACGCGCTCATCGAGCGGTACGGATTCCGGTGGGTCA
TCGAGGACCTCGGCATCTGGTCCCTGGCCGGCCGCAGCCTGCCCTACCCCATGCCGCCGGTGCTC
ACCACGGAGGGCCTGCGGCGGTGCGTGAGCGGCGTCGCCGACTGGGTGCGGCGGCTGCGAGCC
CCCCTGTCCGTGGAGTTCCCCGGCTTCACCGAGGGTGGCAGCTTCCTCGTCGGCGAGCTGGACG
CCTTCTCCTTCTACGACACGGTGATCCGCGAGACCGGCGCCCTGGCGACCATCGACATCGGTCACA
TCCTCGCCTACCAGTGGCTGAAGGGACGCACTGGGGAGCGGATGTTCGAAGGGCTGGAGGCGCT
GCCCCTGGACCGCTGCCACGAAGTGCATCTGTCGGGGTGTCAGATCGTCGACGGACGCTTTCGCG
ACCTGCACCACGGCGTGCTGCTCGACGAACAGCTCACCTTGCTCGAGCACCTGCTGCCGCTGATG
CCGAACCTGGCCGGAGTCACCTACGAGGACCCCAAGTTCACTGCCGAGGGAGAGCTGGTGCCCAA
GTCGCGGCCCAACGCGGAACGGCTGTTCACCCTCGTCCGTTCCTGGAAGGAGGACGACGCCCGT
GCAGCGTGA

MCS2:
ATGGCAGATCTCAATTGGATATCGGCCGGCCACGCGATCGCTGACGTCGTGCAGCGTGAAGCCCTC
AGGCACGACCGCATCCTGCGGGTGCTGGACCGCCTGCTGTACGACAAGGACTTCCGCACCGCGTT
CGCCGAGGACGGCCCCGCCGGCGCACGCGTCGCGCTCGACGAGGACCTCCTCGACGCGTTCGAC
CGGGTGGACGTGCACGAACTGGCACTGGTGGGACGCAACATCCGCTCCGAGGTCGTCTCCGGCG
GCACCGGTACCGGGCCCGGCCTCAAGGGGTCCTTCCCCCGGACGCTGGACGCGCTGCGGGAAGG
GCGCCACGCCCCGGTGAACGACGTGGCCGAGGCGTTCATCGCCTCGGCGGCCTTCCAGGAGTTC
CGGGACGTCCCCTTCTCGCCCCGGGGCCGGGGCAGGACCCTGCCCGAGTGCTTCCACCGCTTCA
TGGCCGCACGGCCCGCCGACCTCGACCCTTCCGGCGAACTGGAGCCGCTCGTCCACCACGAGGC
GGCCGCCGCCGTCACCCGAGCGGTCGCCACCGGCGCCCACGCCACCTTCGATGTGGGACTGCGT
GACATGGCCTTCCACGGGGACGTGCTGTGCGGGTTCCGGGAGTACGCCGAGGCGCCCGCCGCAT
GGCAACTGAAACCGACGATGTTCCTGGCCGGAGCCGGACGCTGCGTGATCGGGCCGGCCCGCCG
TCCGCTGTTCGACGCCCTGACCAGCCTCCTCGACGGCCGCCCCGACGCGCTCACGCCGTCCGTC
CGGGCCTCGCTCGAGGACCGCCTCAGCTCCTGGGGGCTGCGATGA

AA

MCS1:
MGSSHHHHHHSQDPMEKLGLGLGMDLVWGERIGFDKTGSGRPTDQVAAFLERNAHAYDYMFVAFQPI
DYGPLAPERYVPAYDRLFELFGARRPLAFHHTLLNTGSPEDYERAAIADFTNALIERYGFRWVIEDLGIWS
LAGRSLPYPMPPVLTTEGLRRCVSGVADWVRRLRAPLSVEFPGFTEGGSFLVGELDAFSFYDTVIRETG
ALATIDIGHILAYQWLKGRTGERMFEGLEALPLDRCHEVHLSGCQIVDGRFRDLHHGVLLDEQLTLLEHLL
PLMPNLAGVTYEDPKFTAEGELVPKSRPNAERLFTLVRSWKEDDARAA*

MCS2:
MADLNWISAGHAIADVVQREALRHDRILRVLDRLLYDKDFRTAFAEDGPAGARVALDEDLLDAFDRVDVH
ELALVGRNIRSEVVSGGTGTGPGLKGSFPRTLDALREGRHAPVNDVAEAFIASAAFQEFRDVPFSPRGR
GRTLPECFHRFMAARPADLDPSGELEPLVHHEAAAAVTRAVATGAHATFDVGLRDMAFHGDVLCGFREY
AEAPAAWQLKPTMFLAGAGRCVIGPARRPLFDALTSLLDGRPDALTPSVRASLEDRLSSWGLR*

pACYC-Duet::*His*$_6$-*MBP*-*Sg_bipEF*

nt

MCS1:
ATGAAAATCCACCATCACCACCACCACGAAGAAGGTAAACTGGTAATCTGGATTAACGGCGATAAAG
GCTATAACGGTCTCGCTGAAGTCGGTAAGAAATTCGAGAAAGATACCGGAATTAAAGTCACCGTTGA
GCATCCGGATAAACTGGAAGAGAAATTCCCACAGGTTGCGGCAACTGGCGATGGCCCTGACATTATC
TTCTGGGCACACGACCGCTTTGGTGGCTACGCTCAATCTGGCCTGTTGGCTGAAATCACCCCGGAC
AAAGCGTTCCAGGACAAGCTGTATCCGTTTACCTGGGATGCCGTACGTTACAACGGCAAGCTGATTG
CTTACCCGATCGCTGTTGAAGCGTTATCGCTGATTTATAACAAAGATCTGCTGCCGAACCCGCCAAAA
ACCTGGGAAGAGATCCCGGCGCTGGATAAAGAACTGAAAGCGAAAGGTAAGAGCGCGCTGATGTTC
AACCTGCAAGAACCGTACTTCACCTGGCCGCTGATTGCTGCTGACGGGGGTTATGCGTTCAAGTATG
AAAACGGCAAGTACGACATTAAAGACGTGGGCGTGGATAACGCTGGCGCGAAAGCGGGTCTGACCT
TCCTGGTTGACCTGATTAAAAACAAACACATGAATGCAGACACCGATTACTCCATCGCAGAAGCAGC
CTTTAATAAAGGCGAAACAGCGATGACCATCAACGGCCCGTGGGCATGGTCCAACATCGACACCAG
CAAAGTGAATTATGGTGTAACGGTACTGCCGACCTTCAAGGGTCAACCATCCAAACCGTTCGTTGGC
GTGCTGAGCGCAGGTATTAACGCCGCCAGTCCGAACAAAGAGCTGGCAAAAGAGTTCCTCGAAAAC
TATCTGCTGACTGATGAAGGTCTGGAAGCGGTTAATAAAGACAAACCGCTGGGTGCCGTAGCGCTG
AAGTCTTACGAGGAAGAGTTGGTGAAAGATCCGCGTATTGCCGCCACTATGGAAAACGCCCAGAAA
GGTGAAATCATGCCGAACATCCCGCAGATGTCCGCTTTCTGGTATGCCGTGCGTACTGCGGTGATCA
ACGCCGCCAGCGGTCGTCAGACTGTCGATGAAGCCCTGAAAGACGCGCAGACTAATTCGAGCTCG
AACAACAACAACAATAACAATAACAACAACCTCGGGGAGAACCTGTACTTCCAGATGCTGATGGGCA
TGGAGAAACTCGGACTCGGACTGGGGATGGACCTGGTCTGGGGGGAACGCATCGGCTTCGACAAG
ACCGGCTCCGGCCGTCCGACCGACCAGGTGGCCGCCTTCCTCGAGCGCAACGCCCATGCCTACG
ACTACATGTTCGTGGCCTTCCAGCCCATCGACTACGGCCCGCTCGCTCCCGAGCGCTACGTCCCGG
CGTACGACCGGCTCTTCGAGCTGTTCGGAGCACGGCGCCCACTCGCCTTCCACCACACGCTGCTC
AACACCGGCAGCCCGGAGGACTACGAGCGGGCCGCGATTGCCGACTTCACCAACGCGCTCATCGA
GCGGTACGGATTCCGGTGGGTCATCGAGGACCTCGGCATCTGGTCCCTGGCCGGCCGCAGCCTGC
CCTACCCCATGCCGCCGGTGCTCACCACGGAGGGCCTGCGGCGGTGCGTGAGCGGCGTCGCCGA
CTGGGTGCGGCGGCTGCGAGCCCCCCTGTCCGTGGAGTTCCCCGGCTTCACCGAGGGTGGCAGC
TTCCTCGTCGGCGAGCTGGACGCCTTCTCCTTCTACGACACGGTGATCCGCGAGACCGGCGCCCT
GGCGACCATCGACATCGGTCACATCCTCGCCTACCAGTGGCTGAAGGGACGCACTGGGGAGCGGA
TGTTCGAAGGGCTGGAGGCGCTGCCCCTGGACCGCTGCCACGAAGTGCATCTGTCGGGGTGTCAG
ATCGTCGACGGACGCTTTCGCGACCTGCACCACGGCGTGCTGCTCGACGAACAGCTCACCTTGCT
CGAGCACCTGCTGCCGCTGATGCCGAACCTGGCCGGAGTCACCTACGAGGACCCCAAGTTCACTG
CCGAGGGAGAGCTGGTGCCCAAGTCGCGGCCCAACGCGGAACGGCTGTTCACCCTCGTCCGTTC
CTGGAAGGAGGACGACGCCCGTGCAGCGTGA

MCS2:
ATGGCAGATCTCAATTGGATATCGGCCGGCCACGCGATCGCTGACGTCGTGCAGCGTGAAGCCCTC
AGGCACGACCGCATCCTGCGGGTGCTGGACCGCCTGCTGTACGACAAGGACTTCCGCACCGCGTT
CGCCGAGGACGGCCCCGCCGGCGCACGCGTCGCGCTCGACGAGGACCTCCTCGACGCGTTCGAC
CGGGTGGACGTGCACGAACTGGCACTGGTGGGACGCAACATCCGCTCCGAGGTCGTCTCCGGCG
GCACCGGTACCGGGCCCGGCCTCAAGGGGTCCTTCCCCCGGACGCTGGACGCGCTGCGGGAAGG
GCGCCACGCCCCGGTGAACGACGTGGCCGAGGCGTTCATCGCCTCGGCGGCCTTCCAGGAGTTC
CGGGACGTCCCCTTCTCGCCCCGGGGCCGGGGCAGGACCCTGCCCGAGTGCTTCCACCGCTTCA
TGGCCGCACGGCCCGCCGACCTCGACCCTTCCGGCGAACTGGAGCCGCTCGTCCACCACGAGGC
GGCCGCCGCCGTCACCCGAGCGGTCGCCACCGGCGCCCACGCCACCTTCGATGTGGGACTGCGT

GACATGGCCTTCCACGGGGACGTGCTGTGCGGGTTCCGGGAGTACGCCGAGGCGCCCGCCGCAT
GGCAACTGAAACCGACGATGTTCCTGGCCGGAGCCGGACGCTGCGTGATCGGGCCGGCCCGCCG
TCCGCTGTTCGACGCCCTGACCAGCCTCCTCGACGGCCGCCCCGACGCGCTCACGCCGTCCGTC
CGGGCCTCGCTCGAGGACCGCCTCAGCTCCTGGGGGCTGCGATGA

AA

MCS1:
MKIHHHHHHEEGKLVIWINGDKGYNGLAEVGKKFEKDTGIKVTVEHPDKLEEKFPQVAATGDGPDIIFWA
HDRFGGYAQSGLLAEITPDKAFQDKLYPFTWDAVRYNGKLIAYPIAVEALSLIYNKDLLPNPPKTWEEIPAL
DKELKAKGKSALMFNLQEPYFTWPLIADGGYAFKYENGKYDIKDVGVDNAGAKAGLTFLVDLIKNKHMN
ADTDYSIAEAAFNKGETAMTINGPWAWSNIDTSKVNYGVTVLPTFKGQPSKPFVGVLSAGINAASPNKEL
AKEFLENYLLTDEGLEAVNKDKPLGAVALKSYEEELVKDPRIAATMENAQKGEIMPNIPQMSAFWYAVRTA
VINAASGRQTVDEALKDAQTNSSSNNNNNNNNNNLGENLYFQMLMGMEKLGLGLGMDLVWGERIGFD
KTGSGRPTDQVAAFLERNAHAYDYMFVAFQPIDYGPLAPERYVPAYDRLFELFGARRPLAFHHTLLNTGS
PEDYERAAIADFTNALIERYGFRWVIEDLGIWSLAGRSLPYPMPPVLTTEGLRRCVSGVADWVRRLRAPL
SVEFPGFTEGGSFLVGELDAFSFYDTVIRETGALATIDIGHILAYQWLKGRTGERMFEGLEALPLDRCHEV
HLSGCQIVDGRFRDLHHGVLLDEQLTLLEHLLPLMPNLAGVTYEDPKFTAEGELVPKSRPNAERLFTLVR
SWKEDDARAA*

MCS2:
MADLNWISAGHAIADVVQREALRHDRILRVLDRLLYDKDFRTAFAEDGPAGARVALDEDLLDAFDRVDVH
ELALVGRNIRSEVVSGGTGTGPGLKGSFPRTLDALREGRHAPVNDVAEAFIASAAFQEFRDVPFSPRGR
GRTLPECFHRFMAARPADLDPSGELEPLVHHEAAAAVTRAVATGAHATFDVGLRDMAFHGDVLCGFREY
AEAPAAWQLKPTMFLAGAGRCVIGPARRPLFDALTSLLDGRPDALTPSVRASLEDRLSSWGLR*

pACYC-Duet::*His6-MBP-Ps_bipEF*

nt

MCS1:
ATGAAAATCCACCATCACCACCACCACGAAGAAGGTAAACTGGTAATCTGGATTAACGGCGATAAAG
GCTATAACGGTCTCGCTGAAGTCGGTAAGAAATTCGAGAAAGATACCGGAATTAAAGTCACCGTTGA
GCATCCGGATAAACTGGAAGAGAAATTCCCACAGGTTGCGGCAACTGGCGATGGCCCTGACATTATC
TTCTGGGCACACGACCGCTTTGGTGGCTACGCTCAATCTGGCCTGTTGGCTGAAATCACCCCGGAC
AAAGCGTTCCAGGACAAGCTGTATCCGTTTACCTGGGATGCCGTACGTTACAACGGCAAGCTGATTG
CTTACCCGATCGCTGTTGAAGCGTTATCGCTGATTTATAACAAAGATCTGCTGCCGAACCCGCCAAAA
ACCTGGGAAGAGATCCCGGCGCTGGATAAAGAACTGAAAGCGAAAGGTAAGAGCGCGCTGATGTTC
AACCTGCAAGAACCGTACTTCACCTGGCCGCTGATTGCTGCTGACGGGGGTTATGCGTTCAAGTATG
AAAACGGCAAGTACGACATTAAAGACGTGGGCGTGGATAACGCTGGCGCGAAAGCGGGTCTGACCT
TCCTGGTTGACCTGATTAAAAACAAACACATGAATGCAGACACCGATTACTCCATCGCAGAAGCAGC
CTTTAATAAAGGCGAAACAGCGATGACCATCAACGGCCCGTGGGCATGGTCCAACATCGACACCAG
CAAAGTGAATTATGGTGTAACGGTACTGCCGACCTTCAAGGGTCAACCATCCAAACCGTTCGTTGGC
GTGCTGAGCGCAGGTATTAACGCCGCCAGTCCGAACAAAGAGCTGGCAAAAGAGTTCCTCGAAAAC
TATCTGCTGACTGATGAAGGTCTGGAAGCGGTTAATAAAGACAAACCGCTGGGTGCCGTAGCGCTG
AAGTCTTACGAGGAAGAGTTGGTGAAAGATCCGCGTATTGCCGCCACTATGGAAAACGCCCAGAAA
GGTGAAATCATGCCGAACATCCCGCAGATGTCCGCTTTCTGGTATGCCGTGCGTACTGCGGTGATCA
ACGCCGCCAGCGGTCGTCAGACTGTCGATGAAGCCCTGAAAGACGCGCAGACTAATTCGAGCTCG
AACAACAACAACAATAACAATAACAACAACCTCGGGGAGAACCTGTACTTCCAGATGCTGATGGGCA
TGAGCACAGTCTATCAGAAAATAAAATTTCCGAATGTAAAGGAAAAACTGGGGCTTGGTTTAGGCATG
GATTTACCTTGGGGGGAAGATATAGGTTTTTCCAGCAAAGGGGACCATGACGACATCACGCCTAAGA
TGAAAGGTTTTTTTCAGAATTATAAAGACGAGTTTAATTATATTTTCTTTGCTTTTCAACCGAAAAATAGA
AGTGTTTTAAAAGCCGAGGACTACTTTGATGCCTATGATAGATTATTTGAAGCCAATCCACATCTAAAG
GCGAGAGCATTCCATCAAACGATTTTAAATATGGGGGCTACCGAGTATTACAAAAAAACAGAAATCATA
GAATTCACAAATAAGATTATAGAAAGATATGACATAAGATGGATCGTAGAGGATCTTGGCCTGTGGTCA
ATAAAAGGGAAAACCGTCCCTTTCCCTTTACCACCATATATGACACCAGCAGGATTGGAAGCTTGTAT
TAAGAACATCAACGAATACCAATCTGAACTGGCTGTTCCAGTTTCAGTTGAATTTCCTGGATTCACTG
AAGGTACTAACTTCTTTATTGGGGAAATAAATGGATTTGATTATTTCAGAACATTGGTCGAAGAGACAA
ACTCCCCTATCACTATAGATATTGGTCATATCCTTAGTTATCAATGGCTGTTAGGGAATACAAATGAAAA
GATGTTCAATGGTTTAGAAAAATTGCCTTTTGAGAACTGTTTTGAATTGCATCTTTCAGGTTGCCAAAT
TATTAAAGGGAAATTCAGAGATTTGCATCATGGAGTTTTAATGGATGAACAAATTCAGCTTTTAGATTAT
TTGCTTCCTTTATGCCCTAATTTAAAAGCCATTACATATGAAGACCCTAAATATACAGATGAAGGAATAT
TAATTCCGAAGTCACAGAAAAATTATCAAAGAATGAAGGAGGCAGTTGAAAAATGGCAAATGATTTAA

MCS2:
ATGGCAGATCTCAATTGGATATCGGCCGGCCACGCGATCGCTGACGTCGGTACCCTCGAGTCTGGT
ATGGCAAATGATTTAAAACCAAAGAATTATTCACTAGACGAGTTACTGTACAATTTATTATATGATTACCA
ATATAGAAATAACTTTTTAAATGATGAGTTTAATGAATTGAATTTATCAGCTGATAATTTAAATCACATAAA
AACAATAGATAAAGAAGAGCTTGTAGCAACGGCTACAACGATTGTAAGAAATTTAATGAGTGGAAATAT
TGAACATAAAGGTGGCTTAAGAACTTCTTTTCCCGGTGTTTTTCAGGCTTTAGAAATCCTTGAAACCG
ATATAACGCTGTTAATGCATAAGTTCCTGGCGTCCAAACATTTCGAGTGCTACATGGAATTGCCCTATG
CAGGGGAGGGTACTTGTATCGAGGAAGCGTTTTACAATTATTTGTCTGAAAACGAAGAATTTATCCTT
GCGGCTGATAATAATCATTTACTATTAAAACACGAATTTTTAAATGCCATTTTATCTATACTAACAGTCAA
TAAACACCCATTTTTCAGAATAGATAGTGACCTGGTTAAAAATAATGGCCATATTTACTATGCGTACCAA
ACATACTCCAAAGAAATATCCGAGGCACTGAGTGGAAAAAAAGTAGCTAGTGATTCCGAAAAGGTTAT
ATGGCTATATGCAGCGACCGAAAAAAATTTAATTAGGGGCCCTATTCATCCAAGTGTATTAGAAATGGT
AGAAATAGGAGGTTCAAGGGAAATAAACCGCCAACAAAAGTTCAATCAGGACCAGATAGACTGGATT
CTAAACTTAGGATTAATCAAGAATGATGGATAA

AA

MCS1:
MKIHHHHHHEEGKLVIWINGDKGYNGLAEVGKKFEKDTGIKVTVEHPDKLEEKFPQVAATGDGPDIIFWA
HDRFGGYAQSGLLAEITPDKAFQDKLYPFTWDAVRYNGKLIAYPIAVEALSLIYNKDLLPNPPKTWEEIPAL
DKELKAKGKSALMFNLQEPYFTWPLIADGGYAFKYENGKYDIKDVGVDNAGAKAGLTFLVDLIKNKHMN
ADTDYSIAEAAFNKGETAMTINGPWAWSNIDTSKVNYGVTVLPTFKGQPSKPFVGVLSAGINAASPNKEL
AKEFLENYLLTDEGLEAVNKDKPLGAVALKSYEEELVKDPRIAATMENAQKGEIMPNIPQMSAFWYAVRTA
VINAASGRQTVDEALKDAQTNSSSNNNNNNNNNNLGENLYFQMLMGMSTVYQKIKFPNVKEKLGLGLG
MDLPWGEDIGFSSKGDHDDITPKMKGFFQNYKDEFNYIFFAFQPKNRSVLKAEDYFDAYDRLFEANPHL
KARAFHQTILNMGATEYYKKTEIIEFTNKIIERYDIRWIVEDLGLWSIKGKTVPFPLPPYMTPAGLEACIKNIN
EYQSELAVPVSVEFPGFTEGTNFFIGEINGFDYFRTLVEETNSPITIDIGHILSYQWLLGNTNEKMFNGLEK
LPFENCFELHLSGCQIIKGKFRDLHHGVLMDEQIQLLDYLLPLCPNLKAITYEDPKYTDEGILIPKSQKNYQ
RMKEAVEKWQMI*

MCS2:
MADLNWISAGHAIADVGTLESGMANDLKPKNYSLDELLYNLLYDYQYRNNFLNDEFNELNLSADNLNHIK
TIDKEELVATATTIVRNLMSGNIEHKGGLRTSFPGVFQALEILETDITLLMHKFLASKHFECYMELPYAGEG
TCIEEAFYNYLSENEEFILAADNNHLLLKHEFLNAILSILTVNKHPFFRIDSDLVKNNGHIYYAYQTYSKEISE
ALSGKKVASDSEKVIWLYAATEKNLIRGPIHPSVLEMVEIGGSREINRQQKFNQDQIDWILNLGLIKNDG*

pMAL-c5x::*Sg_bipG* nt

ATGAAAATCGAAGAAGGTAAACTGGTAATCTGGATTAACGGCGATAAAGGCTATAACGGTCTCGCTGA
AGTCGGTAAGAAATTCGAGAAAGATACCGGAATTAAAGTCACCGTTGAGCATCCGGATAAACTGGAA
GAGAAATTCCCACAGGTTGCGGCAACTGGCGATGGCCCTGACATTATCTTCTGGGCACACGACCGC
TTTGGTGGCTACGCTCAATCTGGCCTGTTGGCTGAAATCACCCCGGACAAAGCGTTCCAGGACAAG
CTGTATCCGTTTACCTGGGATGCCGTACGTTACAACGGCAAGCTGATTGCTTACCCGATCGCTGTTG
AAGCGTTATCGCTGATTTATAACAAAGATCTGCTGCCGAACCCGCCAAAAACCTGGGAAGAGATCCC
GGCGCTGGATAAAGAACTGAAAGCGAAAGGTAAGAGCGCGCTGATGTTCAACCTGCAAGAACCGTA
CTTCACCTGGCCGCTGATTGCTGCTGACGGGGGTTATGCGTTCAAGTATGAAAACGGCAAGTACGA
CATTAAAGACGTGGGCGTGGATAACGCTGGCGCGAAAGCGGGTCTGACCTTCCTGGTTGACCTGAT
TAAAAACAAACACATGAATGCAGACACCGATTACTCCATCGCAGAAGCTGCCTTTAATAAAGGCGAAA
CAGCGATGACCATCAACGGCCCGTGGGCATGGTCCAACATCGACACCAGCAAAGTGAATTATGGTG
TAACGGTACTGCCGACCTTCAAGGGTCAACCATCCAAACCGTTCGTTGGCGTGCTGAGCGCAGGTA
TTAACGCCGCCAGTCCGAACAAAGAGCTGGCAAAAGAGTTCCTCGAAAACTATCTGCTGACTGATGA
AGGTCTGGAAGCGGTTAATAAAGACAAACCGCTGGGTGCCGTAGCGCTGAAGTCTTACGAGGAAGA
GTTGGTGAAAGATCCGCGTATTGCCGCCACTATGGAAAACGCCCAGAAAGGTGAAATCATGCCGAA
CATCCCGCAGATGTCCGCTTTCTGGTATGCCGTGCGTACTGCGGTGATCAACGCCGCCAGCGGTCG
TCAGACTGTCGATGAAGCCCTGAAAGACGCGCAGACTAATTCGAGCTCGAACAACAACAACAATAAC
AATAACAACAACCTCGGGATCGAGGGAAGGATTTCACATATGTCCATGGGCATGAACGATCCGGCGC
AGATCGACGCGGTGGCCTACGCGGGACGGCCCGACGAGATCGACCGGTTCGACCCGGAGCGCTT
CTACCGTGACCACTGGCGCCGCCGCCCCCTGGTACTGCGCGGCTGCGGCAAGACCTTCCTCGCCC
CGGCCCTGGAGTGGTCCGACGTCGAGGCGCTGGAGGCCCGGCTGGACGGCGACACCTCGGGAG
CCGGTGTGCACCGGCGCCCCGACGGCAGCGTGCTCTTCGTCAACCAGGCCCAGCACACCATGCC
CGAACTGGCCGCGTCCTGCACACGGTTGGCGGACCTCATGGGCTGGGAGGAGTGCACCGCCGAC
CTGTCCGTCACCCGCGGCAGCGGCGCGGGGATCGGCTGCCACTTCGATCACAGCGACAACTTCGT
CGTCCAGCAACAGGGCTCGAAGAGCTGGCTGGTAGGTCTTCCGGAACACACCGGCCGCGAGCGG
CAGCGCAAACGCATGCTGGAGACCAAGGGGTTCGTGCCCTCCGGACGGTTCCCCGAGCCCCCGT
GGCAGGTGGTGCTGCACCCCGGCGACGTGCTCTACCTCCCGCTGTTCGCCCCGCACGAGGGCGT
CGAGGATCCGGACGGCCCGGCGCAGGGCTCGGTCAGCGTCTCCTTCTCGTACAACGCGCGCAATG
CGCTGGGCAGCCACCTCAAACCGCTGCTGCGCGCACTGGCCGCGGAAGACGCCTGGTGGCAGCC
GCTGCCCCTGGCTGGGGCCGACCCGGACCGACTCGAAGAGGCACTGCTGCGGGCACTGCGCAAC
GTGACCCGCTCAGCGAAGGGGGAGACATGA

AA

MKIEEGKLVIWINGDKGYNGLAEVGKKFEKDTGIKVTVEHPDKLEEKFPQVAATGDGPDIIFWAHDRFGG
YAQSGLLAEITPDKAFQDKLYPFTWDAVRYNGKLIAYPIAVEALSLIYNKDLLPNPPKTWEEIPALDKELKA
KGKSALMFNLQEPYFTWPLIAADGGYAFKYENGKYDIKDVGVDNAGAKAGLTFLVDLIKNKHMNADTDY
SIAEAAFNKGETAMTINGPWAWSNIDTSKVNYGVTVLPTFKGQPSKPFVGVLSAGINAASPNKELAKEFL
ENYLLTDEGLEAVNKDKPLGAVALKSYEEELVKDPRIAATMENAQKGEIMPNIPQMSAFWYAVRTAVINAA
SGRQTVDEALKDAQTNSSSNNNNNNNNNNLGIEGRISHMSMGMNDPAQIDAVAYAGRPDEIDRFDPER
FYRDHWRRRPLVLRGCGKTFLAPALEWSDVEALEARLDGDTSGAGVHRRPDGSVLFVNQAQHTMPEL
AASCTRLADLMGWEECTADLSVTRGSGAGIGCHFDHSDNFVVQQQGSKSWLVGLPEHTGRERQRKR
MLETKGFVPSGRFPEPPWQVVLHPGDVLYLPLFAPHEGVEDPDGPAQGSVSVSFSYNARNALGSHLKP
LLRALAAEDAWWQPLPLAGADPDRLEEALLRALRNVTRSAKGET*

pMAL-c6t::*Ps_bipG* nt

ATGAAAATCCACCATCACCACCACCACGAAGAAGGTAAACTGGTAATCTGGATTAACGGCGATAAAG
GCTATAACGGTCTCGCTGAAGTCGGTAAGAAATTCGAGAAAGATACCGGAATTAAAGTCACCGTTGA
GCATCCGGATAAACTGGAAGAGAAATTCCCACAGGTTGCGGCAACTGGCGATGGCCCTGACATTATC
TTCTGGGCACACGACCGCTTTGGTGGCTACGCTCAATCTGGCCTGTTGGCTGAAATCACCCCGGAC
AAAGCGTTCCAGGACAAGCTGTATCCGTTTACCTGGGATGCCGTACGTTACAACGGCAAGCTGATTG
CTTACCCGATCGCTGTTGAAGCGTTATCGCTGATTTATAACAAAGATCTGCTGCCGAACCCGCCAAAA
ACCTGGGAAGAGATCCCGGCGCTGGATAAAGAACTGAAAGCGAAAGGTAAGAGCGCGCTGATGTTC
AACCTGCAAGAACCGTACTTCACCTGGCCGCTGATTGCTGCTGACGGGGGTTATGCGTTCAAGTATG
AAAACGGCAAGTACGACATTAAAGACGTGGGCGTGGATAACGCTGGCGCGAAAGCGGGTCTGACCT
TCCTGGTTGACCTGATTAAAAACAAACACATGAATGCAGACACCGATTACTCCATCGCAGAAGCAGC
CTTTAATAAAGGCGAAACAGCGATGACCATCAACGGCCCGTGGGCATGGTCCAACATCGACACCAG
CAAAGTGAATTATGGTGTAACGGTACTGCCGACCTTCAAGGGTCAACCATCCAAACCGTTCGTTGGC
GTGCTGAGCGCAGGTATTAACGCCGCCAGTCCGAACAAAGAGCTGGCAAAAGAGTTCCTCGAAAAC
TATCTGCTGACTGATGAAGGTCTGGAAGCGGTTAATAAAGACAAACCGCTGGGTGCCGTAGCGCTG
AAGTCTTACGAGGAAGAGTTGGTGAAAGATCCGCGTATTGCCGCCACTATGGAAAACGCCCAGAAA
GGTGAAATCATGCCGAACATCCCGCAGATGTCCGCTTTCTGGTATGCCGTGCGTACTGCGGTGATCA
ACGCCGCCAGCGGTCGTCAGACTGTCGATGAAGCCCTGAAAGACGCGCAGACTAATTCGAGCTCG
AACAACAACAACAATAACAATAACAACAACCTCGGGGAGAACCTGTACTTCCAGATGCTGATGGGCT
TGATAGATAAGATACACTATAAACAATTATCACTAACGGATTTCGAAGAAAATTATGCGGGTAAAAAGCC
ACTGATAATAACTGGATTAGTAGATTTATGGCCTTCTAGGGACTGGGATACAGACTATCTACAGTCTAC
TTATGGAGAAAGGGAAATCATCATAAGAAAATCAGGATACGATAAAGAAAAAAAATTTTACAAATCAAA
ACTTGGTAAATTCATCGGTATTTTAAATGATAACCCCGAAAAATTGTATTGTGACTGGCCCTTTTCCATC
ATGGGCAATGAAGATTTAGAGAGTTATTTTGAAACTCCCTCTCTATTCGACCATAATACAATCAGGACC
AAAAATAACAAGAAATTAAAATGGATTTTCCTGGGTTCGGTTGGAACAGGAACCCCAATACACCAGGA
TTTTGAGAAATCACATAATTGGAATGCAGTTGTATTTGGCCAAAAAAAATGGATTTTTTTCAGCCCTGA
TGATACTGAGTATGTGAAAGATTTTAAGCATGATGTATTCGATTTGGATTTAAAGGAGGATCCAAATTTC
AAATATTCCAAGCCCTACATTGTGGAACAGTTTTCTGGAGAATTAATGTACACACCTAAAAGTTGGTG
GCATGCCGTGCGAAATATCGAAACCTCTTTCTCAGTTAGTGAGAATTTTTGGTATAAAGATGAAAAGG
AATAA

AA

MKIHHHHHHEEGKLVIWINGDKGYNGLAEVGKKFEKDTGIKVTVEHPDKLEEKFPQVAATGDGPDIIFWA
HDRFGGYAQSGLLAEITPDKAFQDKLYPFTWDAVRYNGKLIAYPIAVEALSLIYNKDLLPNPPKTWEEIPAL
DKELKAKGKSALMFNLQEPYFTWPLIAADGGYAFKYENGKYDIKDVGVDNAGAKAGLTFLVDLIKNKHMN
ADTDYSIAEAAFNKGETAMTINGPWAWSNIDTSKVNYGVTVLPTFKGQPSKPFVGVLSAGINAASPNKEL
AKEFLENYLLTDEGLEAVNKDKPLGAVALKSYEEELVKDPRIAATMENAQKGEIMPNIPQMSAFWYAVRTA
VINAASGRQTVDEALKDAQTNSSSNNNNNNNNNNLGENLYFQMLMGLIDKIHYKQLSLTDFEENYAGKK
PLIITGLVDLWPSRDWDTDYLQSTYGEREIIIRKSGYDKEKKFYKSKLGKFIGILNDNPEKLYCDWPFSIMG
NEDLESYFETPSLFDHNTIRTKNNKKLKWIFLGSVGTGTPIHQDFEKSHNWNAVVFGQKKWIFFSPDDTE
YVKDFKHDVFDLDLKEDPNFKYSKPYIVEQFSGELMYTPKSWWHAVRNIETSFSVSENFWYKDEKE*

pMAL-c5x::*Sg_bipI* nt

ATGAAAATCGAAGAAGGTAAACTGGTAATCTGGATTAACGGCGATAAAGGCTATAACGGTCTCGCTGA
AGTCGGTAAGAAATTCGAGAAAGATACCGGAATTAAAGTCACCGTTGAGCATCCGGATAAACTGGAA
GAGAAATTCCCACAGGTTGCGGCAACTGGCGATGGCCCTGACATTATCTTCTGGGCACACGACCGC
TTTGGTGGCTACGCTCAATCTGGCCTGTTGGCTGAAATCACCCCGGACAAAGCGTTCCAGGACAAG
CTGTATCCGTTTACCTGGGATGCCGTACGTTACAACGGCAAGCTGATTGCTTACCCGATCGCTGTTG
AAGCGTTATCGCTGATTTATAACAAAGATCTGCTGCCGAACCCGCCAAAAACCTGGGAAGAGATCCC
GGCGCTGGATAAAGAACTGAAAGCGAAAGGTAAGAGCGCGCTGATGTTCAACCTGCAAGAACCGTA

		CTTCACCTGGCCGCTGATTGCTGCTGACGGGGGTTATGCGTTCAAGTATGAAAACGGCAAGTACGA<br>CATTAAAGACGTGGGCGTGGATAACGCTGGCGCGAAAGCGGGTCTGACCTTCCTGGTTGACCTGAT<br>TAAAAACAAACACATGAATGCAGACACCGATTACTCCATCGCAGAAGCTGCCTTTAATAAAGGCGAAA<br>CAGCGATGACCATCAACGGCCCGTGGGCATGGTCCAACATCGACACCAGCAAAGTGAATTATGGTG<br>TAACGGTACTGCCGACCTTCAAGGGTCAACCATCCAAACCGTTCGTTGGCGTGCTGAGCGCAGGTA<br>TTAACGCCGCCAGTCCGAACAAAGAGCTGGCAAAAGAGTTCCTCGAAAACTATCTGCTGACTGATGA<br>AGGTCTGGAAGCGGTTAATAAAGACAAACCGCTGGGTGCCGTAGCGCTGAAGTCTTACGAGGAAGA<br>GTTGGTGAAAGATCCGCGTATTGCCGCCACTATGGAAAACGCCCAGAAAGGTGAAATCATGCCGAA<br>CATCCCGCAGATGTCCGCTTTCTGGTATGCCGTGCGTACTGCGGTGATCAACGCCGCCAGCGGTCG<br>TCAGACTGTCGATGAAGCCCTGAAAGACGCGCAGACTAATTCGAGCTCGAACAACAACAACAATAAC<br>AATAACAACAACCTCGGGATCGAGGGAAGGATTTCACATATGTCCATGGGCCCTGACGGCACCGGT<br>CTGCCTGCCGCCACGCGCGATTCCGGGGACCGGGCCCTGCGCGCGGCGCTTGAGACGGCCCGC<br>GCCGGCCTCGGAGCCCGGGCGCGCATCGTCCGCGACGAATCGGACGGGCTGTGCCGGGTGCGC<br>ATCACGCTCGGACCCGCGCAGCACGGCCGAACGCCGCAATGGGGTGTCGCCGCCTACGACCGCA<br>CCTATGTGGCAGGCGAGGTGGCCTCCGGTGTACGGCAGTTGCTCGACGACCGTGCGCTGGTGGCC<br>CGGCGCCGCCCGCTGCCCGGACCGGTGTCGGGACCGACGCTGCTCACGCCGTCGGCGGCCGGG<br>GTGCTGGTGCACGAGTGCTTCGGGCACACGAGTGAGGCCGACAACTACCTCGTCCACCGCAAGGC<br>ACTCGGCCTGGGACTCGGTGATGTGTGGACCCGAGCACCGCTGACGGTGTGGGACCGCCCGACG<br>GCGCGTCCCTACGCGGGCAGCTATGTCCGTGACGACGAGGGCACCGTGCCCCGTGCCGTCCCGC<br>TGGTGCGTGAGGGCCGCTGGGCCGGGCTGCTCACCGACCGCGCCACCCGCGCGCTGAGCGCGG<br>GACGCAGCACCGGTCACGGGCGGGGAGCGCCCGGGGCGGTGGCGCCTCGGTGCAGCGTGCTCG<br>AGGTGGGCCCGGGAACGCGGTCCGAGACCGAACTGCTCGGCGGCATCGACGACGGCTGGGTCCT<br>GGGGACCGCCATCGGCGGCTTCTCCGTGCGCGAACACCTGATCATCGAGGCGCTGTGGGCCCGC<br>CGGGTGCGGGCGGGCCGCCTGACGGCGGACGTCGTCGGGCCGGTGGCCGTCTGCGCACCCAAG<br>GTGGCGCTGGCCCGGCAGATCACTGCGGTGGGGCGCGAGACACAGGTGCACAGCTCGCCGTACG<br>CCTGTGTCAAGGACAGCCACGAGGTCGGTTCGACCTTGATCAGCCCCTCCCTGCTGCTGGGCCGT<br>TGCGTCCTGCGGCCACTCGGGCAGGTGGAGCGGCTGCTCACCCGGACGTCCGCCCCGGTGCACT<br>GA
	AA	MKIEEGKLVIWINGDKGYNGLAEVGKKFEKDTGIKVTVEHPDKLEEKFPQVAATGDGPDIIFWAHDRFGG<br>YAQSGLLAEITPDKAFQDKLYPFTWDAVRYNGKLIAYPIAVEALSLIYNKDLLPNPPKTWEEIPALDKELKA<br>KGKSALMFNLQEPYFTWPLIAADGGYAFKYENGKYDIKDVGVDNAGAKAGLTFLVDLIKNKHMNADTDY<br>SIAEAAFNKGETAMTINGPWAWSNIDTSKVNYGVTVLPTFKGQPSKPFVGVLSAGINAASPNKELAKEFL<br>ENYLLTDEGLEAVNKDKPLGAVALKSYEEELVKDPRIAATMENAQKGEIMPNIPQMSAFWYAVRTAVINAA<br>SGRQTVDEALKDAQTNSSSNNNNNNNNNNLGIEGRISHMSMGPDGTGLPAATRDSGDRALRAALETAR<br>AGLGARARIVRDESDGLCRVRITLGPAQHGRTPQWGVAAYDRTYVAGEVASGVRQLLDDRALVARRRPL<br>PGPVSGPTLLTPSAAGVLVHECFGHTSEADNYLVHRKALGLGLGDVWTRAPLTVWDRPTARPYAGSYV<br>RDDEGTVPRAVPLVREGRWAGLLTDRATRALSAGRSTGHGRGAPGAVAPRCSVLEVGPGTRSETELLG<br>GIDDGWVLGTAIGGFSVREHLIIEALWARRVRAGRLTADVVGPVAVCAPKVALARQITAVGRETQVHSSPY<br>ACVKDSHEVGSTLISPSLLLGRCVLRPLGQVERLLTRTSAPVH*
pMAL-c5x::*Ps_bipI*	nt	ATGAAAATCGAAGAAGGTAAACTGGTAATCTGGATTAACGGCGATAAAGGCTATAACGGTCTCGCTGA<br>AGTCGGTAAGAAATTCGAGAAAGATACCGGAATTAAAGTCACCGTTGAGCATCCGGATAAACTGGAA<br>GAGAAATTCCCACAGGTTGCGGCAACTGGCGATGGCCCTGACATTATCTTCTGGGCACACGACCGC<br>TTTGGTGGCTACGCTCAATCTGGCCTGTTGGCTGAAATCACCCCGGACAAAGCGTTCCAGGACAAG<br>CTGTATCCGTTTACCTGGGATGCCGTACGTTACAACGGCAAGCTGATTGCTTACCCGATCGCTGTTG<br>AAGCGTTATCGCTGATTTATAACAAAGATCTGCTGCCGAACCCGCCAAAAACCTGGGAAGAGATCCC<br>GGCGCTGGATAAAGAACTGAAAGCGAAAGGTAAGAGCGCGCTGATGTTCAACCTGCAAGAACCGTA<br>CTTCACCTGGCCGCTGATTGCTGCTGACGGGGGTTATGCGTTCAAGTATGAAAACGGCAAGTACGA<br>CATTAAAGACGTGGGCGTGGATAACGCTGGCGCGAAAGCGGGTCTGACCTTCCTGGTTGACCTGAT<br>TAAAAACAAACACATGAATGCAGACACCGATTACTCCATCGCAGAAGCTGCCTTTAATAAAGGCGAAA<br>CAGCGATGACCATCAACGGCCCGTGGGCATGGTCCAACATCGACACCAGCAAAGTGAATTATGGTG<br>TAACGGTACTGCCGACCTTCAAGGGTCAACCATCCAAACCGTTCGTTGGCGTGCTGAGCGCAGGTA<br>TTAACGCCGCCAGTCCGAACAAAGAGCTGGCAAAAGAGTTCCTCGAAAACTATCTGCTGACTGATGA<br>AGGTCTGGAAGCGGTTAATAAAGACAAACCGCTGGGTGCCGTAGCGCTGAAGTCTTACGAGGAAGA<br>GTTGGTGAAAGATCCGCGTATTGCCGCCACTATGGAAAACGCCCAGAAAGGTGAAATCATGCCGAA<br>CATCCCGCAGATGTCCGCTTTCTGGTATGCCGTGCGTACTGCGGTGATCAACGCCGCCAGCGGTCG<br>TCAGACTGTCGATGAAGCCCTGAAAGACGCGCAGACTAATTCGAGCTCGAACAACAACAACAATAAC<br>AATAACAACAACCTCGGGATCGAGGGAAGGATTTCACATATGTCCATGGGCATGAACAATGAATATTT<br>AATATCATATGATTCAACCGTTGAAGGGGTTGTTTCACCAAGGTGCGATGATAATGAAAAGAATAAGTT<br>TCTTCAAGAAGAATTAAATCATTGTTTAAAATTCATTGAACGTGATAGCTATGATCACTTAAGACATTGG<br>AAGTCTTTTAAACTCCGTATTGGATTAAGCAGTTATGAATTAAAGTATTCCATCCTTATAAGGATCTTAAT<br>AGAGACTTCAACATCTATTATTCCAGTATTATGGCAAACAAGAACTAATAATTCACCATCTACTGAATTA<br>ATGGATCATTTTGATGTTATTTTAGCGGAAGTAGAGGCTGCTTTATATGGTCAGCAACTTGAACGCAAG<br>ATTGAAACACAAATTGTGCTTGAGCCATGGACTGGATCTCATTATGTACACGAATGTTTTGGCCATACT<br>TTAGAAGCAGATAATTACCTAGAGTATTTAAAGCCAAATGGAATAACGATGGGATACAAATGGTCAGAA<br>TATAAAATCAACGTATTCGATGACCCGACCATTCCTGGGCAAAATGGCAGTTATTTATTGGATGCGGAA<br>CAGAATGAAACATTTAGGACTCACTTAATAAAGGATGGGATAACCGTAGGTTTGATGCATAATGCAAAA<br>ACCTGTAAGCTCCTAGGCTTACAATCTTCATGTAATGCAAGAAGCGTTGGCATATCTTCACTTGCGAT<br>GCCCAGAATGTCAACAACTTATTTGGATTCTGGCAATATGGAAGTACAGGAGATCATTTCAGGAATCG<br>ACAACGGTGTATATTGCAGAGGTTCCTGGGGTGGGGGATCACTTGGTTTGAATTTTGTAATCCGGCC<br>ATCATACGGTTTGGTCATTAAAAATGGGAAAATAACCAATAAAATACTTAGAAGATTTGATATTAAAGGA<br>AACAAACTTGATTCAGCTAAAGCTATTAAGTCCCTTAGCAATAAACTGCATTTCTTTAATCCGGTTTTC<br>GGCTGTAATAAGAATGGTGAAAATAATCTTAGCGTCACCCAAGGTTCACCTCATATTCATTTTGAAAAT<br>CTAACACTTTATCCCGTACTTTGA
	AA	MKIEEGKLVIWINGDKGYNGLAEVGKKFEKDTGIKVTVEHPDKLEEKFPQVAATGDGPDIIFWAHDRFGG<br>YAQSGLLAEITPDKAFQDKLYPFTWDAVRYNGKLIAYPIAVEALSLIYNKDLLPNPPKTWEEIPALDKELKA<br>KGKSALMFNLQEPYFTWPLIAADGGYAFKYENGKYDIKDVGVDNAGAKAGLTFLVDLIKNKHMNADTDY<br>SIAEAAFNKGETAMTINGPWAWSNIDTSKVNYGVTVLPTFKGQPSKPFVGVLSAGINAASPNKELAKEFL<br>ENYLLTDEGLEAVNKDKPLGAVALKSYEEELVKDPRIAATMENAQKGEIMPNIPQMSAFWYAVRTAVINAA<br>SGRQTVDEALKDAQTNSSSNNNNNNNNNNLGIEGRISHMSMGMNNEYLISYDSTVEGVVSPRCDDNEK<br>NKFLQEELNHCLKFIERDSYDHLRHWKSFKLRIGLSSYELKYSILIRILIETSTSIIPVLWQTRTNNSPSTEL<br>MDHFDVILAEVEAALYGQQLERKIETQIVLEPWTGSHYVHECFGHTLEADNYLEYLKPNGITMGYKWSEY<br>KINVFDDPTIPGQNGSYLLDAEQNETFRTHLIKDGITVGLMHNAKTCKLLGLQSSCNARSVGISSLAMPR<br>MSTTYLDSGNMEVQEIISGIDNGVYCRGSWGGGSLGLNFVIRPSYGLVIKNGKITNKILRRFDIKGNKLDS<br>AKAIKSLSNKLHFFNPVFGCNKNGENNLSVTQGSPHIHFENLTLYPVL*

3 References

[57] D. R. Brademan, N. M. Riley, N. W. Kwiecien, J. J. Coon, *Mol. Cell. Proteomics* **2019**, *18*, S193-S201.
[58] J. R. Whitaker, P. E. Granum, *Anal. Biochem.* **1980**, *109*, 156.
